# Genome-Wide Association Study with Targeted and Non-targeted NMR Metabolomics Identifies 15 Novel Loci of Urinary Human Metabolic Individuality

**DOI:** 10.1371/journal.pgen.1005487

**Published:** 2015-09-09

**Authors:** Johannes Raffler, Nele Friedrich, Matthias Arnold, Tim Kacprowski, Rico Rueedi, Elisabeth Altmaier, Sven Bergmann, Kathrin Budde, Christian Gieger, Georg Homuth, Maik Pietzner, Werner Römisch-Margl, Konstantin Strauch, Henry Völzke, Melanie Waldenberger, Henri Wallaschofski, Matthias Nauck, Uwe Völker, Gabi Kastenmüller, Karsten Suhre

**Affiliations:** 1 Institute of Bioinformatics and Systems Biology, Helmholtz Zentrum München, German Research Center for Environmental Health, Neuherberg, Germany; 2 Institute of Clinical Chemistry and Laboratory Medicine, University Medicine Greifswald, Greifswald, Germany; 3 DZHK (German Center for Cardiovascular Research), partner site Greifswald, Greifswald, Germany; 4 Interfaculty Institute of Genetics and Functional Genomics, University Medicine Greifswald, Greifswald, Germany; 5 Department of Medical Genetics, University of Lausanne, Lausanne, Switzerland; 6 Swiss Institute of Bioinformatics, Lausanne, Switzerland; 7 Research Unit Molecular Epidemiology, Institute of Epidemiology II, Helmholtz Zentrum München, German Research Center for Environmental Health, Neuherberg, Germany; 8 Institute of Genetic Epidemiology, Helmholtz Zentrum München, German Research Center for Environmental Health, Neuherberg, Germany; 9 Institute of Medical Informatics, Biometry and Epidemiology, Chair of Genetic Epidemiology, Ludwig-Maximilians-Universität, Munich, Germany; 10 Institute for Community Medicine, University Medicine Greifswald, Greifswald, Germany; 11 Department of Physiology and Biophysics, Weill Cornell Medical College in Qatar, Doha, Qatar; Stanford University School of Medicine, UNITED STATES

## Abstract

Genome-wide association studies with metabolic traits (mGWAS) uncovered many genetic variants that influence human metabolism. These genetically influenced metabotypes (GIMs) contribute to our metabolic individuality, our capacity to respond to environmental challenges, and our susceptibility to specific diseases. While metabolic homeostasis in blood is a well investigated topic in large mGWAS with over 150 known loci, metabolic detoxification through urinary excretion has only been addressed by few small mGWAS with only 11 associated loci so far. Here we report the largest mGWAS to date, combining targeted and non-targeted ^1^H NMR analysis of urine samples from 3,861 participants of the SHIP-0 cohort and 1,691 subjects of the KORA F4 cohort. We identified and replicated 22 loci with significant associations with urinary traits, 15 of which are new (*HIBCH*, *CPS1*, *AGXT*, *XYLB*, *TKT*, *ETNPPL*, *SLC6A19*, *DMGDH*, *SLC36A2*, *GLDC*, *SLC6A13*, *ACSM3*, *SLC5A11*, *PNMT*, *SLC13A3*). Two-thirds of the urinary loci also have a metabolite association in blood. For all but one of the 6 loci where significant associations target the same metabolite in blood and urine, the genetic effects have the same direction in both fluids. In contrast, for the *SLC5A11* locus, we found increased levels of *myo*-inositol in urine whereas mGWAS in blood reported decreased levels for the same genetic variant. This might indicate less effective re-absorption of *myo*-inositol in the kidneys of carriers. In summary, our study more than doubles the number of known loci that influence urinary phenotypes. It thus allows novel insights into the relationship between blood homeostasis and its regulation through excretion. The newly discovered loci also include variants previously linked to chronic kidney disease (*CPS1*, *SLC6A13*), pulmonary hypertension (*CPS1*), and ischemic stroke (*XYLB*). By establishing connections from gene to disease via metabolic traits our results provide novel hypotheses about molecular mechanisms involved in the etiology of diseases.

## Author Summary

Human metabolism is influenced by genetic and environmental factors defining a person’s metabolic individuality. This individuality is linked to personal differences in the ability to react on metabolic challenges and in the susceptibility to specific diseases. By investigating how common variants in genetic regions (loci) affect individual blood metabolite levels, the substantial contribution of genetic inheritance to metabolic individuality has been demonstrated previously. Meanwhile, more than 150 loci influencing metabolic homeostasis in blood are known. Here we shift the focus to genetic variants that modulate urinary metabolite excretion, for which only 11 loci were reported so far. In the largest genetic study on urinary metabolites to date, we identified 15 additional loci. Most of the 26 loci also affect blood metabolite levels. This shows that the metabolic individuality seen in blood is also reflected in urine, which is expected when urine is regarded as “diluted blood”. Nonetheless, we also found loci that appear to primarily influence metabolite excretion. For instance, we identified genetic variants near a gene of a transporter that change the capability for renal re-absorption of the transporter’s substrate. Thus, our findings could help to elucidate molecular mechanisms influencing kidney function and the body’s detoxification capabilities.

## Introduction

Genome-wide association studies with metabolic traits (mGWAS) investigate the relationship between genetic variance and metabolic phenotypes (metabotypes). In 2008, Gieger *et al*. presented the first mGWAS in serum of 284 individuals [1]. Since then, numerous mGWAS using different analytical platforms and ever larger study populations were published [2–8]. These studies discovered more than 150 genetic loci that associate with blood levels of more than 300 distinct metabolites. We refer to these loci as the genetically influenced metabotypes (GIMs), their ensemble defining the genetic part of human metabolic individuality. Many of the single nucleotide polymorphisms (SNPs) that associate with metabolic traits map to genetic regions coding for enzymes or metabolite transporters that are biochemically linked to the associated metabolites. Moreover, a large number of these GIMs have been previously linked to clinically relevant phenotypic traits. As intermediate traits on the pathways of many disorders, these GIMs have become valuable tools that allow unraveling disease mechanisms on the molecular level [9].

However, so far mGWAS have mostly been limited to studies of serum or plasma metabolite levels, thereby focusing on genetically influenced metabolic homeostasis in blood. Only a few studies investigated urine as a complementary body fluid enabling studies of kidney function and the detoxification capabilities of the human body. In 2011, we published the first mGWAS in urine [10] using proton nuclear magnetic resonance spectroscopy (^1^H NMR) to determine metabolite concentrations in urine of 862 male participants of the SHIP-0 cohort. We identified five genetic loci (*SLC6A20*, *AGXT2*, *NAT2*, *HPD*, and *SLC7A9*) that modulate urinary metabolite levels. While for this study metabolite concentrations were manually derived from the NMR spectra for a targeted set of metabolites, Nicholson *et al*. [5] directly used spectral features as abstract, non-targeted urinary metabolic traits in an mGWAS. Based on data for 211 participants of the MolTWIN and MolOBB studies, the authors identified SNPs at three loci (*ALMS1*/*NAT8*, *AGXT2*, and *PYROXD2*) that were associated with metabolic traits in urine. Two of these loci (*ALMS1*/*NAT8* and *PYROXD2*) were replicated in an NMR-based mGWAS published by Montoliu *et al*. For that study, the authors analyzed non-targeted urinary traits from 265 subjects from the São Paolo metropolitan area [11]. Recently, Rueedi *et al*. [12] reported significant associations of NMR-derived non-targeted urinary traits in ten loci (*ALMS1*/*NAT8*, *ACADL*, *AGXT2*, *NAT2*, *ABO*, *PYROXD2*, *ACADS*, *PSMD9*, *SLC7A9*, and *FUT2*) using data from 835 participants of the CoLaus study, thus bringing the total number of reported urinary GIMs to eleven.

Here, we substantially extend our previous mGWAS with metabolic traits in urine, both in size and in scope. First, we metabolically characterize the urine samples of 3,861 male and female participants of the SHIP-0 study, thereby quadrupling the sample size when compared to previous studies. Second, we combine both targeted and non-targeted NMR-based metabolomics. In this way, we implement the approaches used in the studies by Nicholson *et al*., Montoliu *et al*., and Rueedi *et al*. alongside the targeted metabolomics approach used in our previous study. For an unbiased interpretation of our mGWAS results, we apply tools for evidence-based locus-to-gene mapping and automated assignment of metabolites to non-targeted NMR spectral features. Finally, besides determining the overlap of variants identified in our study with variants previously linked to clinical traits, we specifically investigate the overlap between variants influencing metabolic traits in both urine and blood.

## Results

**Fig 1:**
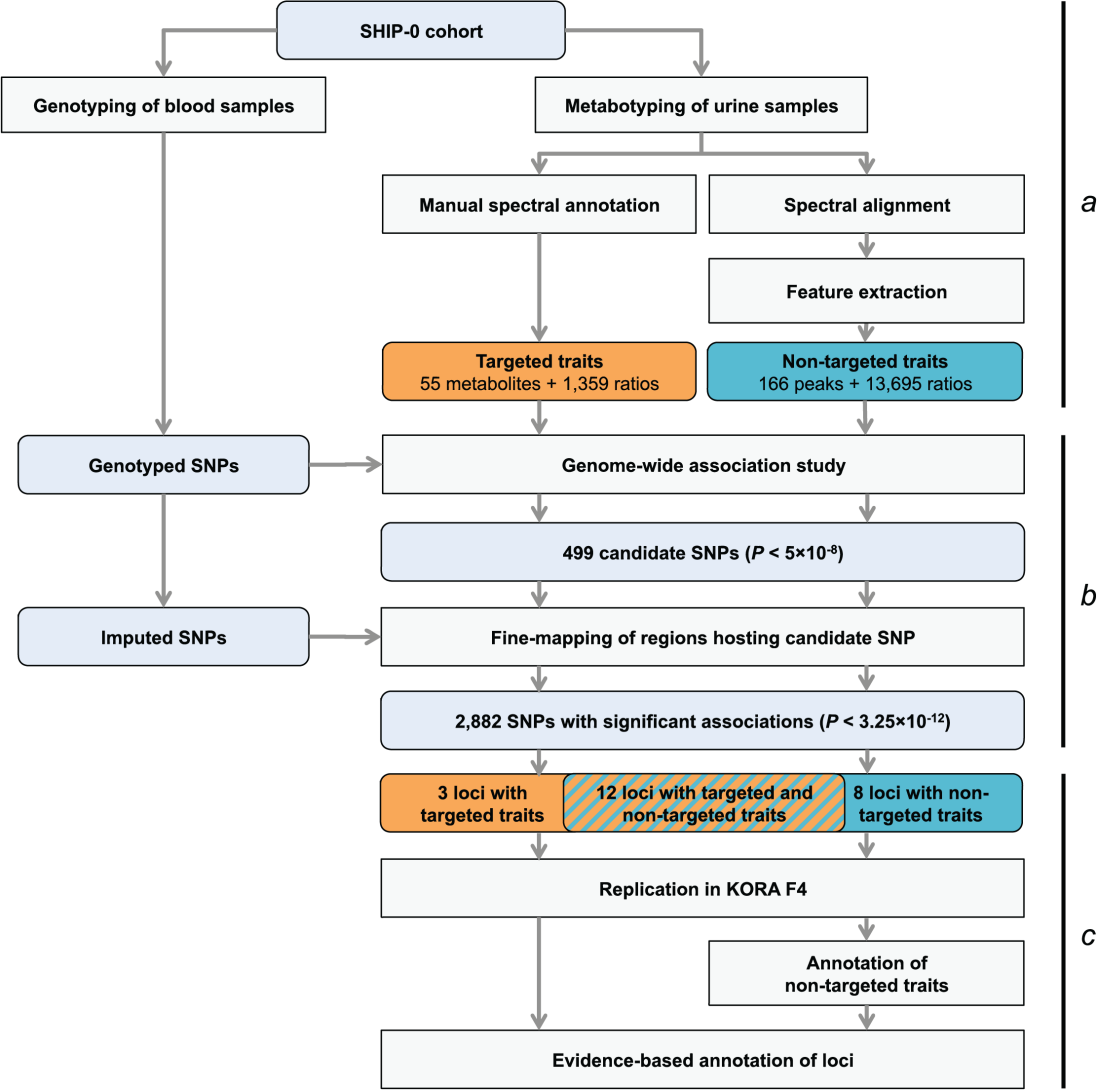
Study design. (a) Genotyping and metabotyping of 3,861 SHIP-0 study participants. One-dimensional ^1^H NMR spectra of the urine samples were recorded to derive targeted and non-targeted metabolic traits. (b) Two-staged mGWAS. First stage: genome-wide association tests using genotyped SNPs and 15,379 targeted and non-targeted traits. Second stage: fine mapping of regions with potentially significant associations using imputed SNPs. (c) Replication and interpretation. Genome-wide significantly associated SNPs were assigned to one of 23 distinct genetic loci. The loci and the significantly associated non-targeted traits were annotated using algorithmic approaches. 22 of the 23 loci could be replicated using genotype and metabotype data from 1,691 KORA F4 participants.

**Fig 2:**
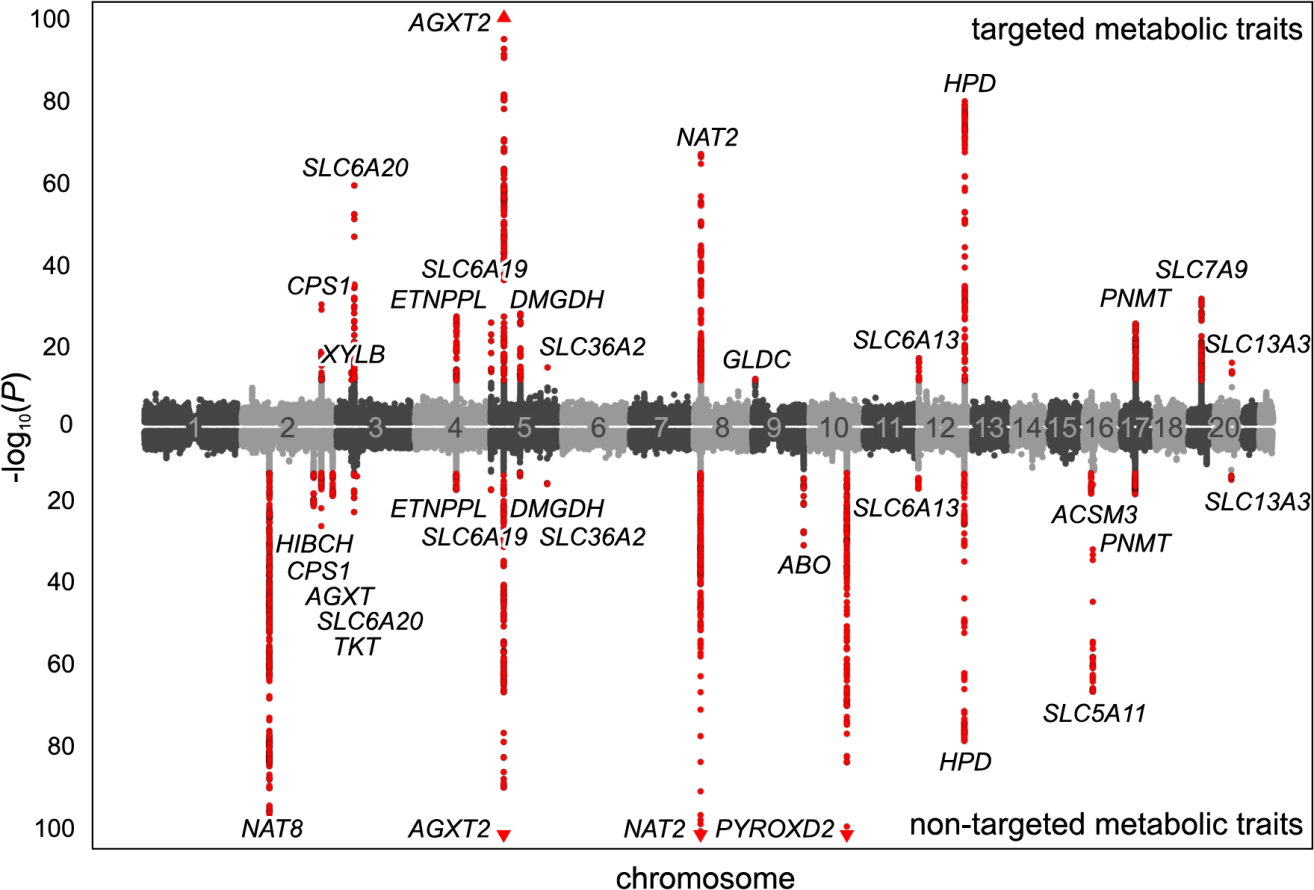
Manhattan plot of genetic associations to targeted and non-targeted traits. SNPs are plotted according to chromosomal location and the-log_10_ transformed *P*-value of the strongest association with targeted traits (top) and non-targeted traits (bottom). In case of associations with ratios, only associations with *P*-gain exceeding 15,180 (targeted metabolic traits) or 138,610 (non-targeted traits) were considered. Associations of genome-wide significance (*P* < 3.25×10^−12^) are plotted in red. Triangles indicate associations with *P* < 1.0×10^−100^. Significant associations within a physical distance of 1 Mb were assigned to a locus labeled after the most likely causative gene (as determined using an evidence-based approach for the identification of candidate genes; see Methods).

**Fig 3:**
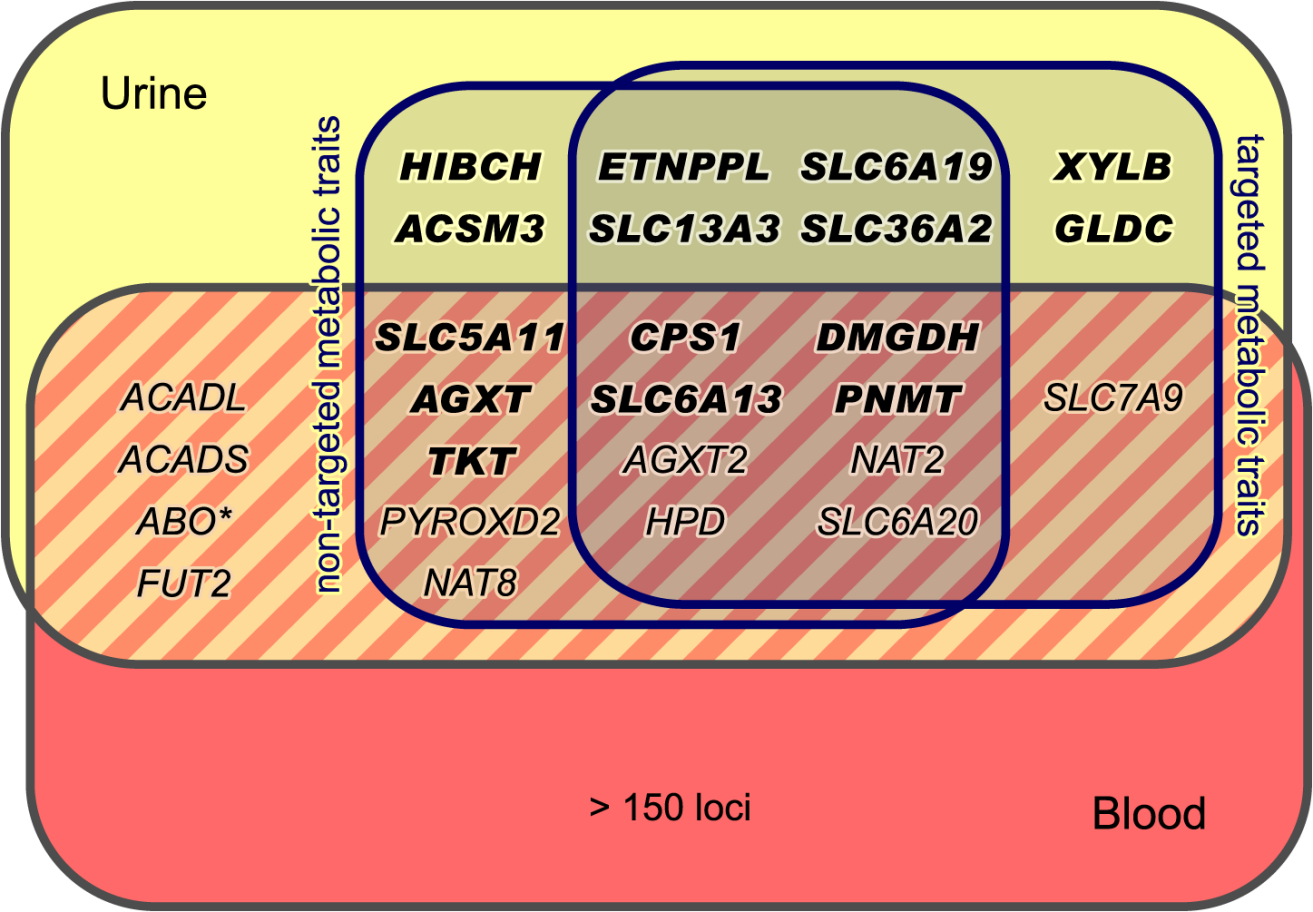
Loci with associated urinary metabolic traits and their overlap with previous mGWAS in blood and urine. We identified and replicated genome-wide significant associations between metabolic traits and genetic variants in 22 genetic loci (named after the most likely causative gene). Three loci could only be identified using targeted metabolic traits, while 7 loci were exclusively discovered with non-targeted traits. 12 loci were identified using both targeted and non-targeted approaches. Loci with hitherto unknown associations with urinary metabolic traits are highlighted (totaling 15). We identified and replicated significant associations in 7 of the 11 loci that were reported in previous mGWAS in urine [5, 10–12]. We also discovered significant associations of the *ABO* locus (marked with an asterisk) with non-targeted traits, but this locus could not be replicated in KORA F4. When compared to previous mGWAS in blood, we find 14 loci that display associations with metabolic traits in both urine and blood [1, 4, 6–8, 14–24].

Our study is based on one-dimensional ^1^H NMR spectra of urine samples from 3,861 genotyped participants in the SHIP-0 cohort (see Methods). For the targeted metabolomics analysis, we manually quantified a set of 60 metabolites in these spectra (Fig 1A). For the non-targeted analysis, we used the same spectra and applied an automated processing algorithm to align the spectra and to perform dimensionality reduction [13]. In the subsequent analysis, we screened the targeted and the non-targeted metabolic traits as well as the pairwise ratios within each trait type for associations with genotyped and imputed variants in a two-step approach (Fig 1B). We identified a total of 23 genetic loci that display significant associations with targeted and/or non-targeted metabolic traits (Fig 2, Tables 1 and 2). All but one of the discovered loci replicated in data from the KORA F4 cohort (N = 1,691). For 15 loci, our study is, to the best of our knowledge, the first to report associations with urinary traits. For 7 of these 15 loci, associations have previously been reported with blood metabolites. Thus, 8 loci are entirely new (Fig 3). Finally, 11 of the 22 replicated loci host significantly associated variants that were previously associated with phenotypes of clinical relevance (Table 3).

**Table 1:** Fifteen genetic loci as discovered in the SHIP-0 data set and their most significant associations to targeted metabolic traits.

Genetic data						Associated metabolic trait						
Locus	Lead SNP	Chr	Position	EA	EAF	Trait or pairwise ratio	N	beta’	*P*	*P*-gain	Replicated	Non-targeted
*CPS1*	rs715	2	211,543,055	C	0.31	glycine/threonine	3,472	0.1409	8.46×10^−31^	9.89×10^10^	●	●
*XYLB*	rs3132440	3	38,395,562	C	0.35	glycolate	3,596	0.0752	2.95×10^−14^		●	–
*SLC6A20*	rs17279437	3	45,814,094	A	0.11	N,N-dimethylglycine/alanine	3,671	-0.2827	8.43×10^−60^	1.36×10^13^	●	●
*ETNPPL*	rs56043887	4	109,705,967	T	0.49	ethanolamine	3,433	-0.0613	7.14×10^−28^		●	●
*SLC6A19*	rs11133665	5	1,188,285	A	0.26	histidine/τ-methylhistidine	2,826	-0.1569	2.40×10^−26^	7.52×10^8^	●	●
*AGXT2*	rs37369	5	35,037,115	T	0.09	3-aminoisobutyrate	3,311	2.2240	2.37×10^−252^		●	●
*DMGDH*	rs6453429	5	78,371,861	T	0.18	N,N-dimethylglycine/betaine	3,611	0.1918	1.52×10^−28^	1.83×10^15^	●	●
*SLC36A2*	rs3846710	5	150,698,806	C	0.17	glycine/citrate	3,816	-0.1225	2.09×10^−15^	5.26×10^4^	●	●
*NAT2*	rs1495743	8	18,273,300	G	0.23	formate/acetate	3,720	0.2688	1.63×10^−67^	2.86×10^7^	●	●
*GLDC*	rs1755615	9	6,643,754	C	0.21	glycine/alanine	3,678	0.0999	1.34×10^−12^	2.83×10^5^	●	–
*SLC6A13*	rs11062102	12	348,785	T	0.36	3-aminoisobutyrate	3,211	-0.1755	1.06×10^−17^		●	●
*HPD*	rs4760099	12	122,318,723	A	0.15	2-hydroxyisobutyrate	3,835	-0.1636	2.20×10^−80^		●	●
*PNMT*	rs7219014	17	37,624,790	A	0.25	histidine/τ-methylhistidine	2,841	-0.1560	4.26×10^−26^	4.27×10^4^	●	●
*SLC7A9*	rs7247977	19	33,358,355	C	0.35	lysine	888	0.3518	9.61×10^−26^		●	–
*SLC13A3*	rs941206	20	45,261,041	C	0.13	succinate/citrate	3,785	0.1245	1.43×10^−16^	1.78×10^8^	●	●

**Chr/Position**: Chromosomal location of the SNP according to the human reference genome (GRCh37). **EA/EAF**: Effect allele and frequency. **Trait or pairwise ratio**: Tested metabolic trait. In case of ratios, the trait that shows the stronger association signal is in the numerator. **N**: Number of samples for which both genotype and phenotype data were available for the tested SNP/metabolic trait pair. **beta’**: beta’ is defined as 10^beta^-1 where beta depicts the relative effect size representing the slope of the regression line in the linear model when using log_10_-scaled metabolic traits and the occurrence of the SNP’s minor allele (coded as 0,1, and 2). Thus, beta’ describes the relative difference per minor allele copy for non-scaled metabolic traits in comparison to the estimated mean of the metabolic trait in the major homozygote test subjects. ***P*-gain**: Defined as min(*P*(M_1_)/*P*(M_1_/M_2_), *P*(M_2_)/*P*(M_1_/M_2_)), where M_1_ and M_2_ represent the two traits of which the ratio M_1_/M_2_ is built. **Replicated**: SNP/metabolic trait pair was replicated in KORA F4 (*P <* 1.32×10^−3^) (S1 Table). **Non-targeted**: SNP or proxy in linkage disequilibrium (LD) is also associated with a non-targeted metabolic trait (*P <* 3.25×10^−12^) (Table 2).

**Table 2:** Twenty genetic loci as discovered in the SHIP-0 data set and their most significant associations to non-targeted metabolic traits.

Genetic data						Associated metabolic trait							
Locus	Lead SNP	Chr	Position	EA	EAF	Trait or pairwise ratio	N	beta’	*P*	*P*-gain	Metabomatching	Replicated	Targeted
*NAT8*	rs10178409	2	73,855,507	T	0.22	2.031 ppm	3,811	0.1988	1.04×10^−95^	–	N-acetylaspartate ^a^	●	–
*HIBCH*	rs13006833	2	191,205,499	G	0.39	1.067 ppm/1.049 ppm	3,552	0.0293	2.06×10^−20^	4.60×10^18^	–	●	–
*CPS1*	rs715	2	211,543,055	C	0.31	3.555 ppm/2.547 ppm	3,536	0.1760	2.80×10^−25^	3.49×10^9^	glycine ^b^	●	●
*AGXT*	rs6748734	2	241,837,452	A	0.29	1.086 ppm	3,800	0.0861	6.94×10^−18^	–	α-ketoisovalerate ^a^	●	–
*SLC6A20*	rs17279437	3	45,814,094	A	0.11	2.916 ppm	3,841	-0.2039	7.88×10^−22^	–	N,N-dimethylglycine	●	●
*TKT*	rs4687717	3	35,282,188	T	0.43	4.094 ppm/7.664 ppm	3.632	0.0476	4.49×10^−13^	6.97×10^6^	gluconate ^a^	●	–
*ETNPPL*	rs7437890	4	109,711,658	C	0.50	3.126 ppm	3,830	-0.0647	2.03×10^−16^	–	ethanolamine	–	●
*SLC6A19*	rs11750211	5	1,183,560	T	0.24	6.877 ppm	3,563	-0.1100	1.92×10^−16^	–	tyrosine ^b^	–	●
*AGXT2*	rs37369	5	35,037,115	T	0.09	1.171 ppm/1.973 ppm	3,828	1.2772	7.48×10^−262^	4.99×10^9^	3-aminoisobutyrate	●	●
*DMGDH*	rs6453427	5	78,361,789	A	0.20	2.916 ppm/5.236 ppm	3,754	0.1566	4.82×10^−13^	4.66×10^5^	N,N-dimethylglycine ^a^	–	●
*SLC36A2*	rs3846710	5	150,698,806	C	0.17	3.555 ppm/2.650 ppm	3,843	-0.1355	5.65×10^−15^	1.11×10^6^	glycine	●	●
*NAT2*	rs35246381	8	18,272,535	C	0.23	2.159 ppm/3.324 ppm	3,841	1.1291	2.70×10^−202^	6.98×10^110^	butyrate ^a^ ^,^ ^b^	●	●
*ABO*	rs550057	9	136,146,597	T	0.29	2.031 ppm/2.049 ppm	3,805	-0.0869	5.85×10^−30^	1.64×10^9^	N-acetylaspartate ^a^	–	–
*PYROXD2*	rs11598867	10	100,146,084	A	0.30	2.854 ppm	3,079	-0.3727	< 1.00×10^−307^	–	trimethylamine ^a^	●	–
*SLC6A13*	rs11062102	12	348,785	T	0.36	1.190 ppm	3,724	-0.1568	4.59×10^−16^	–	3-aminoisobutyrate ^a^	●	●
*HPD*	rs1916333	12	122,458,637	C	0.15	1.345 ppm/7.664 ppm	3,798	-0.1691	6.95×10^−78^	1.64×10^17^	2-hydroxyisobutyrate ^b^	●	●
*ACSM3*	rs11645002	16	20,617,841	A	0.08	1.257 ppm/1.067 ppm	3,800	0.1312	2.59×10^−17^	9.48×10^8^	3-hydroxyisovalerate	●	–
*SLC5A11*	rs17702912	16	25,002,600	T	0.07	3.594 ppm/1.067 ppm	3,860	0.5171	9.24×10^−66^	6.02×10^19^	myo-inositol ^a^	●	–
*PNMT*	rs8069451	17	37,504,933	C	0.24	6.877 ppm	3,842	-0.1093	1.88×10^−17^	–	tyrosine ^b^	–	●
*SLC13A3*	rs941206	20	45,261,041	C	0.13	2.395 ppm/2.650 ppm	3,828	0.1185	6.30×10^−14^	2.13×10^7^	succinate ^b^	●	●

^a^ alternative candidates for metabomatching exist (S1 Fig)

^b^ additional candidates match to other non-targeted traits that also associate with SNP (S1 Fig)

**Chr/Position**: Chromosomal location of the lead SNP according to the human reference genome (GRCh37). **EA/EAF**: Effect allele and frequency. **Trait or pairwise ratio**: Tested metabolic trait (chemical shift). In case of ratios, the trait that drives the association (i.e., shows the stronger association signal) is named first. **N**: Number of samples where both genotype and phenotype data were available for the tested SNP/metabolic trait pair. **beta’**: beta’ is defined as 10^beta^-1 where beta depicts the relative effect size representing the slope of the regression line in the linear model when using log_10_-scaled metabolic traits and the occurrence of the SNP’s minor allele (coded as 0,1, and 2). Thus, beta’ describes the relative difference per minor allele copy for non-scaled metabolic traits in comparison to the estimated mean of the metabolic trait in the major homozygote test subjects. ***P*-gain**: Defined as min(*P*(M_1_)/*P*(M_1_/M_2_), *P*(M_2_)/*P*(M_1_/M_2_)), where M_1_ and M_2_ represent the two traits of which the ratio M_1_/M_2_ is built. **Metabomatching**: Annotation of the non-targeted metabolic trait at the given chemical shift as suggested by metabomatching (S1 Fig). **Replicated**: SNP/metabolic trait pair was replicated in KORA F4 (*P <* 1.32×10^−3^) (S2 Table **)**. **Targeted**: SNP or proxy in LD is also associated with a targeted metabolic trait (*P <* 3.25×10^−12^) (Table 1).

**Table 3:** Twenty-two identified and replicated loci and their overlap with associations to metabolic traits and clinical phenotypes.

Locus		Associated traits					Locus	
Candidate genes	Lead SNPs	Targeted metabolomics [this study]	Non-targeted metabolomics [this study]	Other mGWAS in urine	Other mGWAS in blood	Clinical phenotypes	Comment	Functional match
***NAT8***, *ALMS1*, *DUSP11*, *STAMBP*	rs10178409 ^b^		N-acetylaspartate ^d^ ↗	N-acetylated compounds ↗ [5, 11, 12]	N-acetylornithine ↘ [6, 21, 25], creatinine ↘ [19], 2-aminooctanoic acid (prev. unknown X-12510) ↘ [8, 21], unknown X-11787 ↗ [25], unknown X-12093 ↗ [21], unknown X-13477 ↘ [21]	chronic kidney disease ^e^ [19, 26], glomerular filtration rate ^e^ [27]	*NAT8* is highly expressed in kidney. This gene putatively encodes an N-acetyltransferase. The association of this locus with N-acetylated L-aspartate matches the enzymatic function.	●
***HIBCH***	rs13006833 ^b^		unknown NMR trait (ratio) ↗				*HIBCH* encodes 3-hydroxyisobutyryl-CoA hydrolase. *HIBCH* is linked to *HIBCH* deficiency (MIM 250620), which can lead to neurodegeneration (OrphaNet 88639).	
***CPS1***	rs715 ^a^ ^,^ ^b^	creatine ↗, glycine (+ 8 ratios) ↗	creatine ↗, glycine ↗		N-acetylglycine ↗ [21], betaine ↘ [21], carnitine ↘ [21], creatine ↗ [21, 28], fibriongen ↘ [15], glutaroyl glycine ↗ [6, 14, 21, 22, 24, 25, 28], glycine/PC ae C38:2 ↗ [4], HDL cholesterol ↘ [17], homoarginine ↗ [29], homocysteine ↗[30], pyroglutamine ↘ [21], serine ↗ [21], unknown X-08988 ↗ [21]	chronic kidney disease ^e^ [26], neonatal pulmonary hypertension ^f^ [31]	*CPS1* is highly expressed in liver. It encodes a mitochondrial carbamoyl phosphate synthetase that generates carbamoyl phosphate from ammonia and bicarbonate. *CPS1* deficiency causes Hyperammonemia (MIM 237300). Excess ammonia could be converted to glycine via the glycine cleavage complex (see main text).	●
***AGXT***	rs6748734 ^b^		α-ketoisovalerate ^d^ ↗		unknown X-12556 ↗ [21]		*AGXT* is an alanine-glyoxylate aminotransferase, which is highly expressed in liver. The encoded protein is also highly localized in liver. *AGXT* is linked to type 1 Hyperoxaluria, which can cause renal failure (MIM 259900).	
***XYLB***	rs3132440 ^a^	glycolate ↗				ischemic stroke ^e^ ^,^ ^f^ [32]	*XYLB* likely encodes a xylulokinase that catalyzes D-xylulose to D-xylulose-5-phosphate [33]. The higher glycolate concentration might indicate a switch to an alternative xylulose pathway via phosphofructokinase (*PFK*), as glycolate and related metabolites (glyoxylate, oxalate) are downstream products of the reaction catalyzed by *PFK* (see main text). Metabolic profiles of patients with cerebral infarction show significantly elevated levels of glycolate [34].	●
***SLC6A20***, *CCR1*, *CCR3*, *CCR9*, *LIMD*	rs17279437 ^a^ ^,^ ^b^	N,N-dimethylglycine (+ 10 ratios) ↘	N,N-dimethylglycine ↘	N,N-dimethylglycine/alanine ↘ [10]	pyroglutamine ↗ [21], unknown X-11315 ↘ [21]	Iminoglycinuria ^f^ [35], Hyperglycinuria ^f^ [35]	*SLC6A20* encodes the *SIT1* transporter for imino acids and N-methylated amino acids. *SLC6A20* is linked to Hyperglycinuria and Iminoglycinuria (MIM 138500, MIM 242600).	●
***TKT*** ^c^, *PRKCD*, *GLT8D1*	rs4687717 ^b^		gluconate ^d^ ↗		erythronate/phosphate ↗ [21]		*TKT* encodes a transketolase that connects the pentose phosphate pathway to glycolysis. *PRKCD* is linked to autoimmune diseases (MIM 615559, OrphaNet 300345).	
***ETNPPL*** ^c^, *COL25A1*	rs56043887 ^a^, rs7437890 ^b^	ethanolamine ↘	ethanolamine ↘				*ETNPPL* is highly expressed in brain and liver. The gene encodes an ethanolamine-phosphate-phospholyase. Ethanolamine is the direct precursor of ethanolamine-phosphate (see main text).	●
***SLC6A19*** ^c^, *SLC6A18*	rs11133665 ^a^, rs11750211 ^b^	histidine (+ 3 ratios) ↘, tyrosine (+ 1 ratio) ↘	tyrosine ↘				*SLC6A18* and *SLC6A19* encode transporters for neutral amino acids in kidney. Both genes are linked to Iminoglycinuria [MIM 242600, OrphaNet 42062]; *SLC6A19* is linked to Hyperglycinuria (MIM 138500) and Hartnup disorder (MIM 234500).	
***AGXT2***, *DNAJC21*, *PRLR*, *RAD1*	rs37369 ^a^ ^,^ ^b^	3-aminoisobutyrate ↗	3-aminoisobutyrate ↗	3-aminoisobutyrate ↗ [5, 10, 12]	3-aminoisobutyrate ↗ [28], symmetric/asymmetric dimethylarginine ↗ [20], homoarginine ↘ [29]	heart rate variability ^e^ [20]	*AGXT2* expression is enriched in brain and liver. It catalyzes the biosynthesis of 3-aminoisobutyrate. The non-synonymous variant rs37369 is most likely causative for Beta-aminoisobutyric aciduria (MIM 21010) [10, 36].	●
***DMGDH***, *BHMT*, *ARSB*	rs6453429 ^a^, rs6453427 ^b^	N,N-dimethylglycine (+ 5 ratios) ↗	N,N-dimethylglycine ^d^ ↗		betaine ↘ [21], dimethylglycine ↗ [24, 28], selenium ↗ [18]		*DMGDH* expression is highly enriched in kidney and liver, and so is the gene product. *DMGDH* encodes dimethylglycine dehydrogenase. *DMGDH* deficiency causes fish-like body odor (MIM 605850). In the same locus, *BHMT* catalyzes N,N-dimethylglycine and L-methionine to betaine and homocysteine (EC 2.1.1.5).	●
***SLC36A2***	rs3846710 ^a^ ^,^ ^b^	glycine/citrate ↘	glycine ↘				*SLC36A2* expression is enriched in kidney. The gene encodes a transporter (*PAT2*) for small amino acids (including glycine). Like *SLC6A20*, *SLC36A2* is linked to Hyperglycinuria and Iminoglycinuria (MIM 138500, MIM 242600).	●
***NAT2***, *ASAH1*, *PCM1*	rs1495743 ^a^, rs35246381 ^b^	formate (+ 8 ratios) ↗	formate ↗, butyrate ^d^ ↗, acetylcarnitine ^d^ ↗, N-acetylputrescine ^d^ ↗, τ-methylhistidine ^d^ ↘, 1,3-dimethylurate ^d^ ↘	formate/succinate ↗ [10], unknown NMR trait ↗ [12]	4-acetamidobutanoate ↗ [21], 1-methylurate ↘ [21], 1-methylxanthine ↘ [21], total cholersterol ↗ [17, 37], triglycerides ↗ [17, 37]	bladder cancer^e^ [38, 39], drug response (slow acetylation)^f^ [40–43]	*NAT2* encodes an arylamine N-acetyltransferase. *NAT2* is linked to speed of acetylation (MIM 243400).	●
***GLDC***	rs1755615 ^a^	glycine/alanine ↗					*GLDC* encodes the mitochondrial glycine dehydroxygenase (decarboxylating), which is part of the glycine cleavage system. Mutations in *GLDC* can cause glycine encephalopathy (MIM 605899). *GLDC* expression is enriched in kidney and liver.	●
***PYROXD2***, *HPS1*	rs11598867 ^b^		trimethylamine ^d^ ↘	trimethylamine ↘ [5, 11, 12], unknown NMR trait ↘ [12]	1-methyl-2-piperidine carboxylic acid (prev. unknown X-12092) ↘ [8, 21], caprolactam [16], asymmetric dimethylarginine ↘ [28], dimethylamine ↘ [5], unknown NMR trait ↘ [22], unknown X-12093 ↘ [8, 21]	Hermansky-Pudlak syndrome I ^f^ [44]	*PYROXD2* codes for “pyridine nucleotide-disulphide oxidoreductase domain 2”.	
***SLC6A13***	rs11062102 ^a^ ^,^ ^b^	3-aminoisobutyrate (+ 1 ratio) ↘	3-aminoisobutyrate ^d^ ↘		3-aminoisobutyrate ↘ [28], pyroglutamine ↘ [21]	chronic kidney disease ^e^ [26]	*SLC6A13* is highly expressed in kidney. It encodes *GAT2* that transports betaine, which serves as an osmolyte in kidney, and gamma-aminobutyric acid (GABA). *SLC6A13* might also be a transporter for 3-aminoisobutyrate, which has a similar chemical structure to betaine and GABA.	
***HPD***, *WDR66*, *PSMD9*	rs4760099 ^a^, rs1916333 ^b^	2-hydroxyisobutyrate ↘	2-hydroxyisobutyrate ↘	2-hydroxyisobutyrate ↘ [10, 12]	2-hydroxyisobutyrate ↘ [21]	Tyrosinemia ^f^ [45], Hawkinsuria ^f^ [45], *HPD* deficiency ^f^ [45]	The *HPD* gene product catalyzes 4-hydroxyphenylpyruvate to homogentisate. 2-hydroxyisobutyrate may be related to branched chain amino acid degradation metabolites. *HPD* is linked to type 3 Tyrosinemia (MIM 276710) and Hawkinsuria (MIM 140350). *HPD* is highly expressed and localized in liver and kidney.	
***ACSM3*** ^c^, *ACSM1*	rs11645002 ^b^		3-hydroxyisovalerate ↗				The *ACSM3* gene product (alias *Sa* or *SAH*) is an acyl-CoA synthetase medium-chain family member. “Isobutyrate is the most preferred fatty acid among C2-C6 fatty acids for *Sa* protein.” [46] To lesser extent, *ACSM3* shows substrate specificity for the C5 fatty acid isovalerate [46]. It is estimated that *ACSM3* acts on “acids from C4 to C11 and on the corresponding 3-hydroxy (…) unsaturated acids” (UniProt Q53FZ2). *ACSM3* is suspected to be a risk locus for overweight and hypertension (MIM 145505).	●
***SLC5A11***, *ARHGAP17*, *TNRC6A*	rs17702912 ^b^		myo-inositol ^d^ ↗		myo-inositol ↘ [21], scyllo-inositol ↘ [21]		*SLC5A11* is short for solute carrier family 5 sodium/myo-inositol transporter, member 11 (see main text).	●
***PNMT***, *MED1*, *PPP1RB*, *STARD3*	rs7219014 ^a^, rs8069451 ^b^	histidine (+ 1 ratio) ↘, tyrosine ↘	tyrosine ↘		HDL cholesterol ↘ [17, 37]	rheumatoid arthritis ^e^ [47], reduced *PNMT* activity ^f^ [48]	*PNMT* codes for phenylethanolamine N-methyltransferase and is part of tyrosine metabolism (EC 2.1.1.28).	●
***SLC7A9***, *C19orf40*, *CEP89*, *RHPN2*, *TDRD12*	rs7247977 ^a^	lysine (+ 8 ratios) ↗		lysine/valine ↗ [10], lysine ↗ [12]	homocitrulline ↘ [21], NG-monomethyl-arginine ↘ [28]	chronic kidney disease ^e^ [26]	*SLC7A9* is a transporter for cysteine and neutral and dibasic amino acids. Lysine is a dibasic amino acid and might thus be a substrate of *SLC7A9*. *SLC7A9* is linked to Cystinuria (MIM 220100).	●
***SLC13A3***, *SLC2A10*	rs941206 ^a^ ^,^ ^b^	succinate/citrate ↗	succinate ↗				*SLC13A3* is a high-affinity dicarboxylate transporter that mediates the transport of succinate [49]. It is highly expressed in kidney.	●

^a^ SNP with the strongest association to targeted metabolic traits (“lead SNP”)

^b^ SNP with the strongest association to non-targeted metabolic traits

^c^ manually added to the list of most plausible candidate genes derived by evidence-based selection

^d^ additional candidates match to other non-targeted traits that also associate with the lead SNP

^e^ results from GWAS (*P* < 5.0×10^−8^)

^f^ mutations determined in clinical studies

For each locus, we selected all variants that displayed genome-wide significant association signals to metabolic traits in the SHIP-0 cohort. We added their proxy variants in LD (r^2^ ≥ 0.8; based on 1000 genomes project data [50, 51]). These variant sets were used for the selection of candidate genes and the comparison with association results from other studies. **Candidate genes:** selection of genes based on variant evidence (genes hit or close-by, eQTL, potentially regulatory effects, or missense variants) (S3 Table). Genes with the highest evidence counts are listed. Genes with the most plausible biochemical relation to the associated trait are highlighted in bold typeface. **Associated traits**: Targeted/non-targeted metabolomics: traits that display genome-wide significant association signals in SHIP-0. The arrows indicate whether the trait increases (↗) or decreases (↘) per copy of the effect allele. For non-targeted traits, the most plausible metabolite candidates according to metabomatching are given. **Other mGWAS in urine/blood:** metabolic traits that were previously found to be associated with a locus variant. The arrows indicate the directionality of the effect for the reported effect allele (where available). **Clinical phenotypes:** overlap with variants found to be associated with clinical traits. **Comment**: Gene expression rates were taken from the Illumina Body Map 2.0 (S4 Table). Protein localizations were taken from the Human Protein Atlas (version 12) [52]. For genes linked to clinical traits, we provide OMIM or OrphaNet accession numbers if available. **Functional match**: Indicates which associations exhibit a sound biological link between gene function and the biochemical nature of the associated metabolite(s).

### mGWAS with targeted and non-targeted NMR features

For the targeted metabolomics analysis, the ^1^H NMR spectra were manually annotated to derive absolute metabolite concentrations (out of a panel of 60 compounds) for each sample. For the non-targeted analysis, the same spectra were automatically aligned and processed using the FOCUS package [13]. This resulted in NMR signal intensities at 166 distinct spectral positions (“chemical shifts”) per sample (see Methods). In previous mGWAS, we demonstrated the potential of testing pairwise ratios of metabolite concentrations to boost genetic association signals [1, 4, 6, 8, 10, 21, 53]. Recently, we showed that this approach can also be successfully applied to NMR-based mGWAS with non-targeted features [22]. Thus, we calculated the pairwise ratios of all metabolite concentrations with at least 300 valid data points over all samples (55×54/2 = 1,485 ratios of targeted traits) and NMR signal intensities (166×165/2 = 13,695 ratios of non-targeted traits), respectively (Fig 1A). Out of all 15,401 metabolic features (targeted and non-targeted traits and the ratios thereof), a total of 15,379 features with at least 300 valid data points were screened for genetic associations using 620,456 genotyped autosomal SNPs (Fig 1B). To this end, we computed age- and sex-adjusted linear models under the assumption of additive genetic effects for each SNP-metabolic trait pair. A total of 499 genotyped variants display associations with metabolic traits with *P*-values below 5×10^−8^.

### Fine-mapping of chromosomal regions with associated variants

We used the 499 variants identified in the mGWAS to tag 54 distinct chromosomal regions at a window size of at least 2 Mb (centered to the tag SNPs). We then performed additional association studies using imputed variants (1000 genomes project imputation) in the tagged regions (Fig 1B). We considered associations with a *P*-value below the Bonferroni-adjusted significance threshold of α’ = 5×10^−8^/15,379 = 3.25×10^−12^ to be genome-wide significant. For ratio traits, we also required the *P*-gain to be greater than 1.52×10^4^ for targeted traits and 1.38×10^5^ for non-targeted traits (10 times the number of tested traits [53]). *P*-gain reflects the increase of association strength with the ratio trait when compared to the *P*-values that result from associations with the individual traits buildinging the ratio. A total of 2,882 genotyped or imputed SNPs display association signals below *P* < 3.25×10^−12^ and, in case of ratios, above the imposed *P*-gain threshold (Fig 2). All significantly associated SNPs within a physical distance of 1 Mb were assigned to one of 23 distinct genetic loci. Three loci display significant association signals only when imputed SNPs were used, and 8 loci show significant associations only when pairwise ratios of metabolic traits were considered. Twelve loci show significant associations in both targeted and non-targeted data sets. Three loci are only significantly associated with targeted traits (i.e., quantified metabolite concentrations or ratios thereof), whereas 8 loci are only significantly associated with non-targeted traits (spectral features or ratios thereof) (Fig 3). For each locus, we list the SNP that displays the strongest association signal (lead SNP) and its associated metabolic trait in Tables 1 and 2. In addition, we provide boxplots, regional association plots, and Q-Q plots for each locus in S2 Fig. The summary statistics for all association signals with *P* < 0.05 (*P* < 1×10^−4^ and *P*-gain ≥ 10 for associations with ratios) for each tested SNP can be downloaded from http://www.gwas.eu.

### Systematic assignment of loci to genes and annotation of non-targeted metabolic traits

In general, the biological interpretation of association results from mGWAS requires the mapping of SNPs to candidate genes that are most likely causally linked to the observed changes in the metabotype. Furthermore, non-targeted metabolic traits that exhibit significant association signals have to be assigned to distinct metabolites. In our study, we implemented algorithmic approaches for both the locus-to-gene mapping and the assignment of non-targeted metabolic features.

As the first step in the candidate gene selection, we assigned the significantly associated SNPs to distinct loci using a physical distance threshold of 1Mb. Assigning variants within a locus to one of the covered genes based only on proximity or plausibility ignores haploblock structure and existing regulatory information for the SNPs such as expression quantitative trait loci (eQTL). To take such information into account and to achieve an unbiased selection of candidate genes, we collected evidence for each significantly associated SNP and its proxies in strong linkage disequilibrium (LD) using the SNiPA web server [51]. For each locus, we received a list of candidate genes that are linked to one or more associated variants (or a proxy in LD). Thereby, genes are linked via genomic proximity (i.e., if any of the variants is located within the candidate gene or is in close proximity), via eQTL associations (i.e., if any of the variants is associated with expression levels of the gene in a previous eQTL study), or via regulatory element association (i.e., if any of the variants is contained in a promoter/enhancer/repressor element that is associated with the gene). Moreover, missense variants or known pathogenic variants in the locus are considered to provide additional types of evidence for the linked genes. We finally assigned the locus to the gene with the strongest functional evidence (i.e., the gene showing the highest number of different types of evidences (max. 5) among the candidate genes; see Methods). In case of ambiguous assignments, the gene with the most plausible biological function was chosen. As an example, one locus contains a high number of SNPs with strong associations with non-targeted traits corresponding to N-acetylated compounds. These SNPs cover 12 different genes (see regional association plots in S2 Fig). The gene covered by the highest number of SNPs is *ALMS1*. However, there are 3 more genes in this locus with the same amount of functional evidence count as *ALMS1* (S3 Table). One of these genes is *NAT8*, which encodes an N-acetyltransferase. Since there is a biologically meaningful link between the function of the *NAT8* gene product and the associated metabolic traits, we annotated this locus with *NAT8* as the most likely candidate gene. According to our evidence-based candidate genes assignment approach, the 23 loci map to the genes *NAT8*, *HIBCH*, *CPS1*, *AGXT*, *XYLB*, *SLC6A20*, *TKT*, *ETNPPL*, *SLC6A19*, *AGXT2*, *DMGDH*, *SLC36A2*, *NAT2*, *ABO*, *GLDC*, *PYROXD2*, *SLC6A13*, *HPD*, *ACSM3*, *SLC5A11*, *PNMT*, *SLC7A9*, and *SLC13A3*. S3 Table provides a complete list of candidate genes and the corresponding collected evidences.

For the identification of metabolites underlying non-targeted NMR traits, we used pseudo-spectra that display the strength of associations of a given SNP across the complete NMR spectrum [12, 22]. If the association is strong enough, these “association spectra” often exhibit a striking similarity to the reference NMR spectrum of the underlying metabolite(s). For the present study, we applied the “metabomatching” method introduced by Rueedi *et al*. [12] to perform an automated annotation of the association spectra for each genetic locus of interest. For 19 of the 20 loci that display significant associations with non-targeted traits, metabomatching suggests plausible metabolite candidates matching signals present in the association spectra (S1 Fig). For 10 of these 19 loci (*CPS1*, *SLC6A20*, *ETNPPL*, *SLC6A19*, *AGXT2*, *SLC36A2*, *HPD*, *ACSM3*, *PNMT*, and *SLC13A3*), the match between the association signal and the NMR spectrum of the candidate metabolite (as provided by the Urine Metabolome Database [54]) is strong and unique, which makes the assignment of a metabolite identity to a non-targeted trait unambiguous in these cases.

### Replication

To replicate our findings, we used genotype data and urine samples from participants of the KORA F4 cohort (N = 1,691). From recorded ^1^H NMR spectra of the urine samples, we derived the targeted and non-targeted metabolic traits (metabolite concentrations, NMR spectral features, and the respective pairwise ratios) as for the discovery study. For 14 of the 15 new loci that show significant associations with targeted metabolic traits in the SHIP-0 data set, the top-ranking SNP/metabolic trait association replicates in KORA F4 (S1 Table). For the *SLC7A9* locus, the association with lysine/valine does not replicate, possibly due to the difficulty in annotating lysine from the NMR spectra (> 75% missing values for lysine). However, the second-best, still genome-wide significant association of the tested SNP with valine replicates. For 15 of 20 loci that display significant association signals in the GWAS with non-targeted traits, we were able to replicate the best SNP/NMR trait association or, if this failed, the next, still significant follow-up association (S2 Table). The failure to replicate the remaining 5 loci might be due to the lower sample size in KORA, due to different fasting states of the subjects in the different cohorts, or due to a less perfect alignment of the NMR spectra, since we chose the same FOCUS parameters for aligning SHIP and KORA spectra instead of treating them separately. However, 4 of these 5 loci (*ETNPPL*, *SLC6A19*, *DMGDH*, *PNMT*) show also significant associations in the targeted SHIP-0 data set that replicate in the targeted KORA F4 data set (Table 1). Out of the 23 loci identified in the discovery study, *ABO* is the only locus that could not be replicated using either a targeted or a non-targeted metabolic trait in KORA F4, leaving 22 loci that display stable associations with metabolic traits in urine.

### Overlap with previous mGWAS in urine and blood

We evaluated each identified and replicated locus in the light of previously reported associations with metabolic phenotypes and clinical traits. To this end, we selected all SNPs within a locus for which we found genome-wide significant associations with any urinary metabolic trait in the SHIP-0 cohort. Furthermore, we added all bi-allelic variants from the 1000 genomes project [50] (phase 1, version 3, European ancestry) that are in strong LD to these SNPs (r^2^ ≥ 0.8).

For 15 of the 22 loci, no associations with urinary metabolic traits were reported so far (*HIBCH*, *CPS1*, *AGXT*, *XYLB*, *TKT*, *ETNPPL*, *SLC6A19*, *DMGDH*, *SLC36A2*, *GLDC*, *SLC6A13*, *ACSM3*, *SLC5A11*, *PNMT*, and *SLC13A3*) (Fig 3, Table 3). The remaining 7 loci were already identified in our previous urine mGWAS (*AGXT2*, *HPD*, *SLC7A9*, *SLC6A20*, and *NAT2*) [10] or in the studies by Nicholson *et al*. [5] and Rueedi *et al*. [12] (*NAT8* and *PYROXD2*). For all 7 loci, both trait association and direction of the observed effect are consistent with the results previously published.

We further compared our association results with those of published mGWAS with metabolic traits in blood (*P* < 5×10^−8^), including all studies listed in the NHGRI GWAS catalog [23] and other studies such as the mGWAS by Shin *et al*., which is based on metabolomics data from the KORA F4 and TwinsUK cohorts [1, 4, 6–8, 14–22, 24]. In total, 14 loci show significant associations with metabolic traits both in blood in one of these mGWAS and in urine in our study (Fig 3, Table 3). For 3 of these 14 loci (*SLC6A20*, *PNMT*, *AGXT*), we consider the associated metabolic traits in both media to be unrelated. In 5 cases (*NAT2*, *NAT8*, *PYROXD2*, *SLC7A9*, *TKT*), the genetic association analyses identified different, but related metabolites (i.e., the associated metabolites from urine and blood are either products/substrates of the locus’ candidate gene product, or are biochemically converted within another known enzymatic reaction, or belong to the same metabolite class). In 6 cases, the associations target the same metabolites in urine and blood (*CPS1*, *AGXT2*, *DMGDH*, *SLC6A13*, *HPD*, *SLC5A11*). For 5 of these 6 loci, the direction of the observed effect is the same, whereas for *SLC5A11* (associated with *myo*-inositol), we observe an increase in urinary metabolite concentration per copy of the effect allele, as opposed to decreased levels reported in blood (Table 3). For this locus, we additionally investigated whether the effects seen in blood and urine are directly coupled. To this end, we made use of *myo*-inositol levels (normalized to circulating creatinine) measured through mass spectrometry (MS) in blood serum samples of the same KORA F4 participants [6] that form the replication cohort in this study. The ratio between the urinary *myo*-inositol (this study) and the serum *myo*-inositol levels shows an increase in association strength to the lead SNP in *SLC5A11* (rs17702912) by seven orders of magnitude in comparison to the association of urinary *myo*-inositol alone (*P*_urine_ < 1.95×10^−24^, *P*_blood_ < 1.50×10^−4^, *P*_ratio_ < 2.43×10^−31^).

### Overlap with disease-associated variants and risk genes

For 11 loci, our mGWAS identified significantly associated variants for which either the same variant or a proxy in strong LD was previously reported to be associated with clinical phenotypes according to data from the NHGRI GWAS catalog [23] (*P* < 5×10^−8^), OMIM variation, ClinVar [55], HGMD [56], or dbGaP [57]. Amongst others, these variants have been linked to chronic kidney disease (*NAT8*, *CPS1*, *SLC6A13*, and *SLC7A9*), pulmonary hypertension (*CPS1*), ischemic stroke (*XYLB*), Iminoglycinuria (*SLC6A20*), heart rate variability (*AGXT2*), Hawkinsuria (*HPD*), and pharmacogenomically relevant acetylation phenotypes (*NAT2*) (Table 3). In addition to these 11 loci with disease-associated variants, we found previously discovered connections between clinical traits and the assigned candidate gene for another 7 loci (*HIBCH*, *AGXT*, *SLC6A19*, *DMGDH*, *SLC36A2*, *GLDC*, *ACSM3*).

## Discussion

In this study, we present the largest genome-wide association study with metabolic traits (mGWAS) in urine to date. In addition to quadrupling the sample size compared to previous mGWAS in urine, we analyzed both targeted traits (metabolite concentrations manually derived from NMR spectra) and non-targeted traits (NMR spectral features).

### Fifteen new genetic loci linked to urinary metabolic traits

In total, we identified 23 genetic loci with significant associations between genetic variants and targeted or non-targeted metabolic traits in urine of SHIP-0 participants, 22 of which replicate in the independent KORA F4 cohort. To the best of our knowledge, 15 loci have not been linked to changes in the urine metabolome before. For the remaining 7 loci, our results are in line with the results from previous mGWAS in urine [5, 10, 12] regarding both the associated metabolic traits and the direction of the genetic effects (Table 3).

### Targeted and non-targeted metabolomics are complementary

Though derived from the same NMR spectra, the list of GIMs identified with targeted traits and non-targeted traits partly differ. Of the 22 genetic loci reported in this study, only 12 loci were discovered in both targeted and non-targeted traits, whereas 7 loci show significant associations only with non-targeted traits, and 3 only with targeted traits (Fig 3 and Table 3).

For the data set used in the targeted analysis, the NMR spectra were manually annotated to identify and quantify the metabolites underlying the spectra. Involving human expert knowledge usually allows metabolite identification with very high confidence and yields more precise quantification, especially if signals of multiple metabolites overlap in the NMR spectrum. Furthermore, a manual annotation can to some extent compensate for different experimental and sample conditions, as alignment and pre-processing can be optimized for each spectrum individually. As an example, lysine exhibits characteristic signals in the NMR spectral region between δ = 1.68 and 1.76 ppm, which is often dominated by signals from a variety of additional metabolites, making the annotation of lysine a very difficult task. Thus, while lysine concentrations could be determined for 888 samples of the discovery cohort through manual quantification and yielded a significant genetic association at *SLC7A9*, the non-targeted approach did not capture any association signals for this locus.

However, a manual spectral annotation as performed in the targeted analysis is quite laborious, which limits the number of quantifiable metabolites in large studies. This leads to a bias towards a certain set of metabolites and, as a consequence, significant associations actually present in the NMR data might be missed. Also, a manual annotation in general bears some risk of annotator-induced bias [58]. As an automated method, the non-targeted analysis of spectra has the potential to overcome some of the limitations of targeted analyses. Here, the most prominent example is the *PYROXD2* locus, where SNPs display exceptionally strong associations (*P* < 1.0×10^−307^) to the NMR signal intensities at δ = 2.854 ppm. We could not identify any significant associations within this locus using the targeted data. Thus, we assumed that our set of targeted traits did not cover the metabolite(s) corresponding to these signals. The challenge with genetically associated non-targeted traits lies in the lack of biochemical interpretability. To facilitate the assignment of non-targeted NMR traits to chemical compounds, we applied the metabomatching algorithm introduced by Rueedi *et al*. [12]. In case of *PYROXD2*, metabomatching suggests that the associated NMR signals correspond to trimethylamine. Thereby, the automated method replicates the findings of Nicholson *et al*. [5] where the authors manually annotated the associated signals based on expert knowledge. In case of our mGWAS with targeted traits, trimethylamine was not part of the metabolite panel and thus the association with *PYROXD2* could only be discovered using non-targeted metabolic traits in combination with the automated metabomatching processing. Of course, automated annotation of non-targeted traits also has its limitations: the annotation through metabomatching relies on the association signals that genetic variants display over the NMR spectral range (“association spectra”) as well as on the existence of the relevant reference metabolite spectrum (see Methods and S1 Fig). In some cases, these association spectra are not meaningful enough to allow an unambiguous assignment of non-targeted features to metabolites, or they may be pointing to a metabolite not present in the reference set.

In summary, our study demonstrates that GWAS with NMR-determined metabolic traits can benefit from a combined application of both targeted and non-targeted metabolomics. Our results suggest that a targeted approach is better suited for the annotation of metabolites for which the corresponding NMR signals are in regions with a plethora of other signals as in some cases these signals cannot be resolved through non-targeted methods. Furthermore, genetic associations with targeted traits appear to be more robust, since 5 of the 12 loci that display associations with both targeted and non-targeted traits clearly display stronger association signals in the targeted data set (several orders of magnitude in case of the *SLC6A20* locus; Tables 1 and 2). However, the non-targeted metabolic traits provide a less biased view on the metabolome, which in our case results in additional significantly associated genetic loci.

### Functional metabolomics: from GIMs to testable hypotheses

Fifteen of the 22 identified and replicated loci show a plausible biochemical connection between functionally annotated genes and their associated metabolic traits (Table 3). This is similar to observations from previous mGWAS. For instance, Shin *et al*. reported biologically meaningful links between metabolites and genetic loci for 101 of 145 GIMs [21]. In case of genes with vague functional annotations, gene-metabolite associations from mGWAS provide testable hypotheses for further gene characterization. As an example, Suhre *et al*. experimentally confirmed the mGWAS driven hypothesis of *SLC16A9* being a carnitine transporter [6]. Vice versa, with the help of mGWAS, the chemical structure of a non-targeted metabolic trait was elucidated through the function of the associated gene [8].

Another prominent finding of previous mGWAS is the overlap between disease relevant genetic variants and variants associated with metabolic traits. In the present study, we found 11 loci hosting variants that have previously been linked to clinical phenotypes. This includes associations with the estimated glomerular filtration rate (eGFR) and chronic kidney disease (CKD). Thus, the associated metabolites might, on the one hand, serve as intermediate traits for clinical endpoints. On the other hand, the associations might provide new insights regarding the involvement of specific metabolic pathways in pathomechanisms and the mediation of genetic risk loci through metabolic changes. For all 22 GIMs, we provide information on both the match of gene and metabolite function and the link to clinical traits in Table 3. In the following, we exemplify the value of our results for the characterization of gene functions in the light of clinical phenotypes.

As a first example, we identified significant associations of variants upstream of *ETNPPL* with ethanolamine. Interestingly, at the time when we received the first results from our association studies this gene was named *AGXT2L1* and was assumed to encode an *alanine-glyoxylate-aminotransferase*. Based on this gene annotation, there was no obvious relation to the associated metabolite ethanolamine. In such cases, only dedicated experiments (similar to the one for the carnitine/SLC16A9 association mentioned above [6]) could validate the connection of ethanolamine to the gene product. Meanwhile, Veiga-da-Cunha *et al*. experimentally investigated the locus in an independent study and found that *AGXT2L1* actually encodes an ethanolaminephosphate-phospholyase [59]. As a consequence, *AGXT2L1* now carries the gene symbol *ETNPPL*. As ethanolamine is a direct precursor of ethanolaminephosphate via ethanolamine kinase (EC 2.7.1.28), our finding indeed matches the actual gene function. Besides the functional characterization of this locus, Veiga-da-Cunha *et al*. suggest that the *ETNPPL*-mediated degradation of ethanolaminephosphate balances the concentration of that metabolite in the central nervous system. They concluded that an altered ethanolaminephosphate homeostasis might contribute to mental disorders such as schizophrenia [59]. In line with this hypothesis, the *ETNPPL* expression rate in brain was previously found to be associated with schizophrenia [60]. *ETNPPL* is primarily expressed in brain and liver and the encoded protein is, amongst other tissues, highly localized in the cerebral cortex and the kidney (S4 Table and The Human Protein Atlas [52], http://www.proteinatlas.org/ENSG00000164089/tissue). Our results suggest that an excess of ethanolamine in urine could indicate alterations in ethanolaminephosphate homeostasis linked to a genetically reduced enzymatic activity of *ETNPPL*.

As a second example, we identified significant associations of genetic variants with 2-hydroxyisobutyrate (2-HIBA) in a locus comprising 9 different genes. According to our evidence-based locus to gene assignment, *4-hydroxyphenylpyruvate dioxygenase* (*HPD*) is the most probable effector gene candidate. The association between 2-HIBA and this locus represents a well replicated finding: it was already identified in our previous NMR-based mGWAS in urine [10] and it has meanwhile also been discovered in an MS-based GWAS with blood metabolites [21]. Nonetheless, to the best of our knowledge, there is no obvious, known biological link between 2-HIBA and the *HPD* gene or any of the remaining 8 genes covered by this locus. In the literature, 2-HIBA is often referred to as a secondary metabolite that can be found in urine of humans and rats exposed to the volatile gasoline additives methyl-*tert*-butylether and ethyl-*tert*-butylether [61–63]. However, 2-HIBA has been identified by both MS- and NMR-based methods in almost all serum and urine samples of large human cohorts (e.g. ARIC [25], CoLaus [12], KORA [6, 21], SHIP [10], TasteSensomics [12], and TwinsUK [6, 21]) in relatively high concentrations (~ 40 μM in urine in this study), which suggests sources beyond gasoline for this metabolite (e.g. microbiota [64] or medication [65]). Interestingly, Dai *et al*. recently showed that 2-HIBA is an intermediate for the newly discovered but common 2-hydroxyisobutyrylation of lysine residues of histones [66], thus indicating an endogenous role of 2-HIBA. In this context, it is interesting to note that *SETD1B* is one of the genes within the identified locus on chromosome 12. *SETD1B* is a component of the methyltransferase complex that specifically methylates the lysine-4 residue of histone H3 [67]. This residue is amongst the 63 sites for 2-hydroxyisobutyrylation presented by Dai *et al*. [66]. Thus, one could speculate that in addition to its activity as a histone methylase, *SETD1B* may also be involved in the newly discovered process of histone hydroxyisobutyrylation, a hypothesis that may now be tested by dedicated experiments.

As a third example, we discuss the association of variants in *XYLB* with increased urinary glycolate levels. One of these variants, rs17118, causes an amino acid exchange in the *XYLB* gene product. *XYLB* encodes the enzyme *xylulokinase* [33], which catalyzes the phosphorylation of D-xylulose to D-xylulose-5-phosphate. In humans, the vast majority of D-xylulose is metabolized via *xylulokinase* [33, 68, 69]. However, there is an alternative metabolic pathway in which D-xylulose is metabolized by *phosphofructokinase* (*PFK*) (S3 Fig) [70]. Therein, one of the downstream products is glycolate. Thus, the genetic variants in *XYLB* might reduce the enzymatic activity of *xylulokinase* and thereby cause a shift towards the alternative pathway. Interestingly, the minor allele of rs17118 has been implicated in increased susceptibility for ischemic stroke [32]. Furthermore, Jung *et al*. found a significant association between elevated glycolate levels in plasma and cerebral infarction [34]. In the alternative pathway, glycolate is a precursor of oxalate, whose toxic effect has been demonstrated repeatedly [33, 71]. Very recently, Rao *et al*. postulated that circulating oxalate precipitate might be a potential mechanism for stroke [72]. In this context, the association between the SNP rs17118 and glycolate (identified in our study) suggests that the carriers of this variants have a higher risk of stroke (identified in [32]) possibly via increased levels of glycolate or oxalate through favoring the alternative D-xylulose degradation. Unfortunately, oxalate or any other metabolite in the two D-xylulose degradation pathways are not detected in our metabolomics analysis to further support our hypothesis.

### Extending blood GIMs to urine

In total, 26 genetic loci that associate with urinary metabolic traits are known to date (22 identified or confirmed in this study plus 4 identified in previous studies [5, 11, 12], Fig 3). Of the 26 loci, only 8 loci lack corresponding SNP-metabolite associations in blood, and, based on current mGWAS, represent urine specific hits. All of these 8 loci were first reported in the present study. In case of the 14 loci with overlapping associations between blood and urine in our study (Table 3), 6 target the same metabolite in both media (*CPS1*, *AGXT2*, *DMGDH*, *SLC6A13*, *HPD*, *SLC5A11*). Interestingly, in all but one case (*SLC5A11*) the genetic effect has the same direction in both fluids, thus indicating that urine can be regarded as “diluted plasma” to some extent. For 5 of the 14 loci, we considered the associated metabolic traits in blood and urine to be biochemically related. Here, the metabolites are either products of the enzyme coded by the candidate gene (*NAT8*: N-acetylated compounds), or they are linked through an enzymatic reaction other than the reaction catalyzed by the candidate gene’s product (*NAT2*: 1,3-dimethylurate and 1-methylurate [73]; *PYROXD2*: trimethylamine and dimethylamine [EC 1.5.8.2]; *SLC7A9*: lysine and homocitrulline [EC 2.1.3.8]), or they belong to the same metabolite class (*TKT*: gluconate and erythronate are aldonates). The observed associations of related but different metabolites in blood and urine may be indicative either for biochemical conversions before excretion, or simply be a result of differences in the composition of the metabolite panels covered by the various mGWAS. In case of the remaining 3 loci, we find no direct biochemical or metabolic relationship between the metabolites in both media, since *AGXT* associates with an unknown compound in blood, *PNMT* associates with amino acids in urine and HDL cholesterol in blood, and *SLC6A20* targets loosely related amino acid derivatives.

As an example for parallel effects in blood and urine, we identified an association between variants in the *Carbamoyl-Phosphate Synthetase 1* (*CPS1*) gene and elevated urinary glycine levels. The strongest associated SNP rs715 was also identified in previous mGWAS with higher glycine concentrations in blood [14, 21, 24]. This variant has been highlighted previously as a putative regulator of *CPS1* expression [74–76]. Furthermore, the second strongest glycine-associated SNP rs1047891 causes a non-synonymous mutation (Thr>Asn) in the C-terminal domain of the *CPS1* polypeptide, which hosts the binding site for the allosteric activator N-acteyl-L-glutamate (NAG) [77]. Both SNPs are therefore potentially causative variants in this metabolic association. *CPS1* is highly expressed in liver (S4 Table) and controls the first step in the urea cycle: ammonia is catalyzed to carbamoyl-phosphate, which in turn is the entry substrate of the urea cycle. *CPS1* deficiency can lead to high ammonia levels in the body (Hyperammonemia, OMIM #237300) (S4 Fig). The association of the *CPS1* variants with glycine can be explained by the conversion of excess ammonia to glycine via the glycine cleavage system [78, 79] and is thus biologically meaningful. The association between common variants in *CPS1* and glycine might therefore be driven by mild forms of genetically induced Hyperammonemia. In this study, we could establish a link between genetic factors and a potential urinary marker for this condition.

*SLC5A11* is the only locus where we observe an association with exactly the same metabolite in blood and urine but with reversed effects: while *myo*-inositol concentrations in urine increase per effect allele copy of the lead SNP, they decrease in serum (Table 3) [21]. The *Solute Carrier Family 5 (Sodium/Inositol Cotransporter)*, *Member 11* (*SLC5A11*) is a co-transporter of *myo*-inositol with sodium [80]. *SLC5A11* was postulated to play a role in the regulation of serum *myo*-inositol concentrations [81], which was recently confirmed by an mGWAS in blood [21]. On the one hand, the influence of *SLC5A11* on *myo*-inositol concentrations has been linked to apical transport and absorption in intestine [82]. On the other hand, *SLC5A11* may be implicated in the re-absorption of *myo*-inositol in the proximal tubule of the kidney [83]. The opposite direction of the genetic influence in blood and urine as observed in our study suggests that *SLC5A11* is actively involved in the re-absorption of *myo*-inositol. This assumption is further supported by the strong increase of the association strength when testing the ratio between urinary and serum *myo*-inositol. This could indicate that the reduced levels in blood are indeed caused by a reduced re-absorption rate in subjects that are homozygous regarding the effect allele.

As these examples demonstrate, mGWAS in urine extend our understanding of genetically influenced biochemical processes and can facilitate the knowledge transfer from blood to urine and vice versa. Currently, this transfer is limited by the comparatively low number of GIMs in urine (26) versus blood (>150). Further increasing the sample sizes of mGWAS in urine and the application of more sensitive MS-based metabolomics platforms as already used for blood mGWAS could compensate this bias.

## Methods

### Study samples

For this study, we used data from SHIP (Study of Health in Pomerania) and, for replication, from the KORA (Kooperative Gesundheitsforschung in der Region Augsburg) study. Both studies have been described extensively in the study design papers [84–86] and in our previous publications [4, 6, 10, 21].

SHIP is a longitudinal population study conducted in West-Pomerania, located in the northeastern part of Germany. 4,308 inhabitants in that region participated in the first phase “SHIP-0”. For the GWAS presented here, metabolically characterized urine samples and genotype data were jointly available for 3,861 study participants (1,960 female and 1,901 male, aged 20 to 81 years). KORA is a population study conducted in the municipal region of Augsburg in southern Germany. The KORA F4 cohort comprises 3,080 subjects. For the study presented here, both genotype and urine samples from 1,691 participants (865 female and 826 male, age 32 to 77) were available. In both studies, all participants have given written informed consent and the local ethics committees (SHIP: ethics committee of the University of Greifswald; KORA: ethics committee of the Bavarian Chamber of Physicians, Munich) have approved the studies.

### Metabolomics data acquisition and processing

#### NMR measurements

In SHIP-0, non-fasting, spontaneous urine samples were collected from the study participants. In contrast, KORA F4 study participants were overnight-fasting prior to urine sample collection. All urine samples were stored at -80°C until the analysis. In preparation for the recording of NMR spectra, 75μl of phosphate buffer were added to 675μl of urine to set the pH to 7.0 (+/- 0.35). The deuterated buffer contained 0.5 mM sodium trimethylsilylphosphate (TSP) to provide a reference substance for the annotation of the NMR spectra. One-dimensional ^1^H NMR spectra were recorded at the University of Greifswald, Germany using a Bruker DRX-400 spectrometer (Bruker BioSpin GmbH, Rheinstetten, Germany). Spectra were acquired at 300K and a frequency of 400.13 MHz using a standard one-dimensional NOESY-PRESAT pulse sequence with water peak suppression, as previously described in [10].

#### Targeted analysis

The Fourier-transformed and baseline-corrected NMR spectra were manually annotated by spectral pattern matching using the Chenomx Worksuite 7.0 by Chenomx, Inc. (Edmonton, Canada) to deduce absolute urinary metabolite concentrations. The panel of targeted metabolites comprises 60 compounds (including creatinine, which was used for normalization). In the discovery mGWAS, we used both metabolite concentrations (59) and pairwise ratios thereof (59 × 58/2 = 1,711) from the SHIP-0 data as phenotypic traits. Prior to analysis, individual metabolite concentrations were normalized to the annotated creatinine levels, and both concentrations and ratios were log_10_ transformed. Individual data points more than three times the standard deviation away from the mean were removed to avoid spurious associations. Finally, we considered only metabolic traits with at least 300 valid data points for the GWAS. In total, the final SHIP-0 data set comprises 1,518 targeted metabolic traits (55 metabolites and 1,463 ratios) that were tested for genetic associations.

Likewise, we processed the NMR spectra originating from the KORA F4 data set that was used for replication. Due to the smaller data set size, we lowered the burden for non-missing values to 100. The replication data set contains 53 metabolites and 1,359 pairwise ratios. It covers all metabolic traits that significantly associated in the discovery GWAS.

#### Non-targeted analysis

The same Fourier-transformed and baseline-corrected NMR spectra that were used for the targeted analysis were binned at a segment width of 0.0005 ppm. All signal intensities were normalized to the TSP reference peak. We used the FOCUS software (http://www.urr.cat/FOCUS) [13] for the subsequent processing of the NMR spectra. We excluded the spectral regions between δ = 4.6 and 5.0 ppm (water peak) and the regions below δ = 0.0 and above δ = 10.0 ppm, which do not contain any signals relevant for a metabolomics analysis. We performed the spectral alignment and feature extraction using the default parameters of FOCUS, resulting in a total of 166 NMR peaks in SHIP-0 and 217 peaks in KORA F4. As for the targeted data set, we computed pairwise ratios of the NMR peaks (SHIP-0: 13,695; KORA F4: 23,436). To compensate for dilution effects, we additionally normalized the signal intensities of individual NMR peaks to the annotated creatinine concentrations prior to analysis. All non-targeted traits (i.e., NMR peaks and peak ratios) were log_10_ transformed. In comparison to the targeted data set, we chose a less stringent removal of extreme values. Here, individual data points more than four standard deviations away from the mean were removed.

### Genotype data

Both SHIP and KORA samples were genotyped using Affymetrix Human SNP Array 6.0 gene chips. SNPs were called using the Birdseed2 algorithm. In both data sets, the total genotyping rate was above 99%. 909,508 SNPs were genotyped in the SHIP-0 cohort, and 906,716 SNPs in the KORA F4 cohort. We excluded SNPs that violated the Hardy-Weinberg equilibrium (*P*_HWE_ < 1.0×10^−6^, 8,623 in SHIP and 32,033 in KORA), or had a genotyping rate below 95% (57,160 in SHIP and 84,351 in KORA), or displayed minor allele frequencies (MAF) below 5% (227,967 in SHIP and 224,723 in KORA). After the exclusion, 620,456 autosomal SNPs remained in the SHIP-0 data set and 593,830 autosomal SNPs in the KORA F4 data set. Both SHIP and KORA genotypes were imputed in a two-stage process (pre-phasing followed by imputation). According to data from the 1000 genomes project (phase 1, March 2012 release [50]), we used SHAPEIT (v1.416) [87] for phasing in KORA F4 and IMPUTE (v2.2.2) [88] for imputation in KORA F4 and both phasing and imputation in SHIP-0. For the association analyses, we considered only imputed variants with a MAF ≥ 5%, *P*_HWE_ ≥ 1.0×10^−6^, and an imputation quality score (IMPUTE info-score) ≥ 0.8.

### Statistical analysis

#### Genome-wide association study

In both the SHIP-0 and KORA F4 cohorts, we used PLINK (v1.07) [89] to compute age- and sex corrected linear regression models under the assumption of an additive genetic model. For the discovery study based on the SHIP-0 data set, we carried out association tests for each genotyped autosomal SNP (filtered for the criteria described above) and all 15,379 targeted and non-targeted metabolic traits. In addition, we tested all imputed, quality-filtered SNPs within a physical distance of 1 Mb to each genotyped SNP that displayed an association signal of *P* < 5×10^−8^. This two-stage approach of testing imputed variants in selected candidate regions results in a drastically lowered computational burden when compared to association studies with imputed variants over the whole genomic range (0.9M vs. 15.9M tested SNPs). We considered associations to be genome-wide significant if the resulting *P* value was below the Bonferroni-adjusted significance threshold of 5×10^−8^/15,379 = 3.25×10^−12^. In case of associations with ratios, we imposed an additional significance criterion on the *P*-gain as suggested by Petersen *et al*. [53]. *P*-gain describes the observed increase in strength of association when compared to the association with the individual metabolic traits from which the ratio was computed (min(*P*(M_1_)/*P*(M_1_/M_2_), *P*(M_2_)/*P*(M_1_/M_2_))). A conservative Bonferroni-type lower limit on the *P*-gain at a significance level of 5% is given by ten times the number of tested ratio pairs. Thus, associations with ratios were considered to be significant if the *P*-gain exceeded 15,180 in case of targeted metabolic ratios and 138,610 in case of non-targeted ratios. The analysis of the PLINK output files was performed using in-house R (v3.0.2) code (available at http://www.gwas.eu).

#### Replication of association results

To replicate the significant associations discovered in the SHIP-0 data set, we used data from the independent KORA F4 cohort. For each genetic locus, we attempted to replicate the association of the SNP/metabolic trait pair that displayed the lowest *P-*value (S1 and S2 Tables). If an association did not replicate, we checked whether the tested SNP was significantly associated with other metabolic traits in SHIP-0. In that case, we tried to replicate these associations, beginning with the one that displayed the strongest association signal. To replicate associations with non-targeted traits, we selected the NMR feature with the minimum difference in chemical shift. In total, we attempted the replication of 38 SNP/metabolic trait associations. Thus, we considered associations with *P* < 0.05/38 = 1.32×10^−3^ to be successfully replicated in the KORA F4 cohort.

### Genetic variant annotation and evidence-based locus to gene mapping

Annotation data for genetic variants as well as linkage disequilibrium (LD) data from the 1000 genomes project (phase 1 version 3, EUR panel [50]) were retrieved from SNiPA v1 (http://www.snipa.org) [51]. Full lists of association signals from the serum-based mGWAS [21] were obtained from the Metabolomics GWAS server (http://www.gwas.eu).

All genotyped and imputed SNPs that displayed genome-wide significant association signals (according to the aforementioned *P*-value and *P*-gain criteria) were assigned to distinct genetic regions (loci), based on a physical distance threshold of 1 Mb. Each of the resulting 23 genome-wide significant loci was then projected to candidate genes using an evidence-based procedure. To this end, we used the “block annotation” feature of SNiPA on the LD-extended (r^2^ ≥ 0.8) list of associated variants at each locus. This feature provides a condensed view of genes that are linked to any of the significantly associated variants or their LD-proxies via genomic proximity, eQTL association, or regulatory elements. Additionally, the block annotation highlights missense and pathogenic variants. Based on these data, we defined the following criteria to identify candidate genes: 1) Genomic proximity: genes that harbor or are in close proximity (<5kb) to any of the variants in the list. 2) eQTL association: genes where altered expression levels have been discovered to associate with any of the variants in the list. 3) Regulatory elements: potentially regulated genes that are associated with a promoter/enhancer/repressor element containing a variant of the list. Further evidence for potential involvement of a gene was assumed if 4) the variant list contains a missense variant for a protein product of this gene and 5) if an intragenic variant in the list is annotated as pathogenic in one of the phenotype databases contained in SNiPA. For each gene, we counted how many of the aforementioned criteria are met. Thus, the maximum evidence count for a candidate gene is five. If evidence-based gene selection was ambiguous, the gene with the most plausible biological function was chosen (S3 Table).

### Metabomatching of non-targeted NMR traits

Metabomatching [12] is an automated annotation method that identifies metabolites likely to underlie an observed genetic association between a SNP and one or more non-targeted metabolic traits (www.unil.ch/cbg). It does so by comparing the association signal between a SNP and all non-targeted traits (pseudo-spectrum or association spectrum) to the ^1^H-NMR spectra of metabolites in a reference set, and assigning a score to each pseudo-spectrum to NMR spectrum match. The metabolites most likely to underlie the genetic association are the ones with the highest scores. We applied metabomatching to each SNP showing a significant association with an NMR feature or feature-ratio, using the 180 metabolites listed in the urine metabolome database [54] with an experimental NMR spectrum as reference set. The urine metabolome database is the subset of metabolites in the Human Metabolome Database (HMDB) [90] present in urine. Where indicated, 2-compound or effect direction-specific metabomatching was applied. Final candidates were manually identified, usually among the few top-ranked matches. Most likely candidates are used in the text; potential alternatives are listed, along with the metabomatching details, in S1 Fig.

## Supporting Information

S1 Fig Metabomatching on SNPs associated with non-targeted metabolic traits. The subfigures (a)-(s) show the most likely candidate metabolite as suggested by metabomatching for each locus in Table 2 (except *HIBCH*, for which no match was found). Top panels contain the pseudo-spectrum of the SNP with the strongest association within its locus, bottom panels show the NMR spectrum of the most likely candidate metabolite(s). For pseudo-spectra, only peaks with-log(*P*) > 1.3 (*P* < 0.05) are displayed, and peak color indicates effect direction, with blue for *β* > 0 and orange for *β* < 0. The most likely candidate metabolite is not necessarily the metabolite of top rank: instead, it is manually selected among top-ranked metabolites. For some loci, the pseudo-spectrum indicates the involvement of more than one compound. For *CPS1* (b) and *HPD* (o), 2-compound metabomatching, which ranks compound pairs rather than individual compounds, produces viable candidates. For *SLC6A19* (g), *NAT2* (k), and *PNMT* (r), the pseudo-spectra allow too many viable compound pairs even for 2-compound metabomatching, so these matches were manually selected.
(PDF)

S2 Fig Regional association plots, box plots, and quantile-quantile plots for the loci with genome-wide significant associations. Top: The regional association plot displays SNPs in a locus and the strength of association to the top-associated metabolic trait (Tables 1 and 2). The plot window (500 kb) is centered on the SNP that displays the strongest association signal and, in case of ratios, meets the *P*-gain criterion (“lead SNP”, indicated in blue). The variants are colored according to the degree of linkage disequilibrium (LD) to the lead SNP. The plot symbols and border colors indicate functional annotations (e.g. effects on transcripts) as well as results from other association studies. Plots were created with the SNiPA web service (available at www.snipa.org) using data from the 1000 genomes project (phase 1 version 3, European super-population) and Ensembl 75. Bottom left: Top associated metabolic trait in SHIP-0 grouped by genotype of the lead SNP (in order major allele homozygotes, heterozygotes, and minor allele homozygotes). Bottom right: Quantile-quantile plots displaying the observed vs. the expected distribution of *P*-values for associations to the top-associated metabolic trait.
(PDF)

S3 Fig Polymorphisms in *XYLB* might induce a switch to an alternative xylulose pathway via *phosphofructokinase* (*PFK*). The mGWAS identified significant associations of SNP rs3132440 (intronic region of *XYLB*) and rs17118 (non-synonymous variant in *XYLB*) with glycolate. The urinary concentration increases per copy of the effect allele (indicated by the yellow arrow). Glycolate is a downstream product of the PFK-mediated D-xylulose pathway. Thus, the associated variants could decrease the enzymatic activity of *XYLB*’s gene product (*xylulokinase*; *XK*), which would lead to an increased D-xylulose metabolism via *PFK*. Figure adapted from [70, 91].
(PDF)

S4 Fig Potential relationship between CPS1 deficiency and elevated glycine levels. A deficiency of *Carbamoyl-Phosphate Synthetase 1* (*CPS1*) can lead to high ammonia levels in blood (top). We detected significant associations of variants in *CPS1* and elevated urinary glycine levels (indicated by the yellow arrow). We hypothesize that the excess ammonia is converted to glycine via the Glycine Cleavage System (bottom).
(PDF)

S1 Table Replication results for the 15 lead SNPs and their associations to targeted metabolic traits in the KORA F4 cohort. In case of association to ratios, the metabolite that shows the stronger association signal is listed in the numerator. Tests: number of conducted association tests on the SNP. The significance level was adjusted for 38 tests (*P* < 0.05/38 = 1.32×10^−3^), which is the sum of replication attempts using targeted traits and non-targeted traits (S2 Table). In the targeted data set, the strongest identified association between rs7247977 (*SLC7A9*) and the lysine/valine ratio could not be replicated. Instead, the second-strongest, still significant association of this SNP with the concentrations of valine was successfully replicated.
(DOCX)

S2 Table Replication results for the 20 lead SNPs and their associations to non-targeted metabolic traits in the KORA F4 cohort. Non-targeted traits are reported as chemical shifts (i.e., the position in the NMR spectrum). To replicate the non-targeted association results, we selected the NMR peaks or ratios thereof that were closest to the NMR features used in the SHIP-0 data set. For five loci (*ETNPPL*, *SLC6A19*, *DMGDH*, *ABO*, and *PNMT*), we were unable to replicate the top associations in the KORA F4 data set. These associations are marked with an asterisk (*).
(DOCX)

S3 Table Evidence-based locus-to-gene assignment. (XLSX)

S4 Table Gene expression rates from the Illumina Body Map Project 2.0. Expression rate (given as reads per kilobase of transcript per million mapped reads; RPKM) for the most likely effector gene per locus as determined in 16 different tissues.
(DOCX)
