# Supplementary material for: Genome-Wide Association Study with Targeted and Non-targeted NMR Metabolomics Identifies 15 Novel Loci of Urinary Human Metabolic Individuality
Source: PLoS Genet. 2015 Sep 9;11(9):e1005487. doi: 10.1371/journal.pgen.1005487 (PMC4564198; doi:10.1371/journal.pgen.1005487)
Supplement: S1 Fig — The subfigures (a)-(s) show the most likely candidate metabolite as suggested by metabomatching for each locus in Table 2 (except HIBCH, for which no match was found). Top panels contain the pseudo-spectrum of the SNP with the strongest association within its locus, bottom panels show the NMR spectrum of the most likely candidate metabolite(s). For pseudo-spectra, only peaks with-log(P) > 1.3 (P < 0.05) are displayed, and peak color indicates effect direction, with blue for β > 0 and orange for β < 0. The most likely candidate metabolite is not necessarily the metabolite of top rank: instead, it is manually selected among top-ranked metabolites. For some loci, the pseudo-spectrum indicates the involvement of more than one compound. For CPS1 (b) and HPD (o), 2-compound metabomatching, which ranks compound pairs rather than individual compounds, produces viable candidates. For SLC6A19 (g), NAT2 (k), and PNMT (r), the pseudo-spectra allow too many viable compound pairs even for 2-compound metabomatching, so these matches were manually selected. (PDF) [file pgen.1005487.s001.pdf]

S1 Fig.: **Metabomatching on SNPs associated with non-targeted metabolic traits.** Each figure shows the most likely candidate metabolite as suggested by metabomatching. Top panels contain the pseudo-spectrum of the SNP with the strongest association within its locus, bottom panels the NMR spectrum of the most likely candidate metabolite(s). For pseudo-spectra, only peaks with $-\log(P) > 1.3$ ($P < 0.05$) are displayed, and peak color indicate effect direction, with blue for $\beta > 0$ and orange for $\beta < 0$. The most likely candidate metabolite is not necessarily the metabolite of top rank: instead, it is manually selected among top-ranked metabolites. For some loci, the pseudo-spectrum indicates the involvement of more than one compound. For *CPS1* (b) and *HPD* (o), 2-compound metabomatching, which ranks compound pairs rather than individual compounds, produces viables candidates. For *SLC6A19* (g), *NAT2* (k), and *PNMT* (r), the pseudo-spectra allow too many viable compound pairs even for 2-compound metabomatching, so that matches were manually selected.

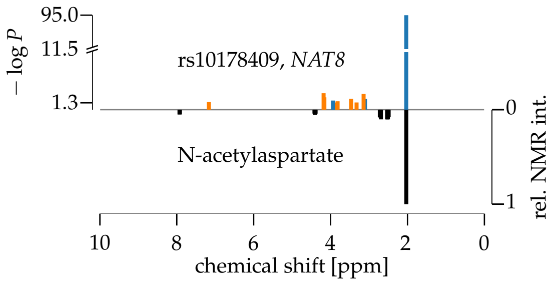

(a) Although it has unmatched secondary peaks, N-acetylaspartate (rank 4) is the most likely candidate, followed closely by isovalerate (rank 2).

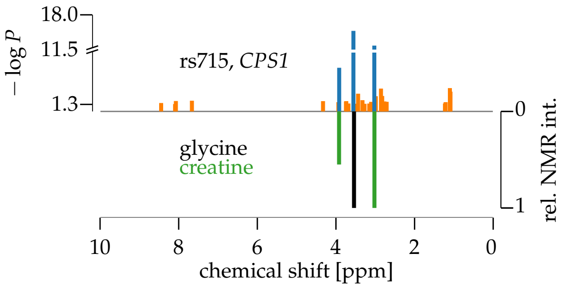

(b) No viable candidate matches the three leading peaks, so that 2-compound metabomatching was applied. In addition, leading peaks of opposing effect direction were zeroed out before metabomatching. The compound pair glycine (rank 10) and creatine (rank 2) perfectly matches the pseudo-spectrum (pair rank 1).

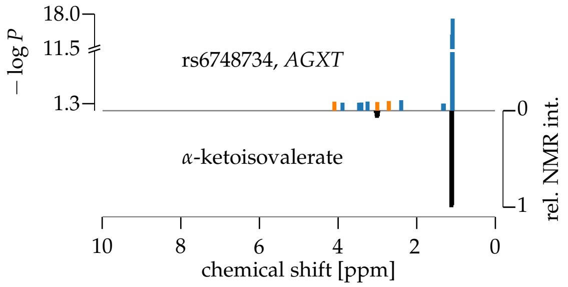

(c) α-ketoisovalerate (rank 3) is the most likely candidate, followed closely by 2-ketobutyrate (rank 1) and methylsuccinate (rank 5).

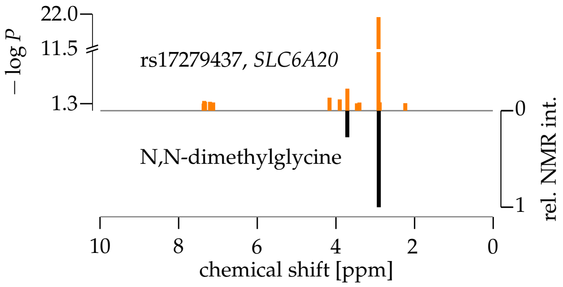

(d) Top-ranked N,N-dimethylglycine is a strong 2-peak match.

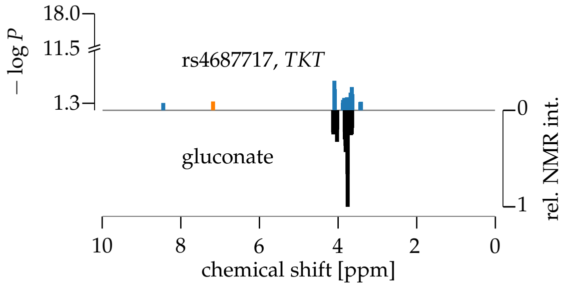

(e) The main pseudo-spectrum peaks lie in a region matched by many sugars, alcohols, and related compounds, making identification of a candidate difficult. Gluconate (rank 2) is the most likely candidiate, followed closely by sucrose (rank 1) and D-galactose (rank 3).

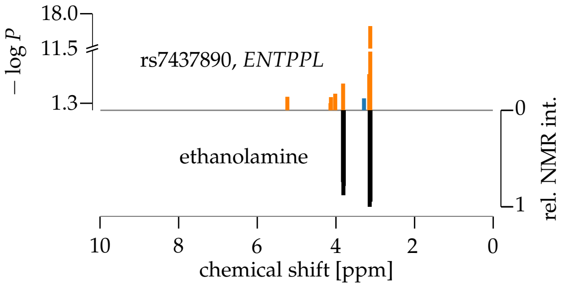

(f) Top-ranked ethanolamine is a strong 3-peak match (including 3.152 and 3.126, difficult to distinguish in this figure).

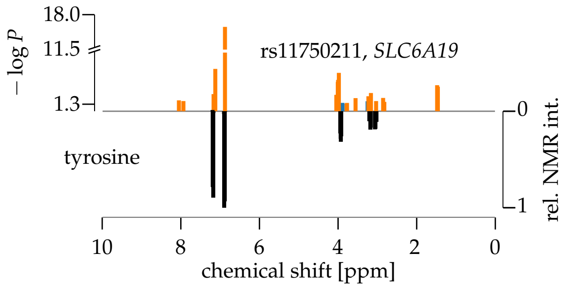

(g) Tyrosine (rank 9) is the highest ranked candidate for 6.877. Secondary signal points to histidine and alanine.

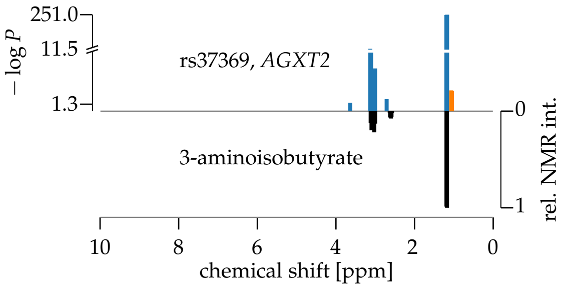

(h) Top-ranked 3-aminoisobutyrate is a strong 3-peak match.

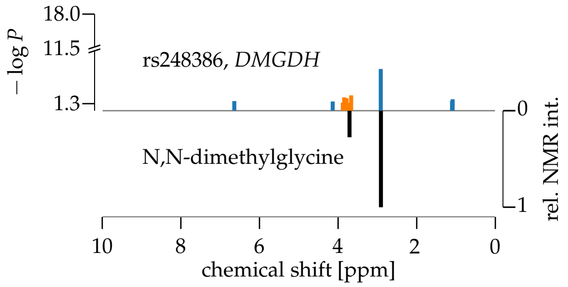

(i) N,N-dimethylglycine (rank 3) is the most likely candidate, followed closely by N-methlyhydantoin (rank 5)

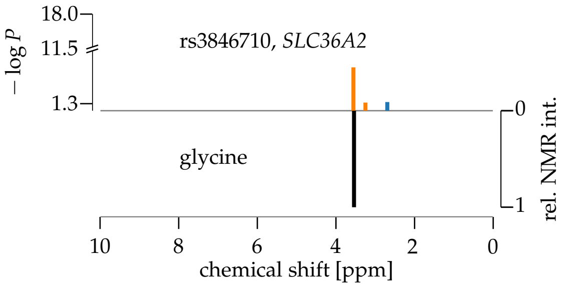

(j) Top-ranked glycine is a clear match.

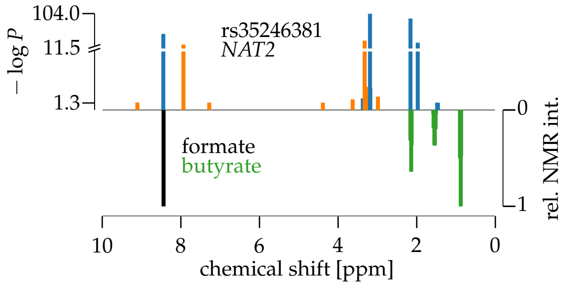

(k) Metabomatching on positive effect size peaks. No compound matches both 2.159, and any of the other strong signals. Individually, 2.159 is best matched by butyrate, followed by sebacate and azelate. Formate is the top-ranked candidate matching 8.447. Acetylcarnitine and N-acetylputrescine are the most likely candidates for the remaining positive effect size peaks. The most likely candidate pair to match the negative effect size peaks are τ-methylhistidine and 1,3-dimethylurate.

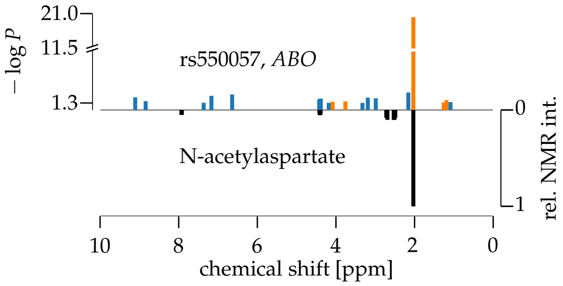

(l) Although it has unmatched secondary peaks, N-acetylaspartate (rank 2) is the most likely candidate, followed by isovalerate (rank 3).

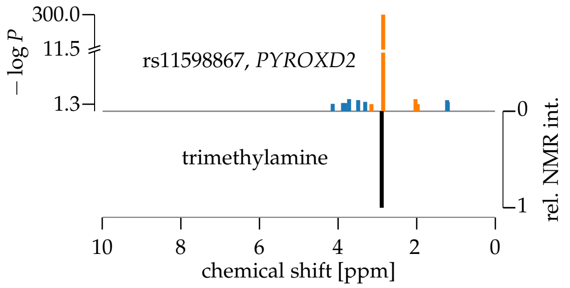

(m) Top-ranked trimethylamine is the most likely candidate, followed closely by methylguanidine (rank 2).

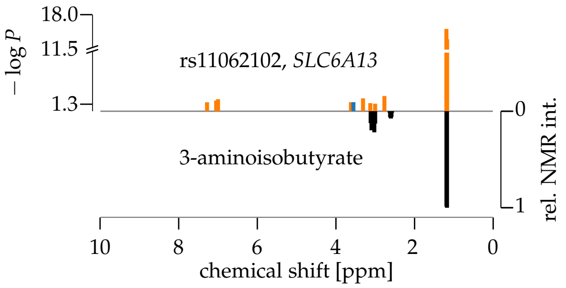

(n) 3-aminoisobutyrate is the most likely candidate, followed closely by isobutyrate (rank 1) and ethanol (rank 2).

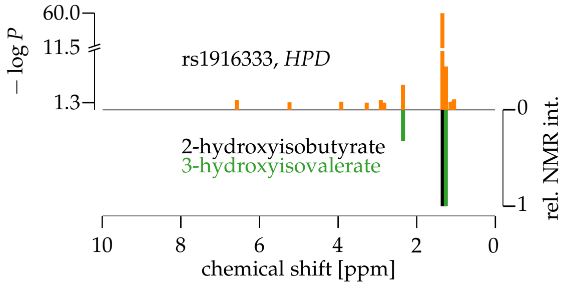

(o) No viable candidate matches the 3 leading peaks, so that 2-compound metabomatching was applied. Top-ranked pair 2-hydroxyisobutyrate and 3-hydroxyisovalerate is the most likely candidate pair.

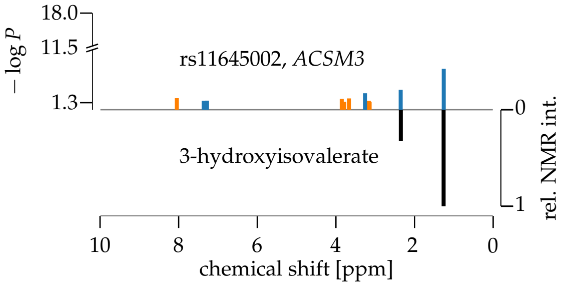

(p) Top-ranked 3-hydroxyisovalerate is a strong 2-peak match.

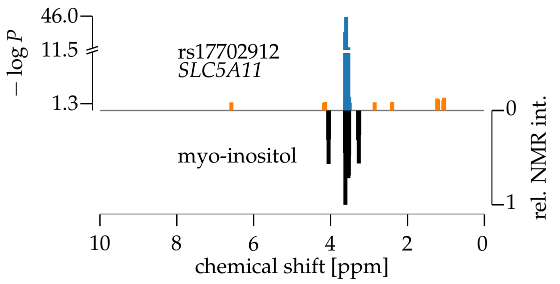

(q) The main pseudo-spectrum peaks are matched by polyols, most likely myo-inositol (rank 1) and glycerol (rank 2).

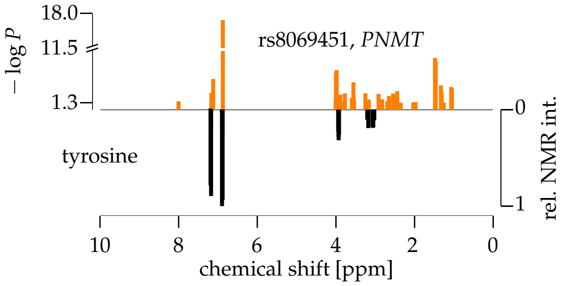

(r) Tyrosine (rank 14) is the highest ranked candidate for 6.877. Secondary signal points to histidine, alanine, and threonine.

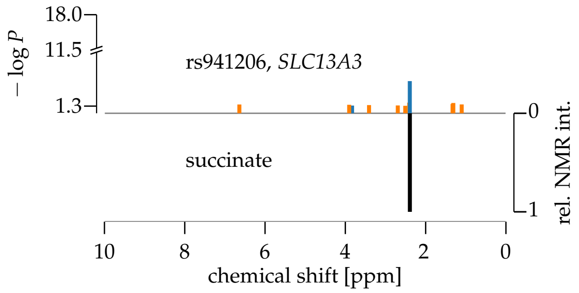

(s) Top-ranked succinate is a clear match.
