# Supplementary material for: Genome-Wide Association Study with Targeted and Non-targeted NMR Metabolomics Identifies 15 Novel Loci of Urinary Human Metabolic Individuality
Source: PLoS Genet. 2015 Sep 9;11(9):e1005487. doi: 10.1371/journal.pgen.1005487 (PMC4564198; doi:10.1371/journal.pgen.1005487)

GWAS with **targeted** metabolic traits - **CPS1** locus

Associations with Glycine/Threonine

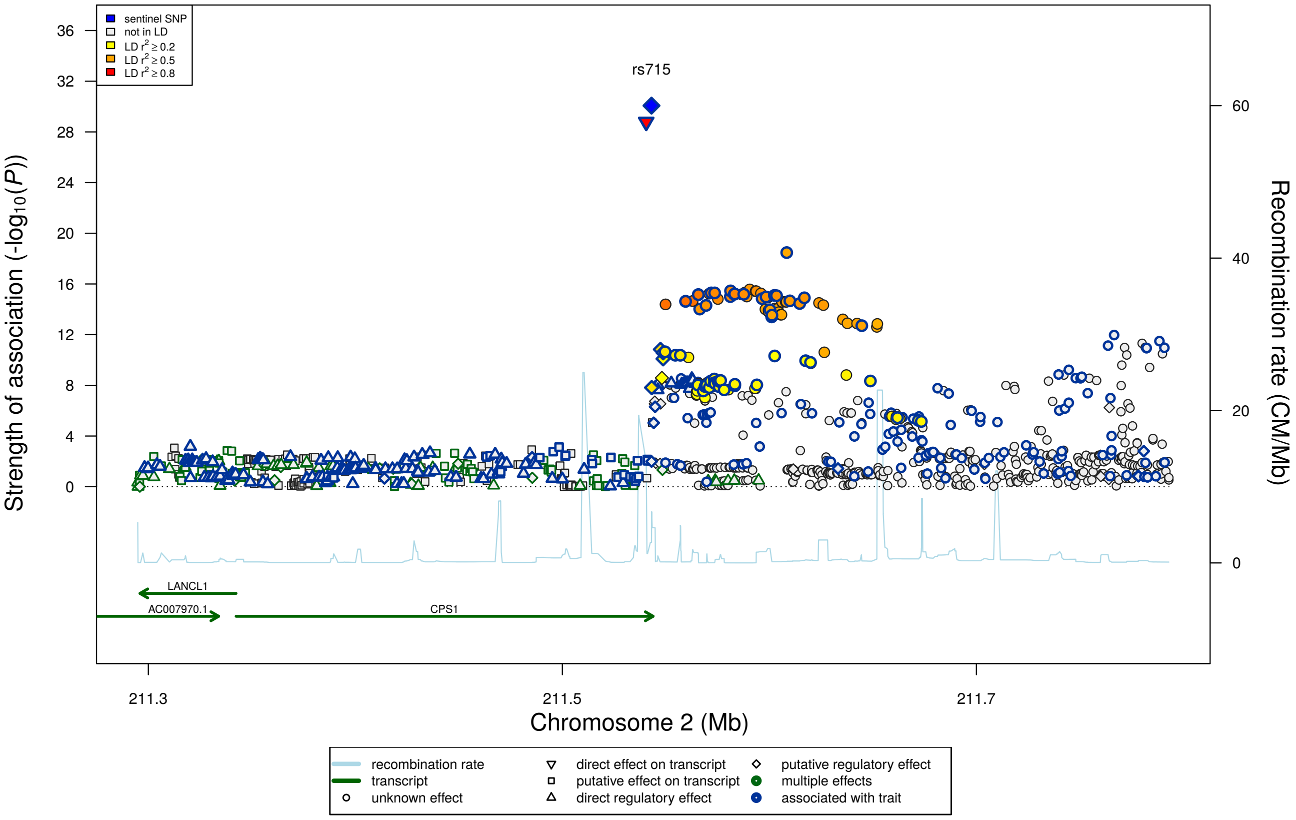

Genotype of rs715

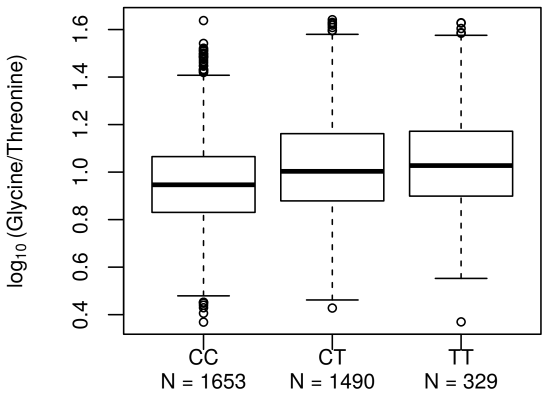

Genome−wide associations with Glycine/Threonine

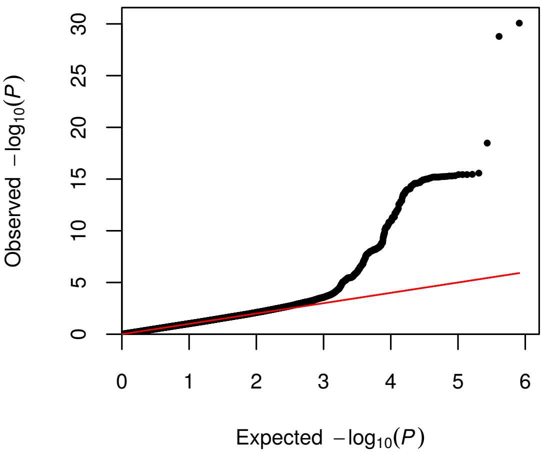

GWAS with **targeted** metabolic traits - **XYLB** locus

Associations with Glycolate

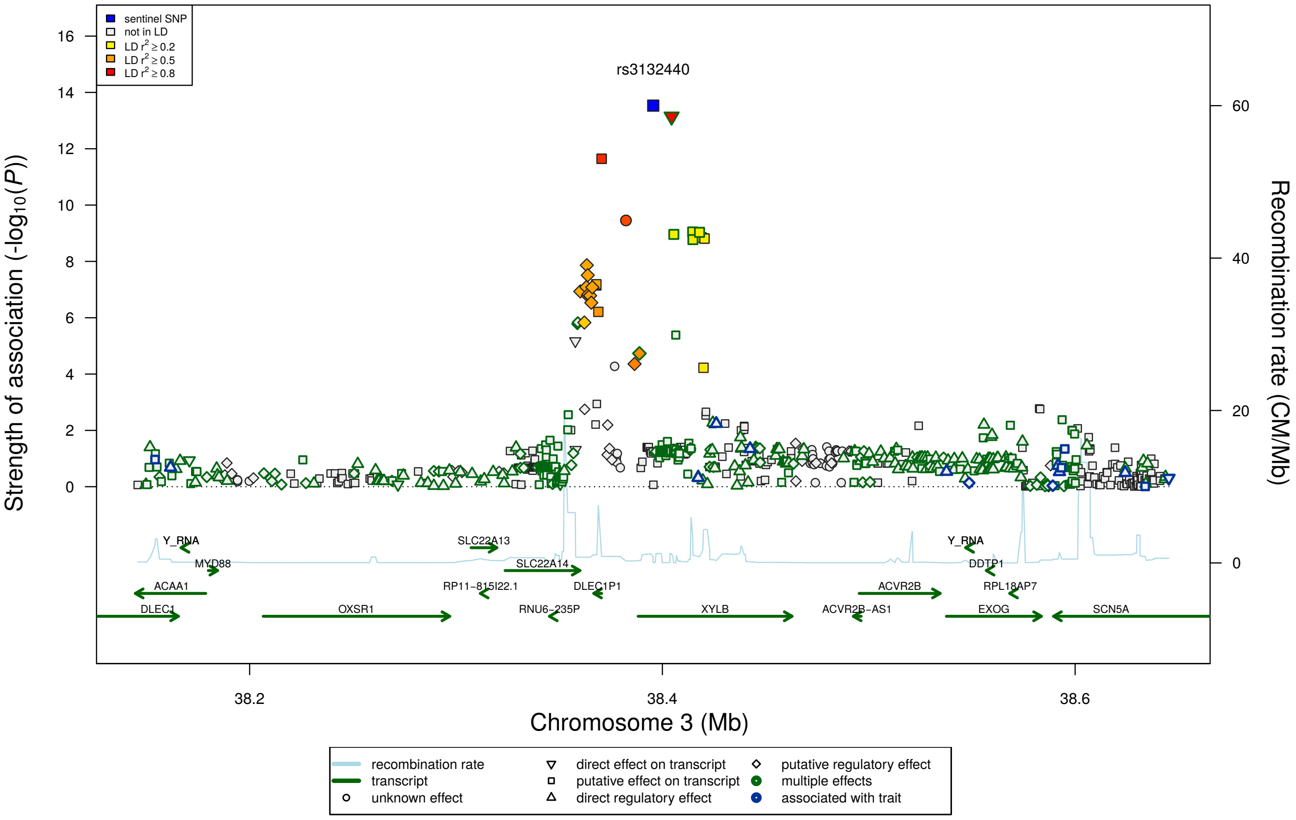

Genotype of rs3132440

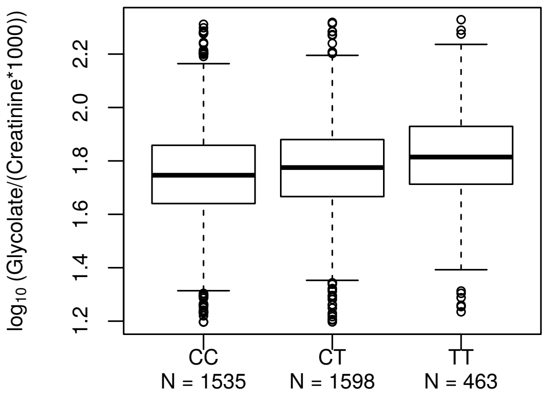

Genome−wide associations with Glycolate

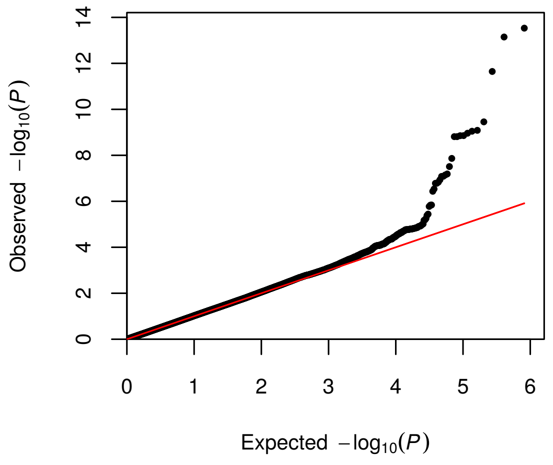

GWAS with **targeted** metabolic traits - **SLC6A20** locus

Associations with N,N-Dimethylglycine/Alanine

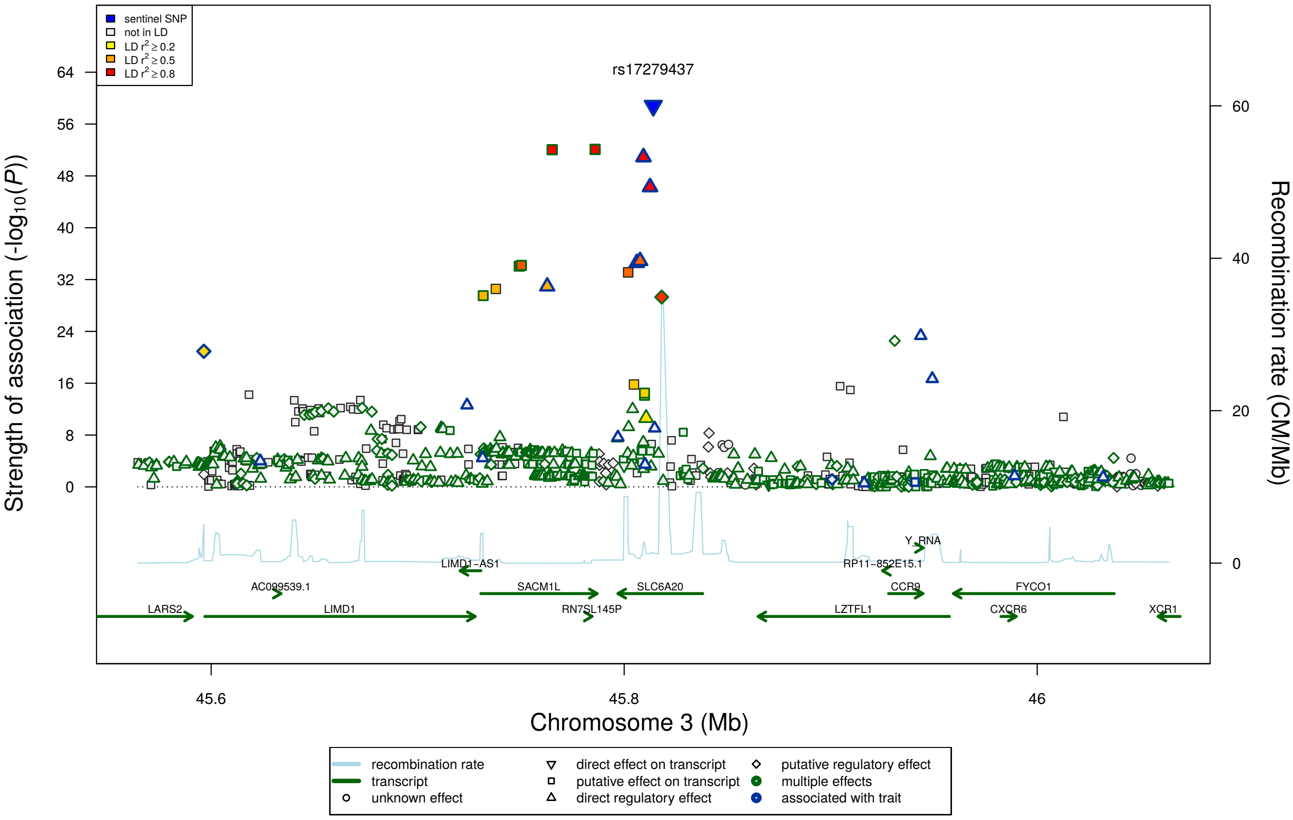

Genotype of rs17279437

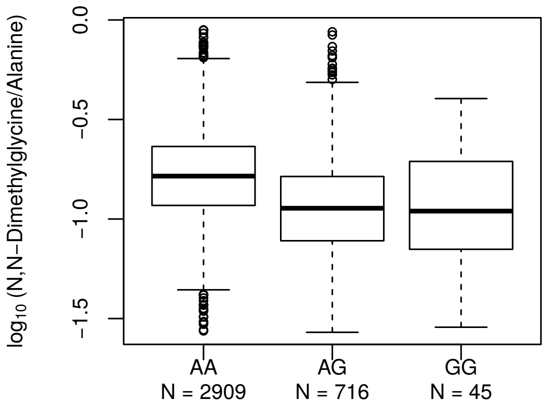

Genome−wide associations with N,N−Dimethylglycine/Alanine

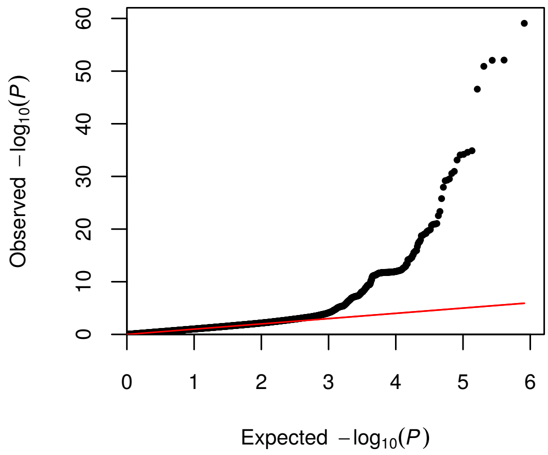

GWAS with **targeted** metabolic traits - **ETNPPL** locus

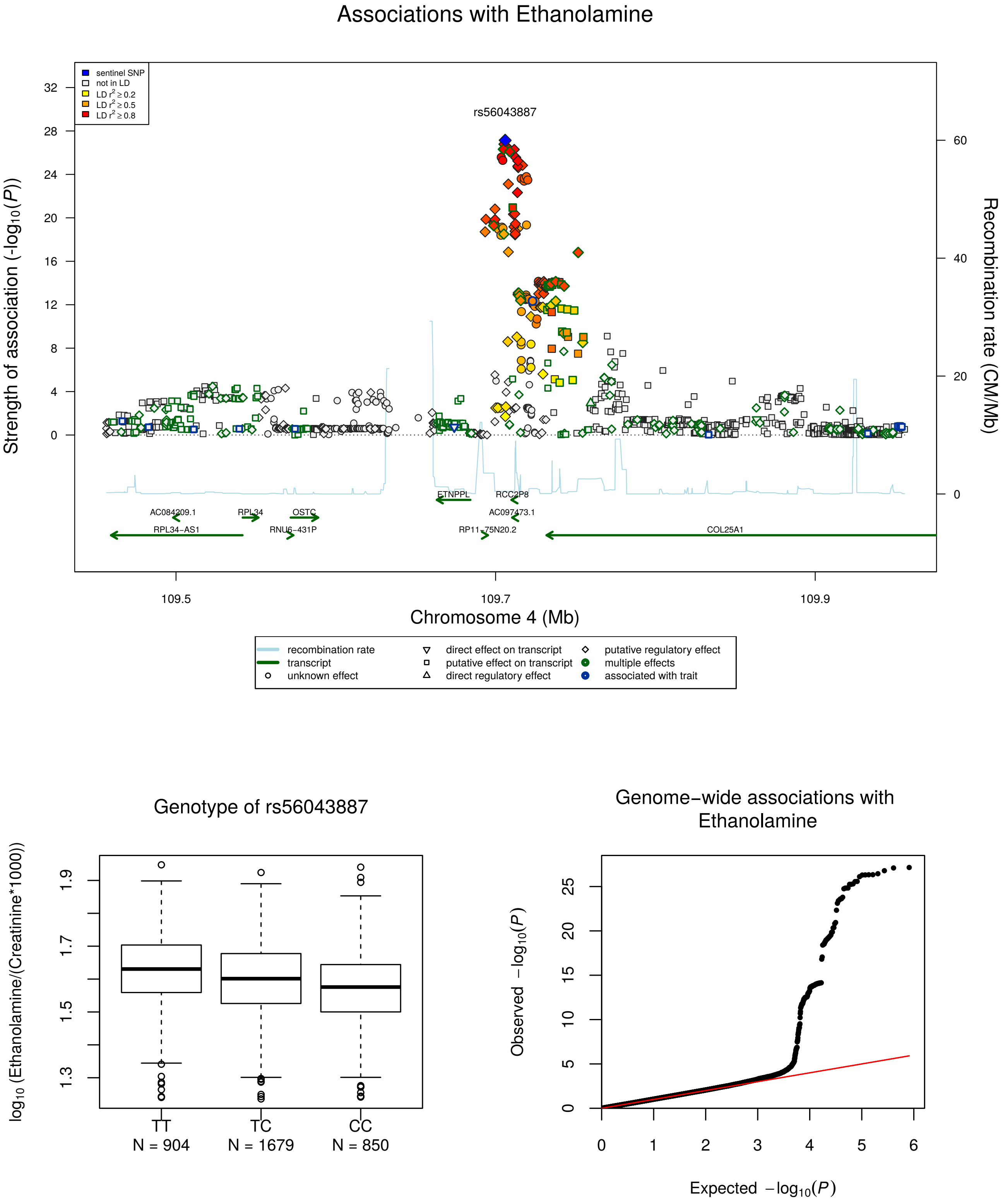

GWAS with **targeted** metabolic traits - **SLC6A19** locus

Associations with Histidine/tau-Methylhistidine

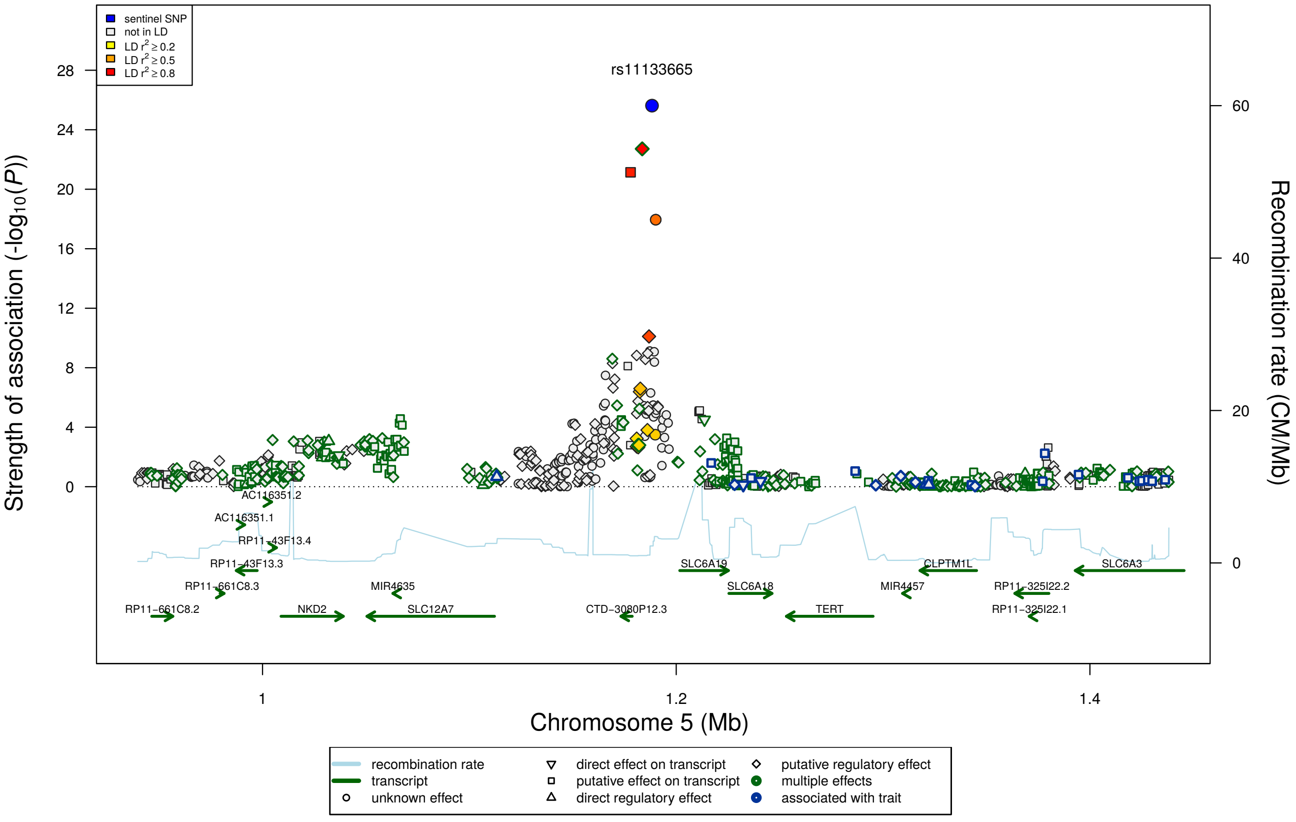

Genotype of rs11133665

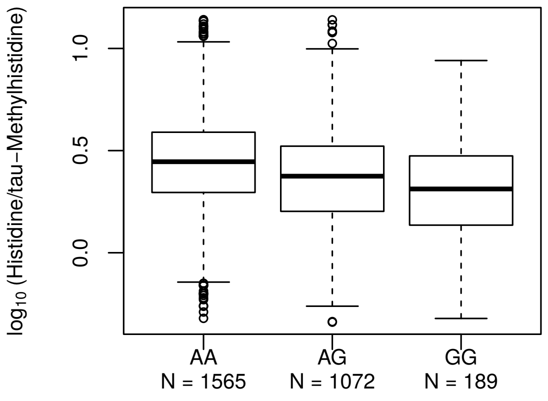

Genome-wide associations with Histidine/tau-Methylhistidine

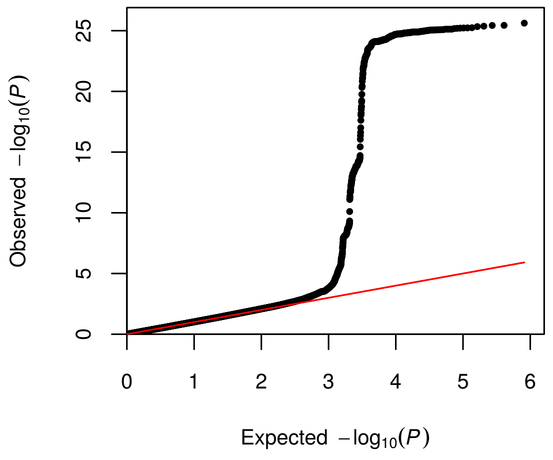

GWAS with **targeted** metabolic traits - **AGXT2** locus

Associations with 3-Aminoisobutyrate

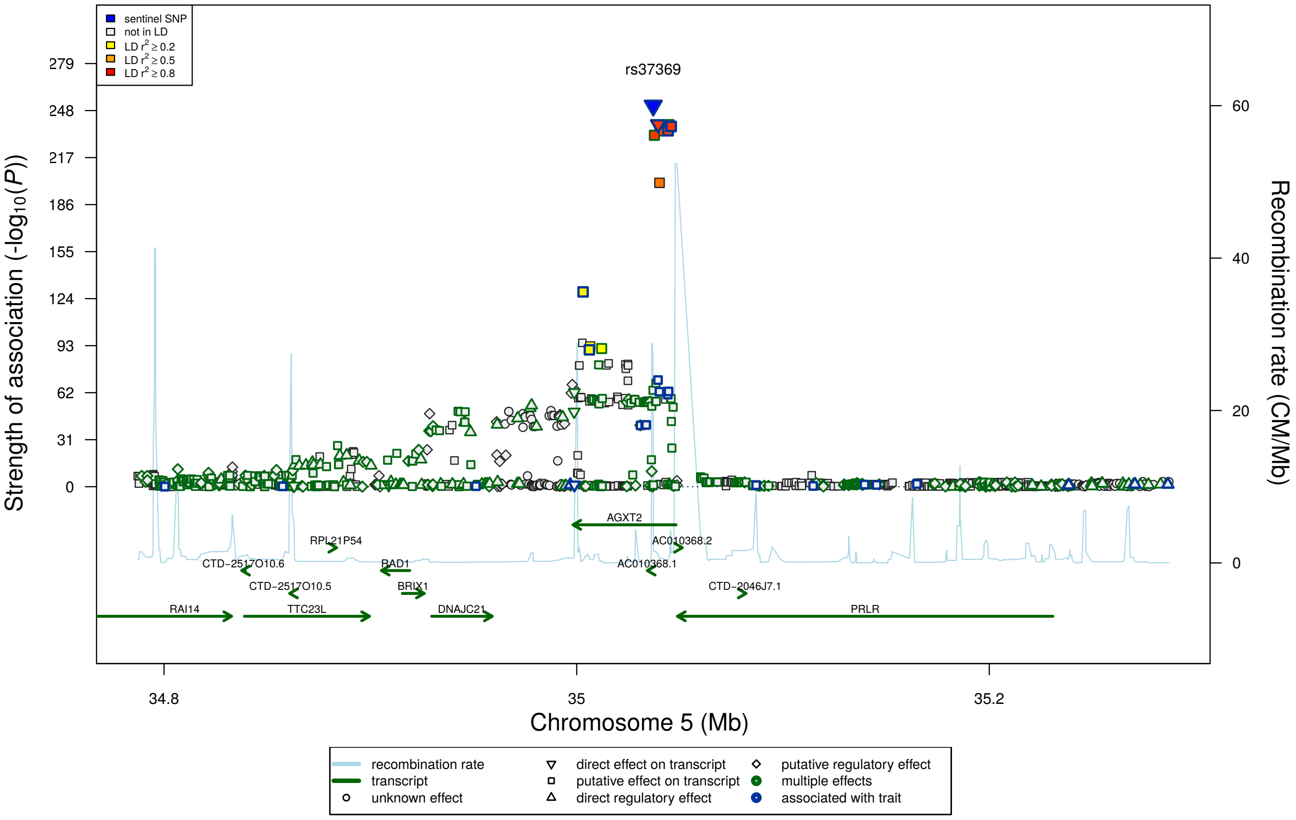

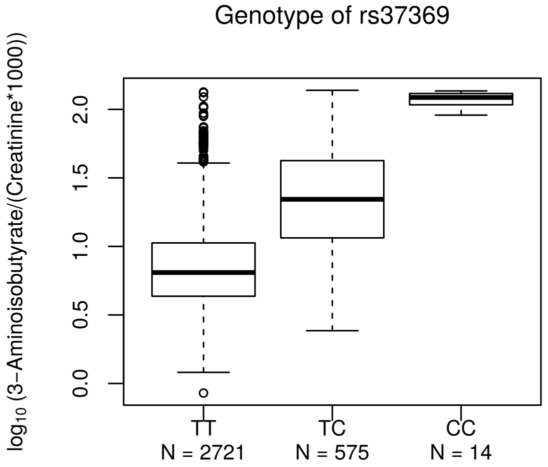

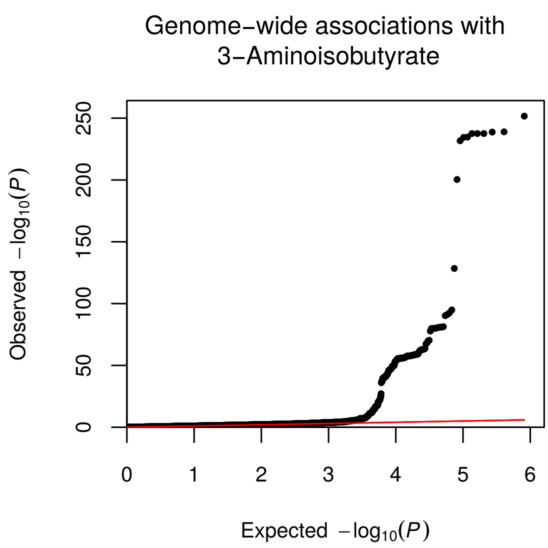

GWAS with **targeted** metabolic traits - **DMGDH** locus

Associations with N,N-Dimethylglycine/Betaine

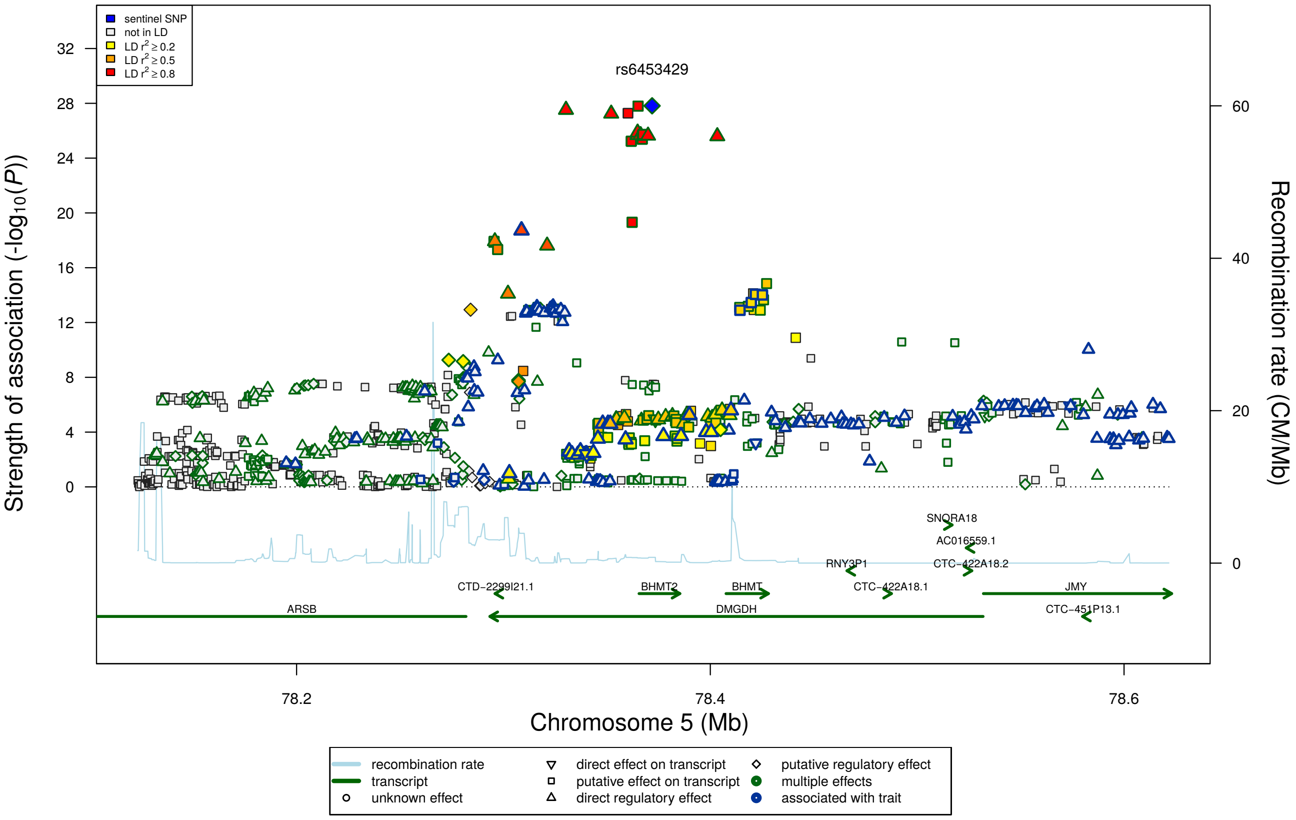

Genotype of rs6453429

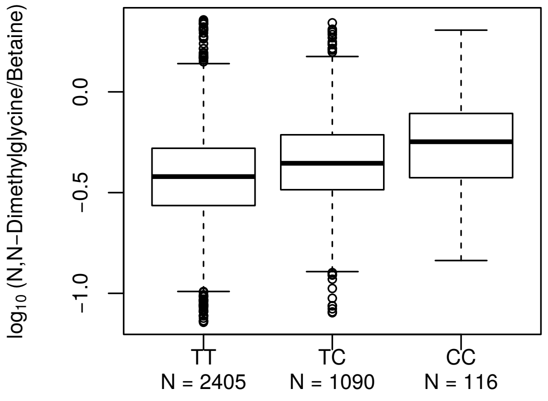

Genome−wide associations with N,N−Dimethylglycine/Betaine

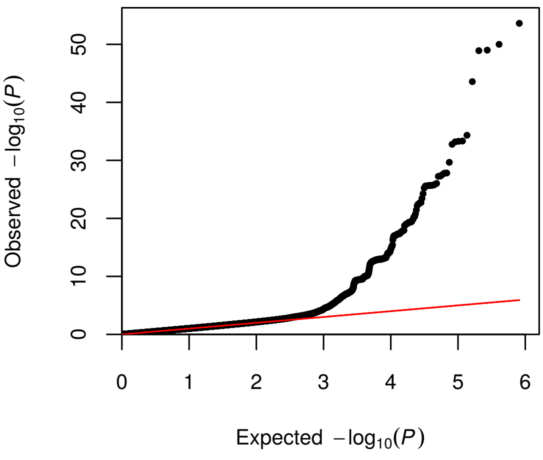

GWAS with **targeted** metabolic traits - **SLC36A2** locus

Associations with Glycine/Citrate

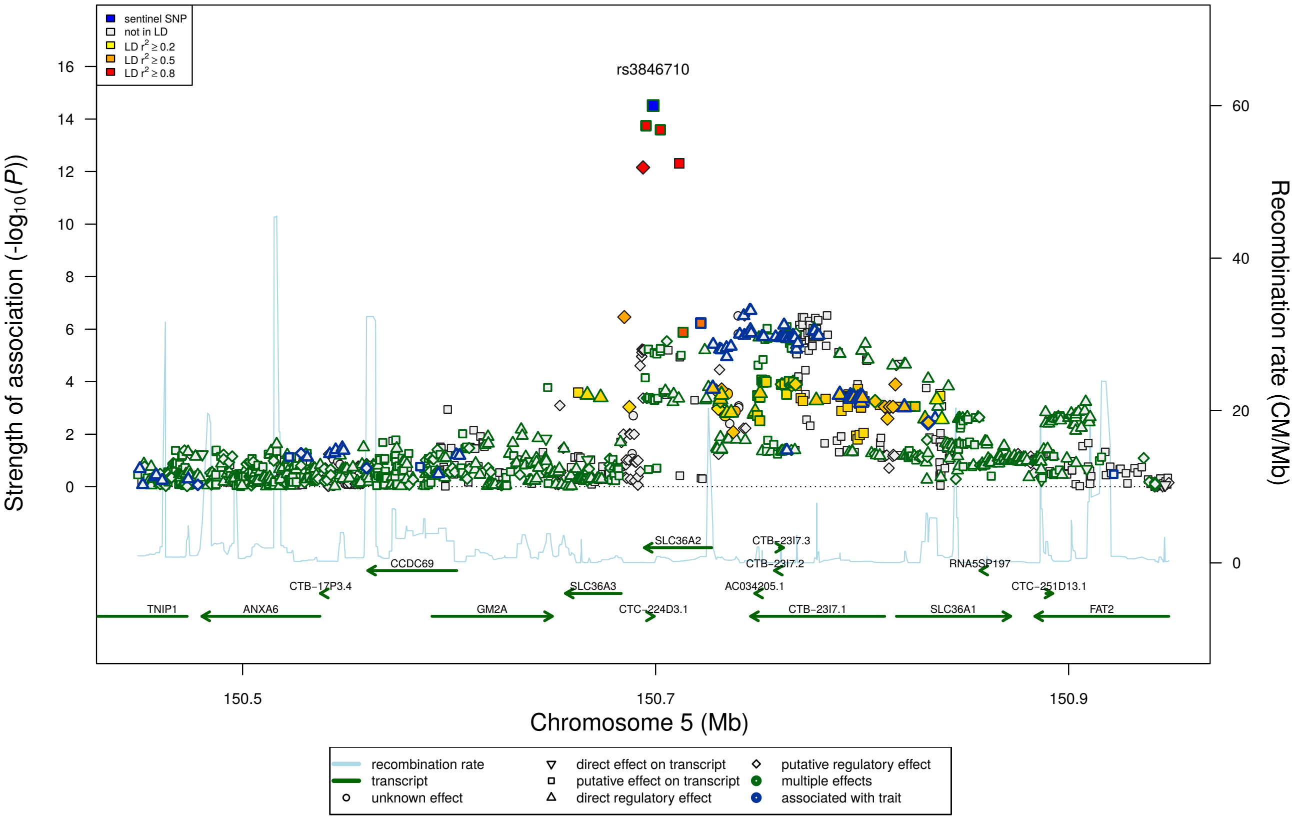

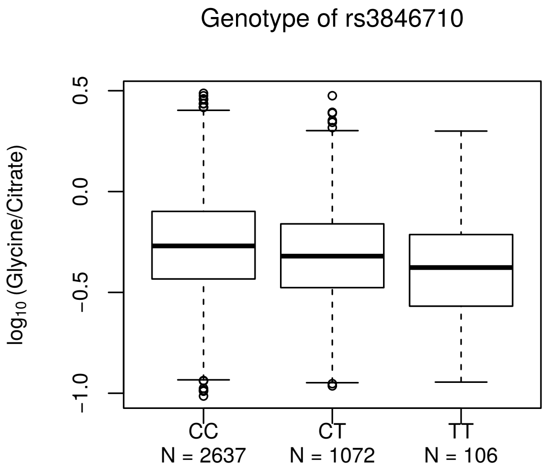

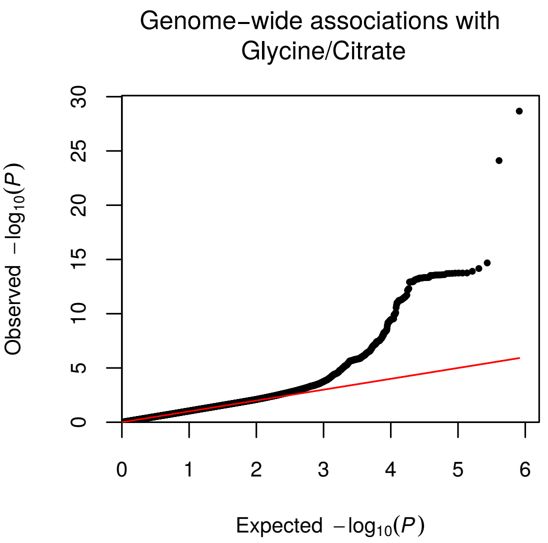

GWAS with **targeted** metabolic traits - **NAT2** locus

Associations with Formate/Acetate

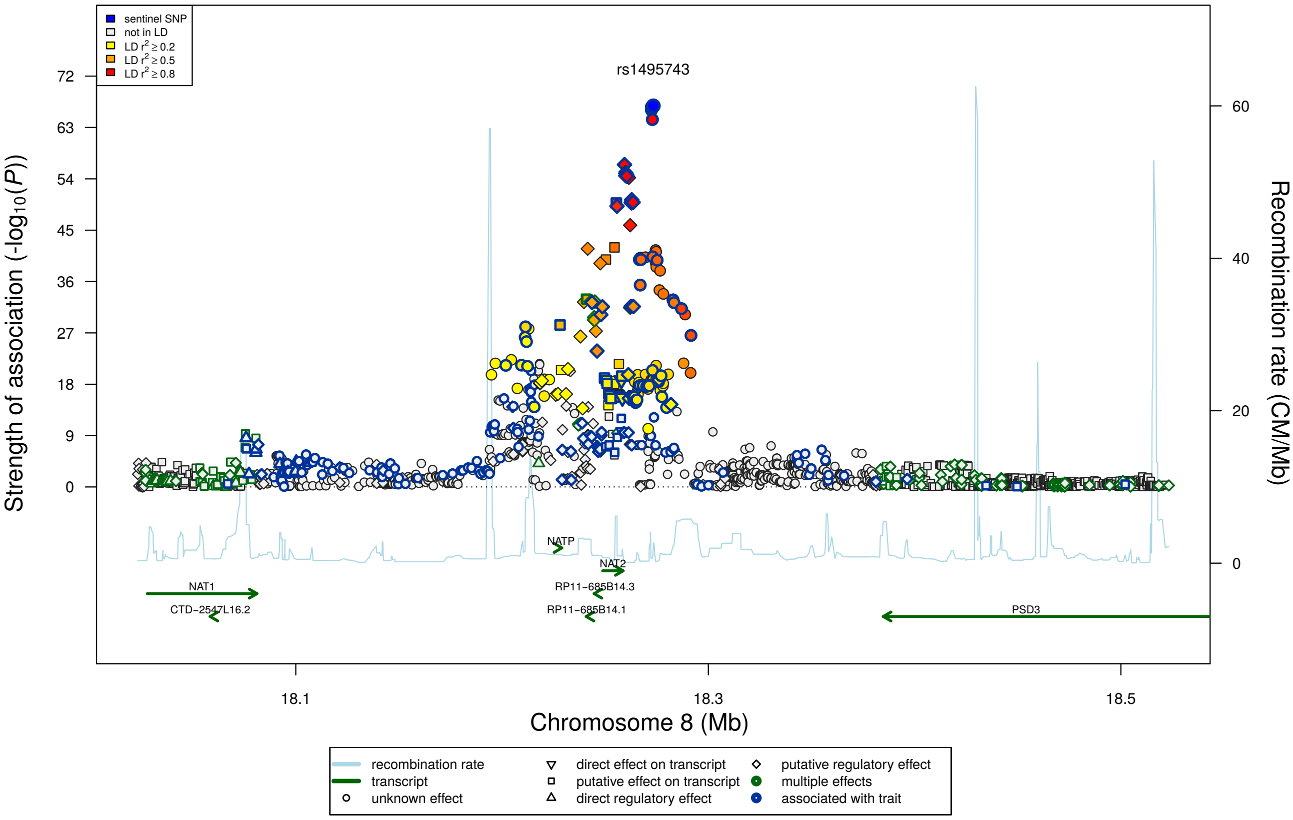

Genotype of rs1495743

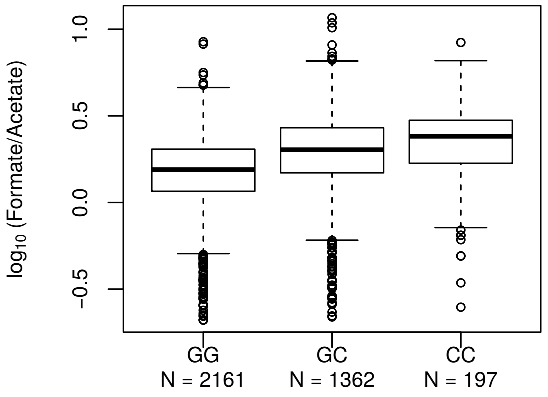

Genome−wide associations with Formate/Acetate

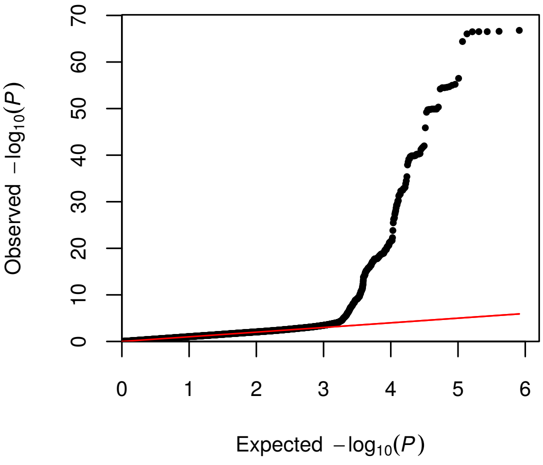

GWAS with **targeted** metabolic traits - **GLDC** locus

Associations with Glycine/Alanine

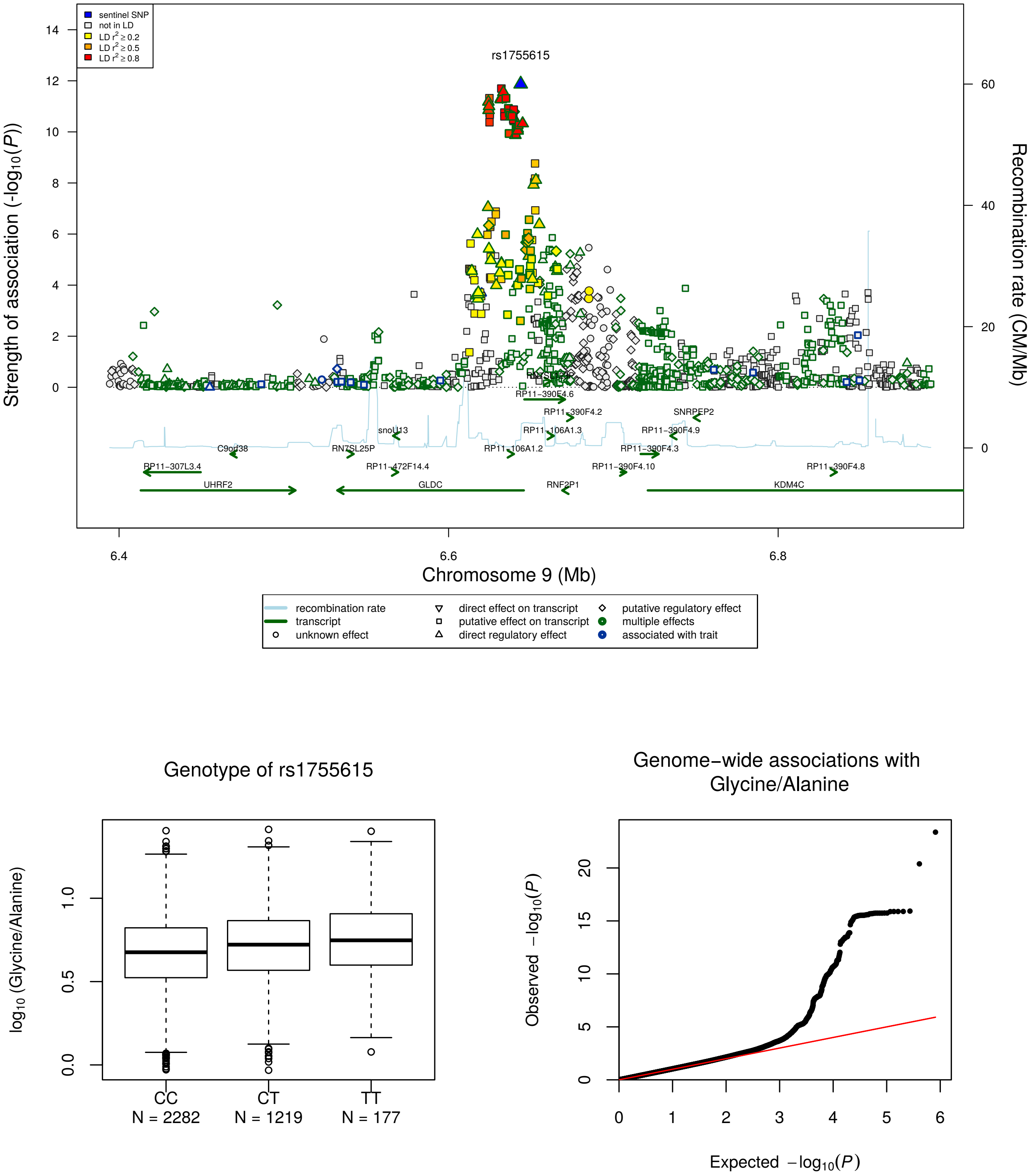

GWAS with **targeted** metabolic traits - **SLC6A13** locus

Associations with 3-Aminoisobutyrate

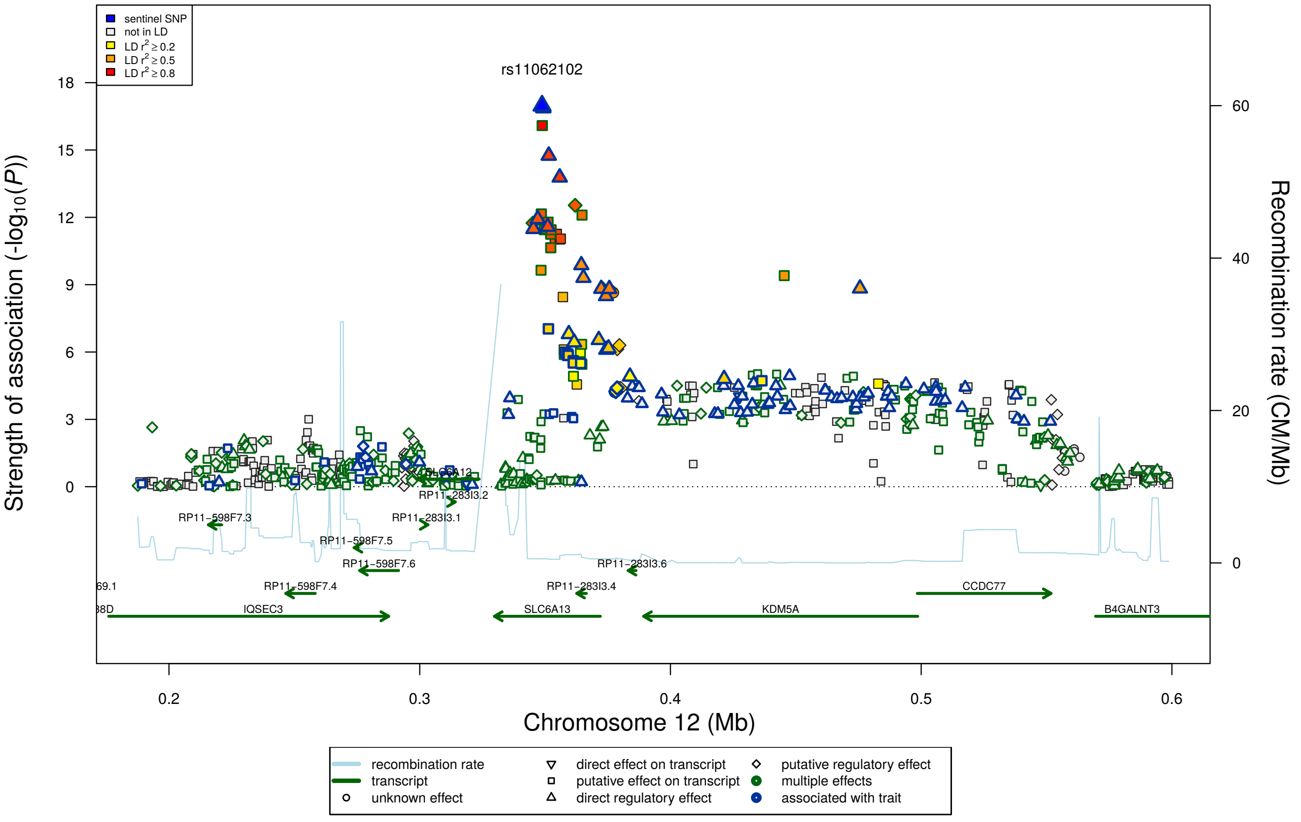

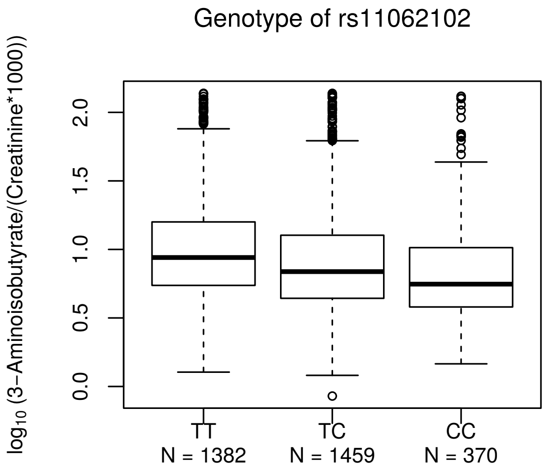

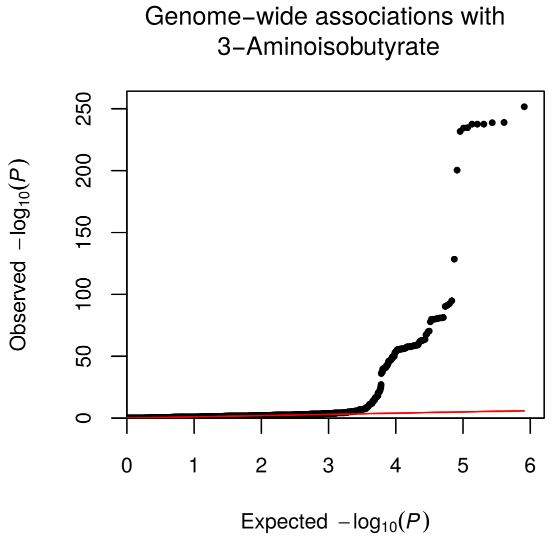

GWAS with **targeted** metabolic traits - **HPD** locus

Associations with 2-Hydroxyisobutyrate

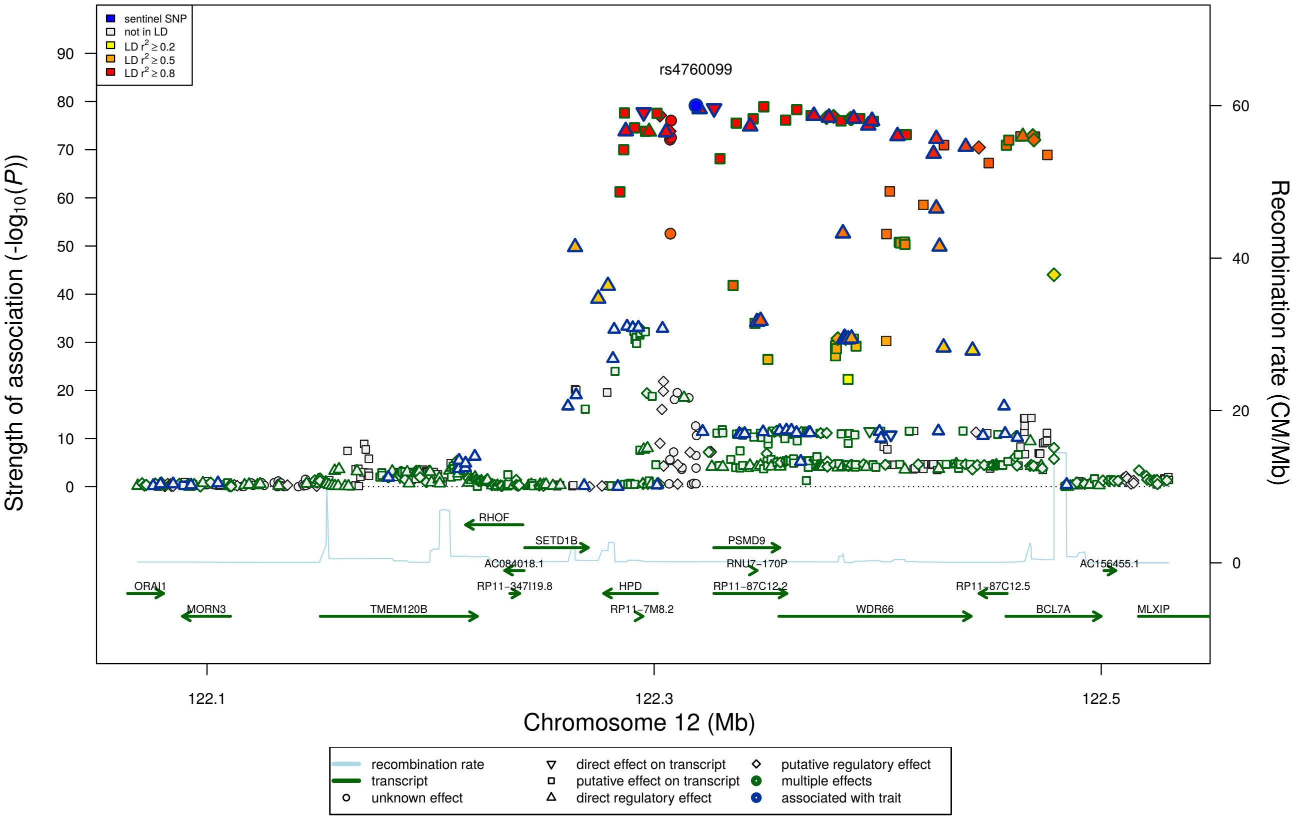

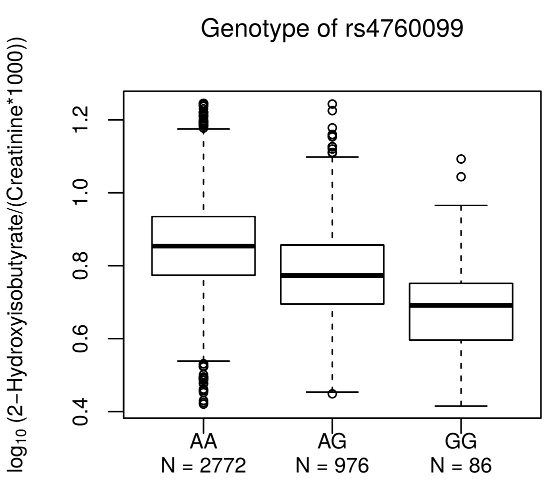

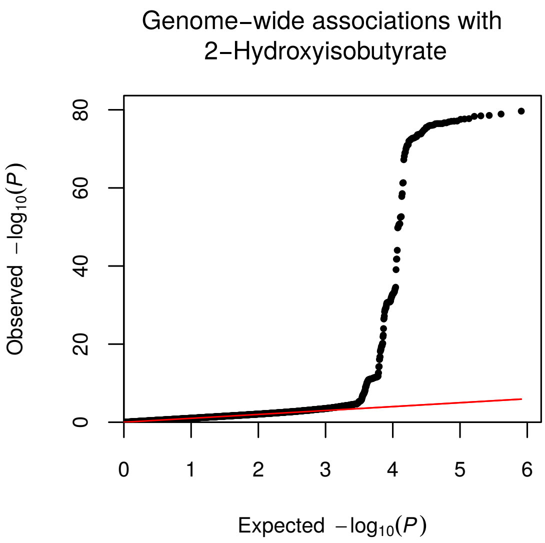

GWAS with **targeted** metabolic traits - **PNMT** locus

Associations with Histidine/tau-Methylhistidine

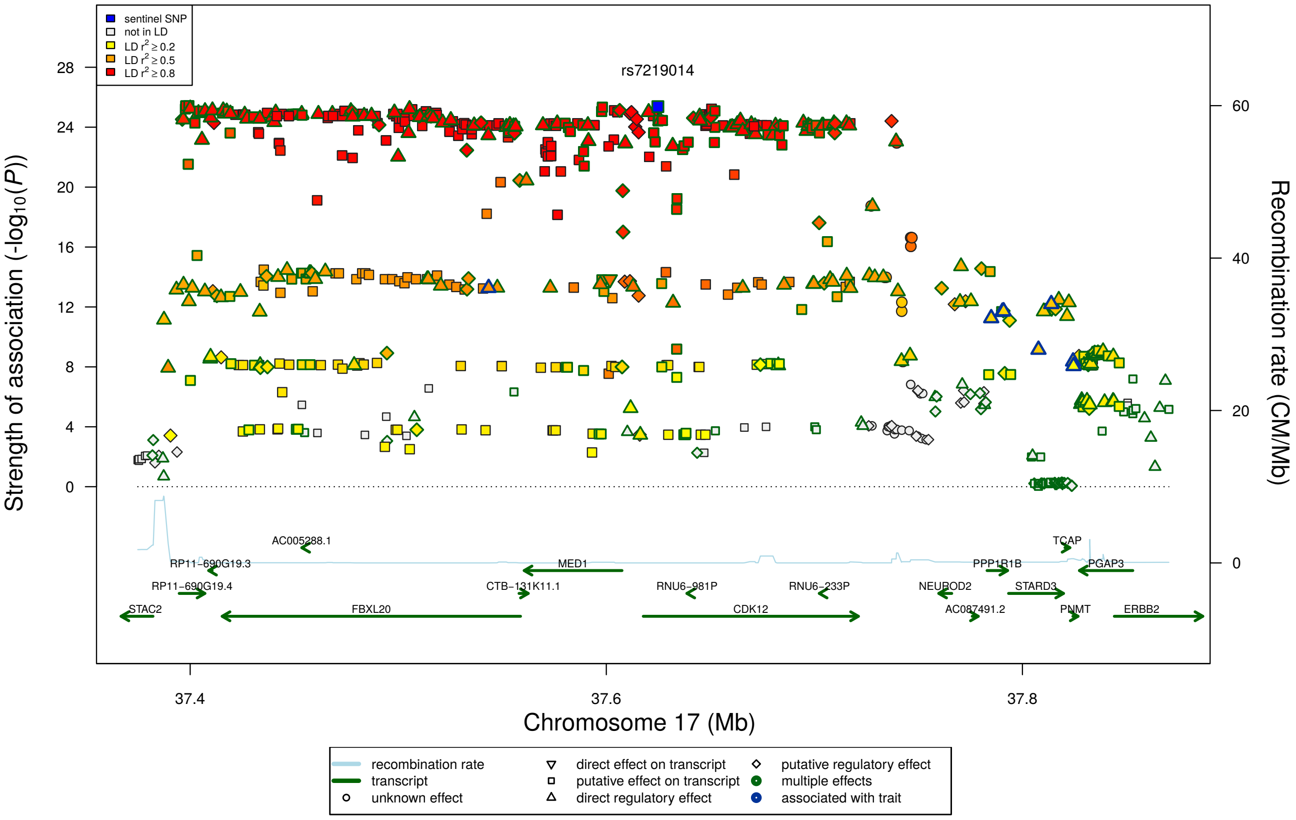

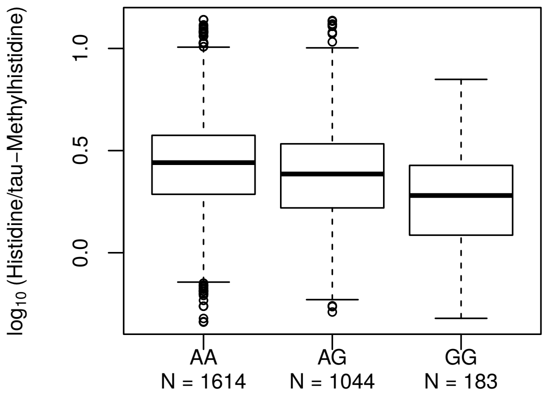

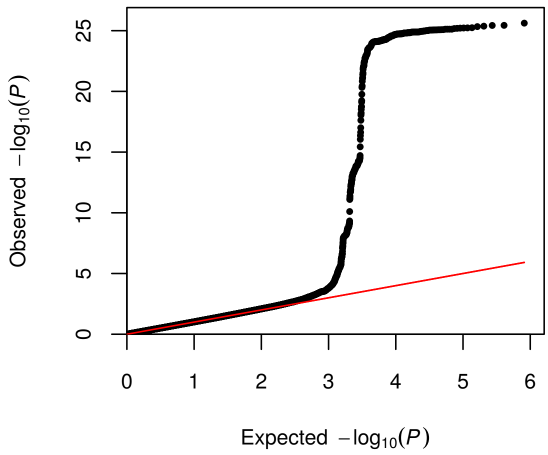

GWAS with **targeted** metabolic traits - **SLC7A9** locus

Associations with Lysine

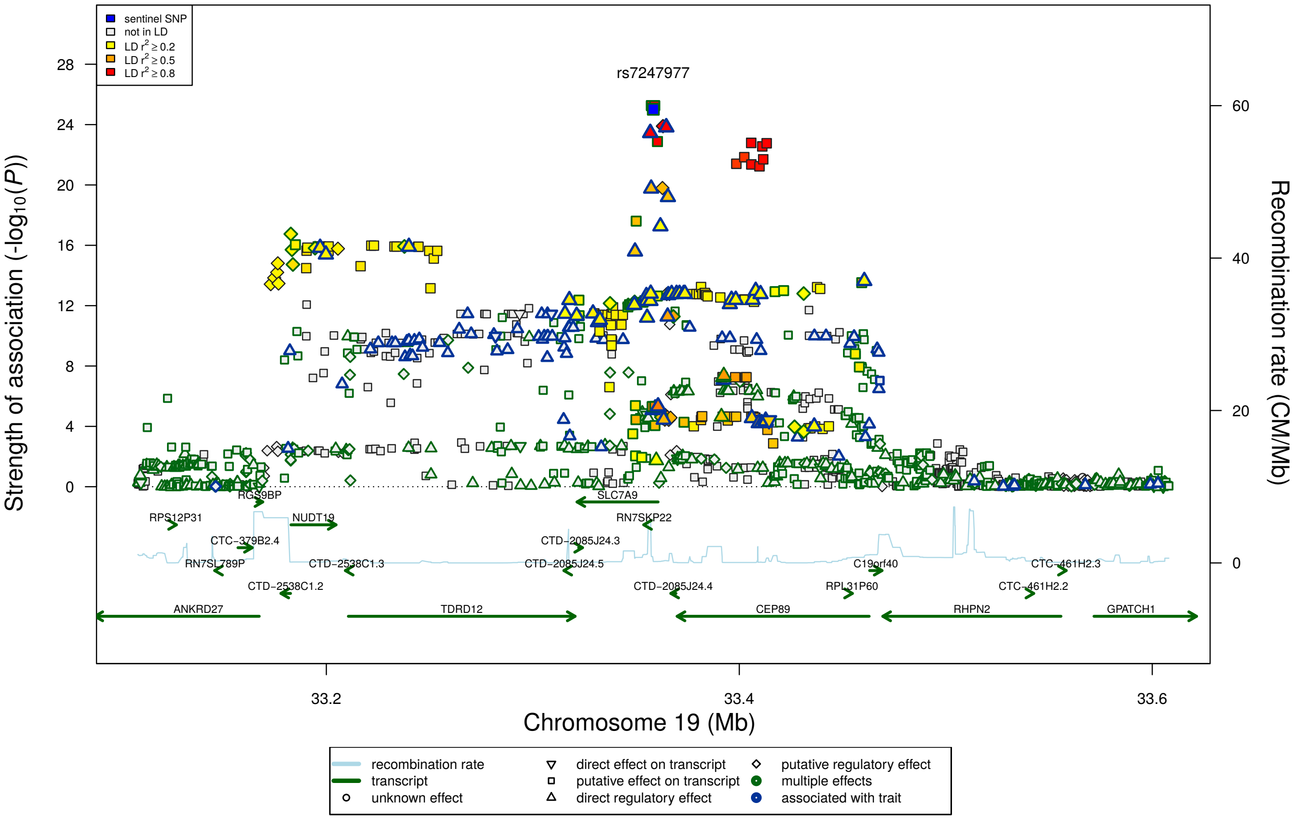

Genotype of rs7247977

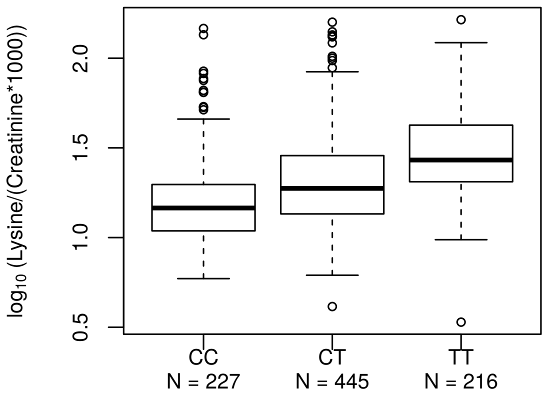

Genome−wide associations with Lysine

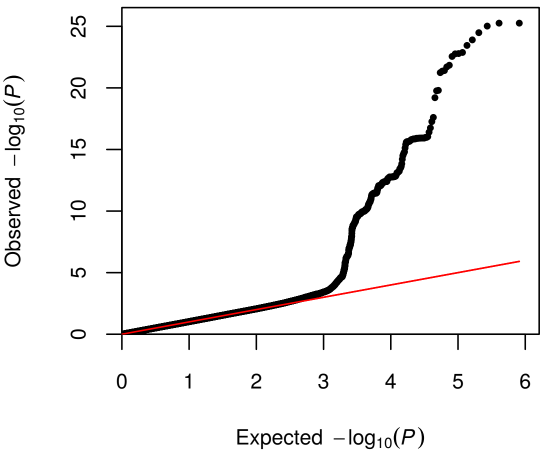

GWAS with **targeted** metabolic traits - **SLC13A3** locus

Associations with Succinate/Citrate

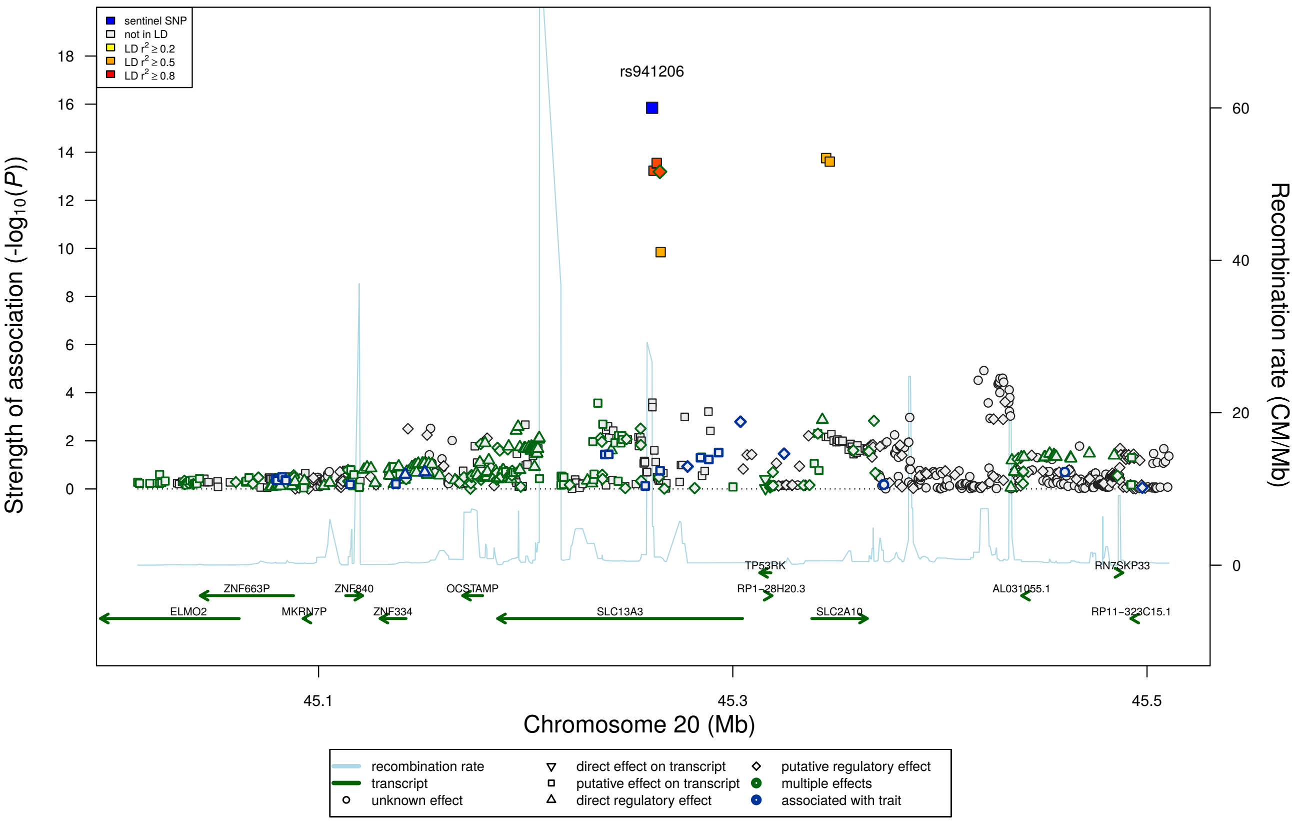

Genotype of rs941206

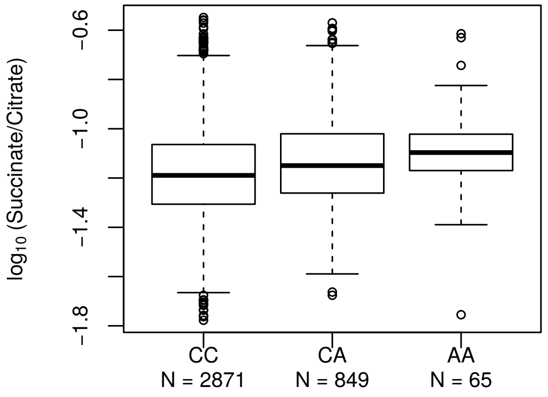

Genome−wide associations with Succinate/Citrate

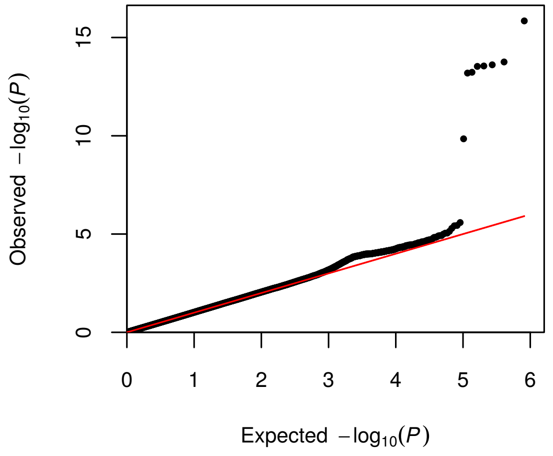

GWAS with **non-targeted** metabolic traits - **NAT8** locus

Associations with 2.0308 ppm

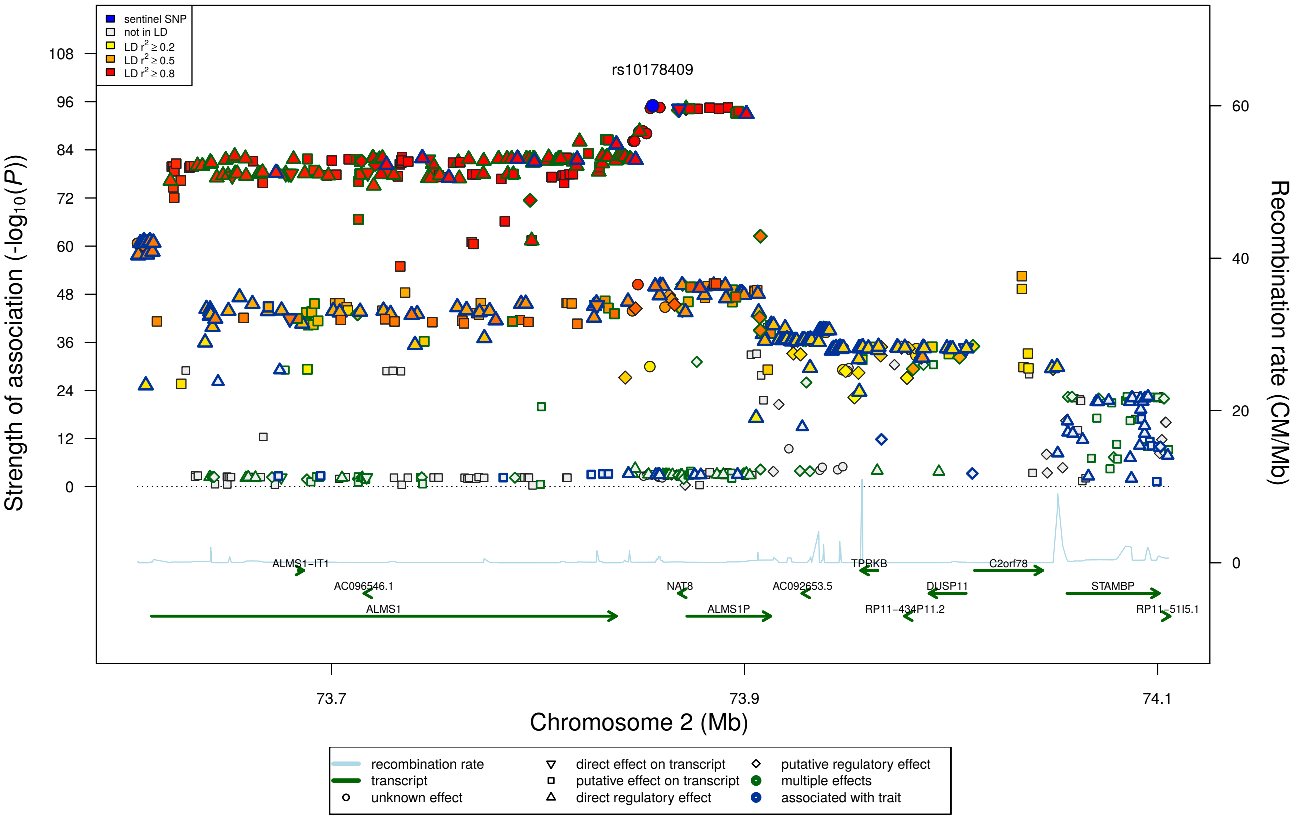

Genotype of rs10178409

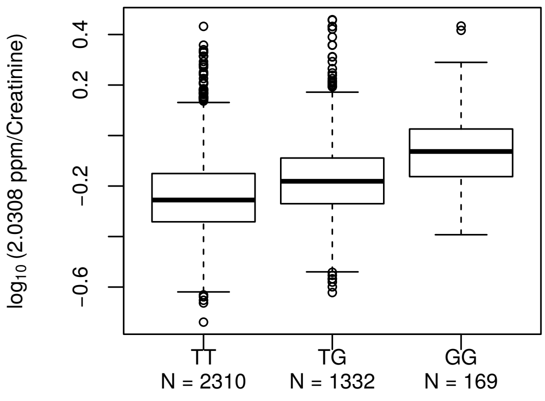

Genome-wide associations with 2.0308 ppm

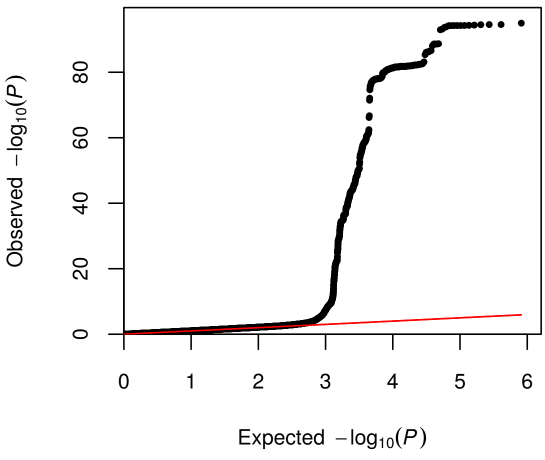

GWAS with **non-targeted** metabolic traits - **HIBCH** locus

Associations with 1.0666/1.0489 ppm

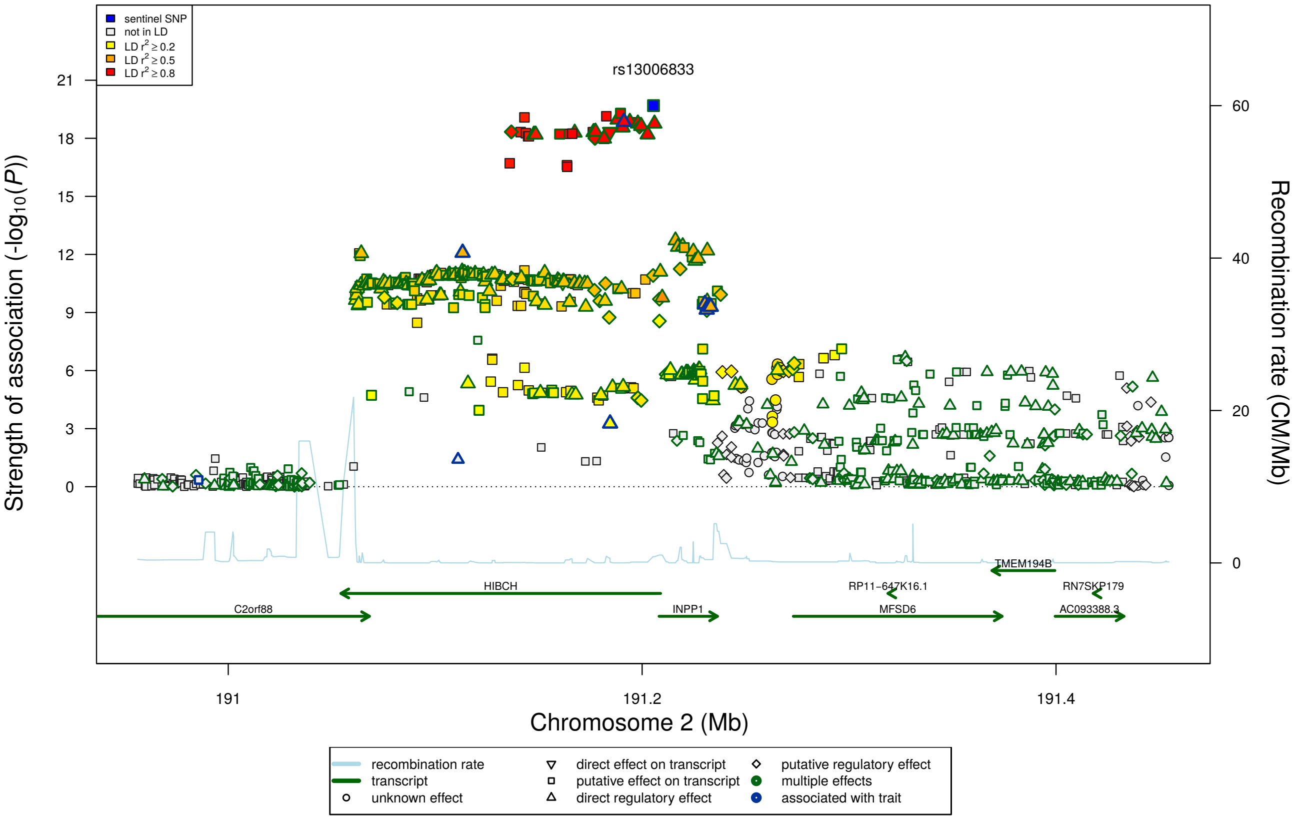

Genotype of rs13006833

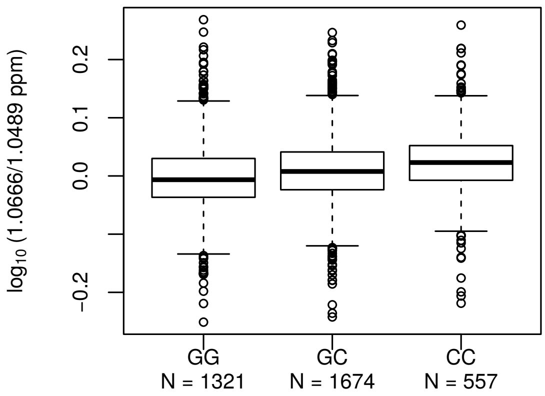

Genome−wide associations with 1.0666/1.0489 ppm

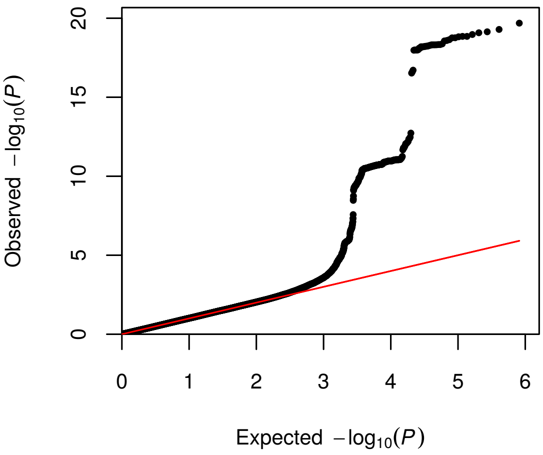

GWAS with **non-targeted** metabolic traits - **CPS1** locus

Associations with 3.5549/2.5468 ppm

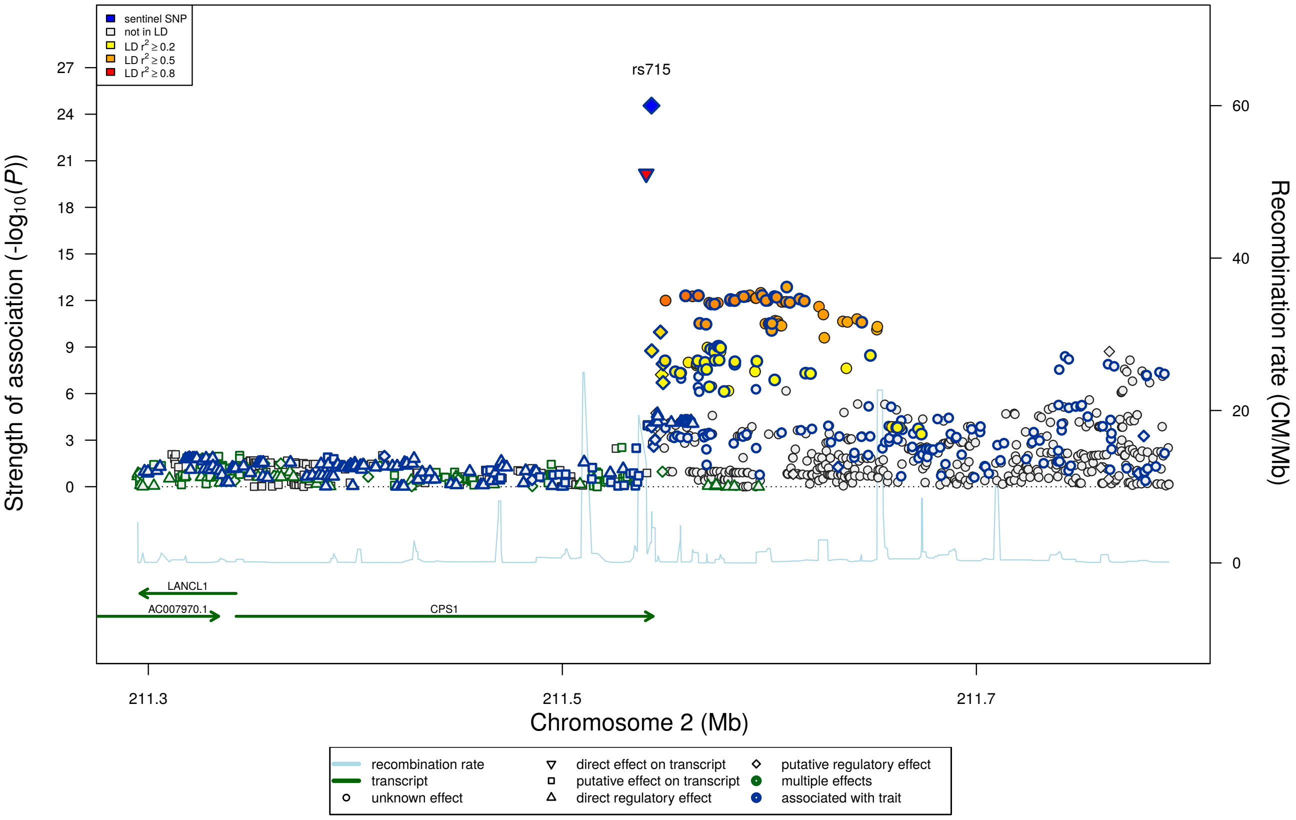

Genotype of rs715

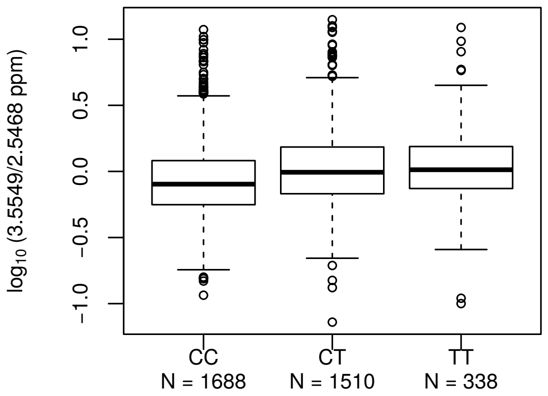

Genome−wide associations with 3.5549/2.5468 ppm

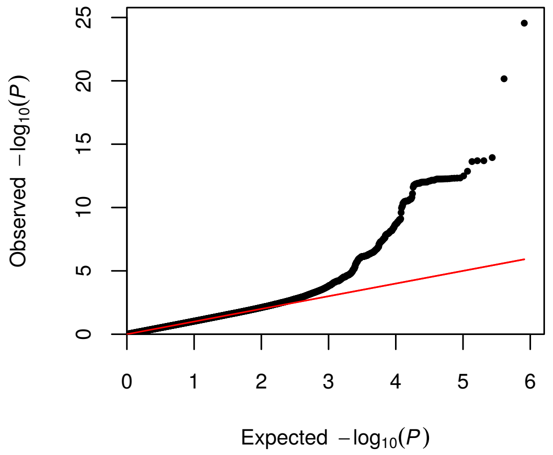

GWAS with **non-targeted** metabolic traits - **AGXT** locus

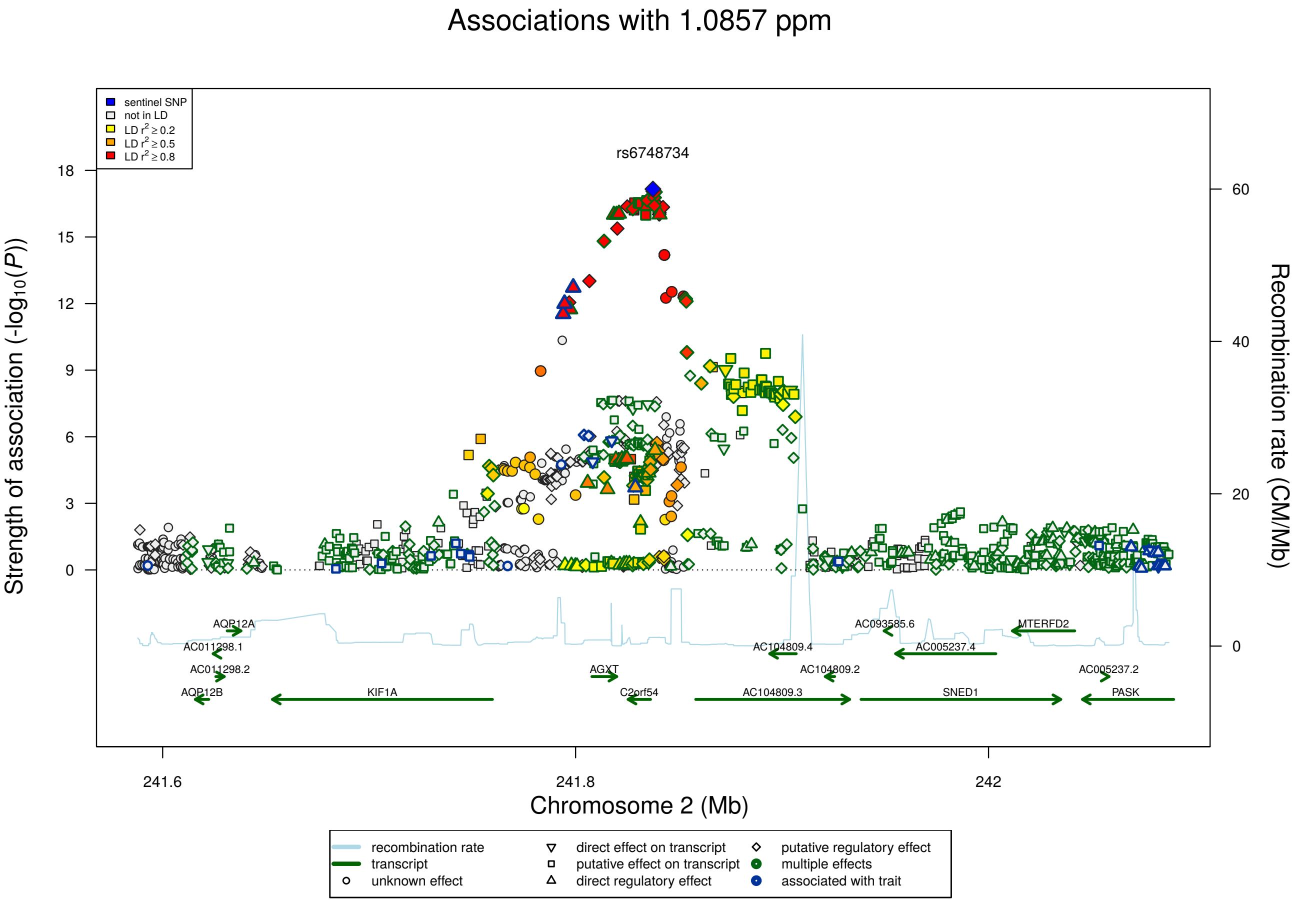

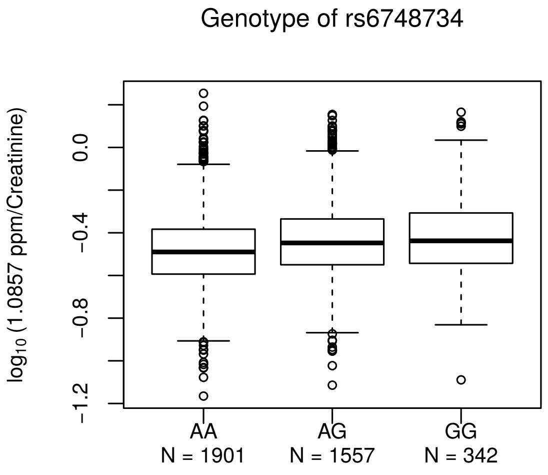

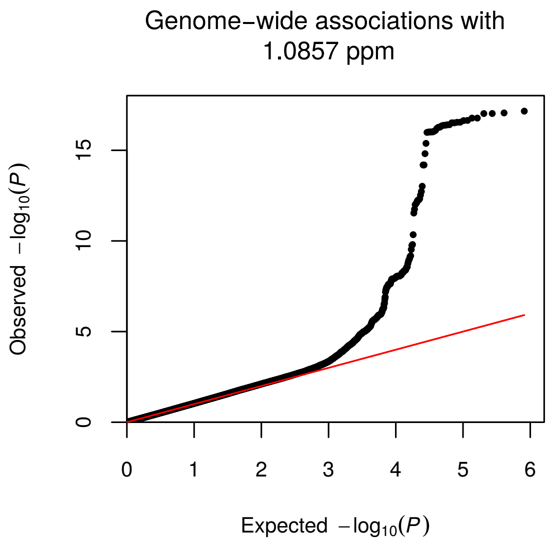

GWAS with **non-targeted** metabolic traits - **SLC6A20** locus

Associations with 2.9156 ppm

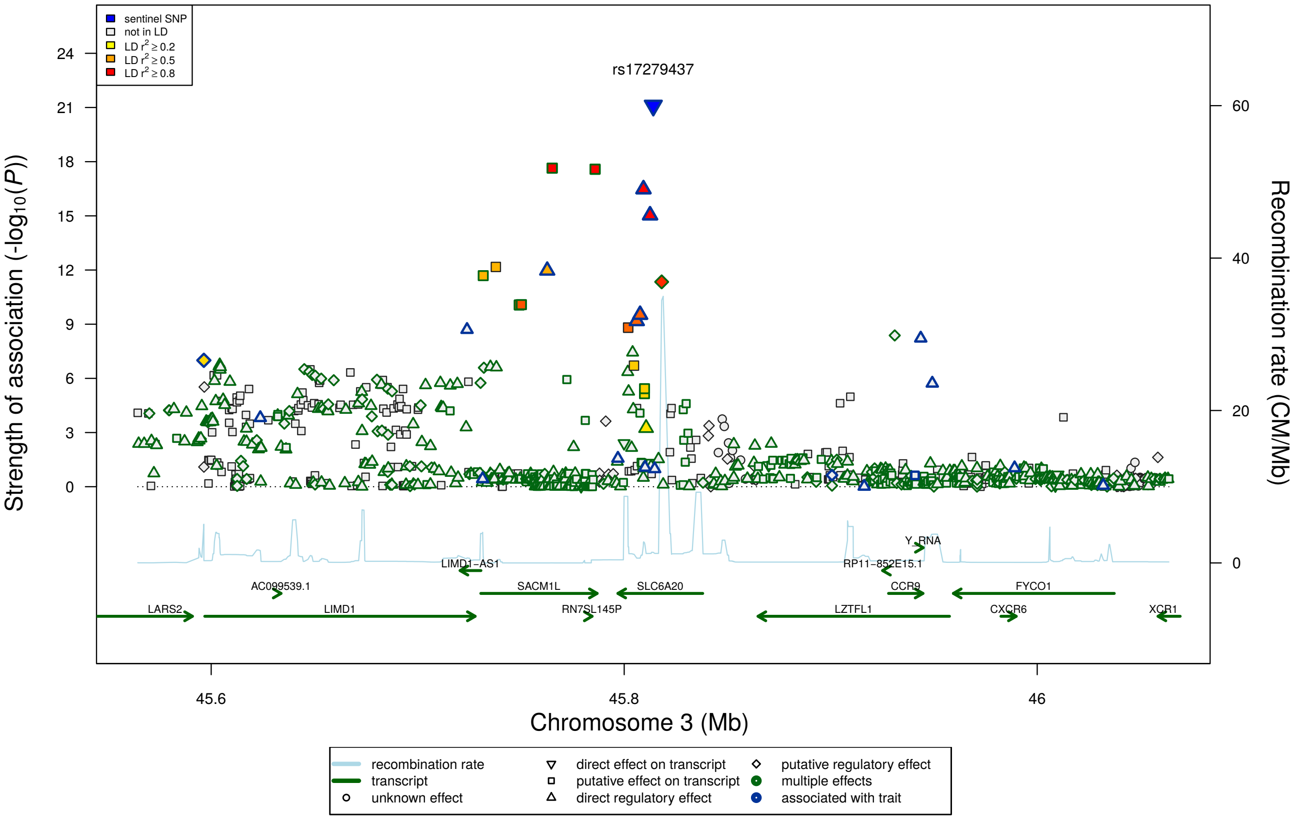

Genotype of rs17279437

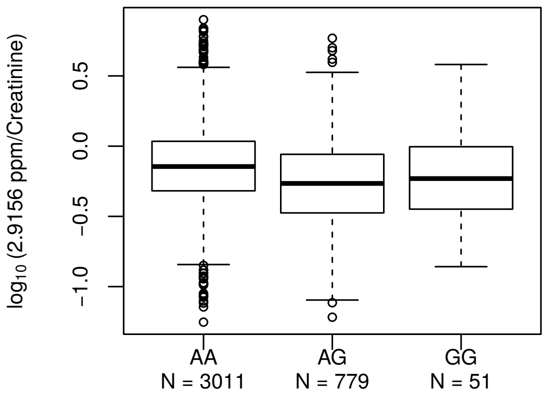

Genome−wide associations with 2.9156 ppm

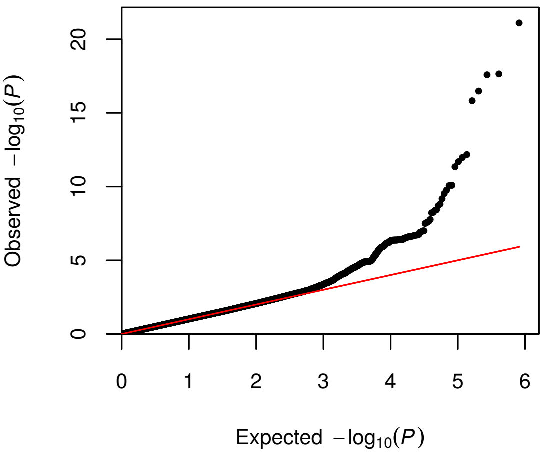

GWAS with **non-targeted** metabolic traits - **TKT** locus

Associations with 4.0935/7.6643 ppm

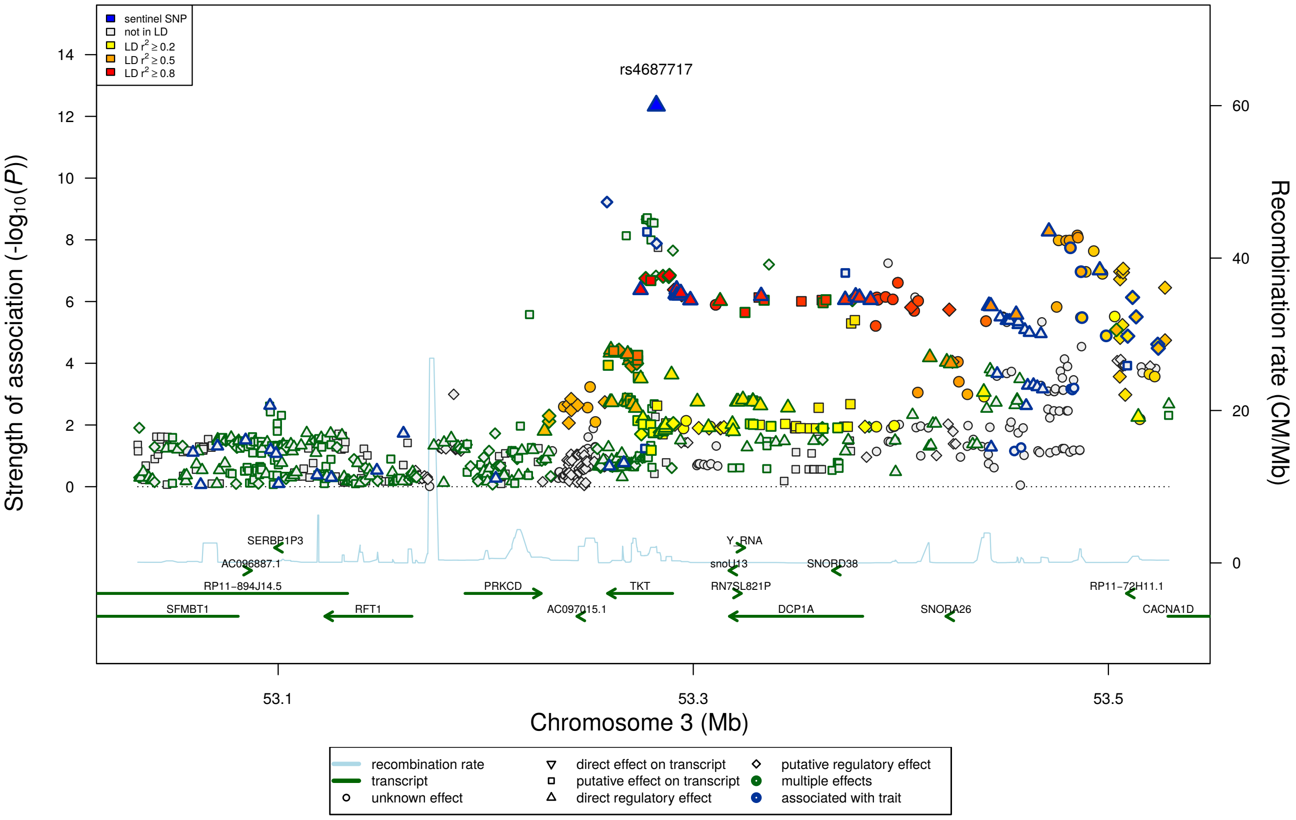

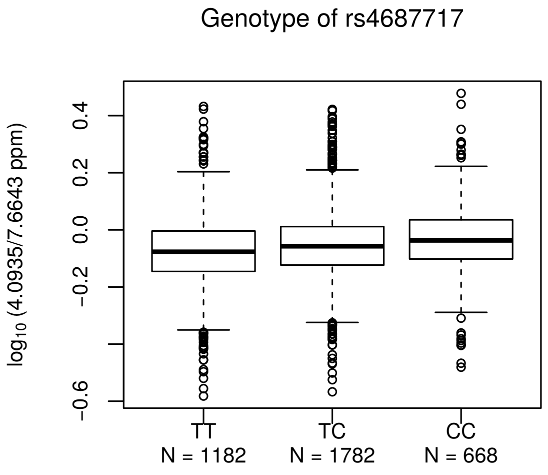

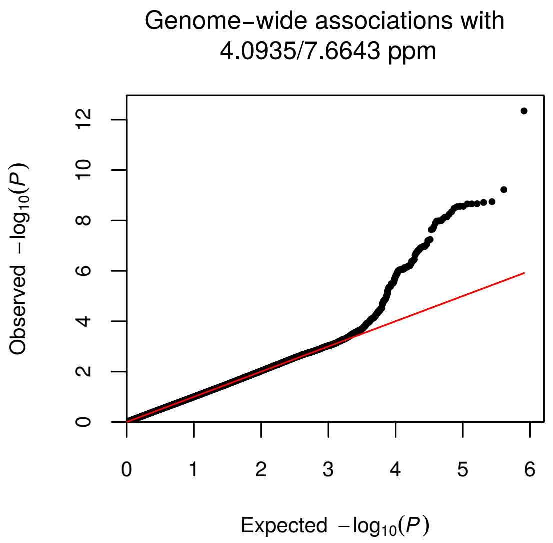

GWAS with **non-targeted** metabolic traits - **ETNPPL** locus

Associations with 3.1263 ppm

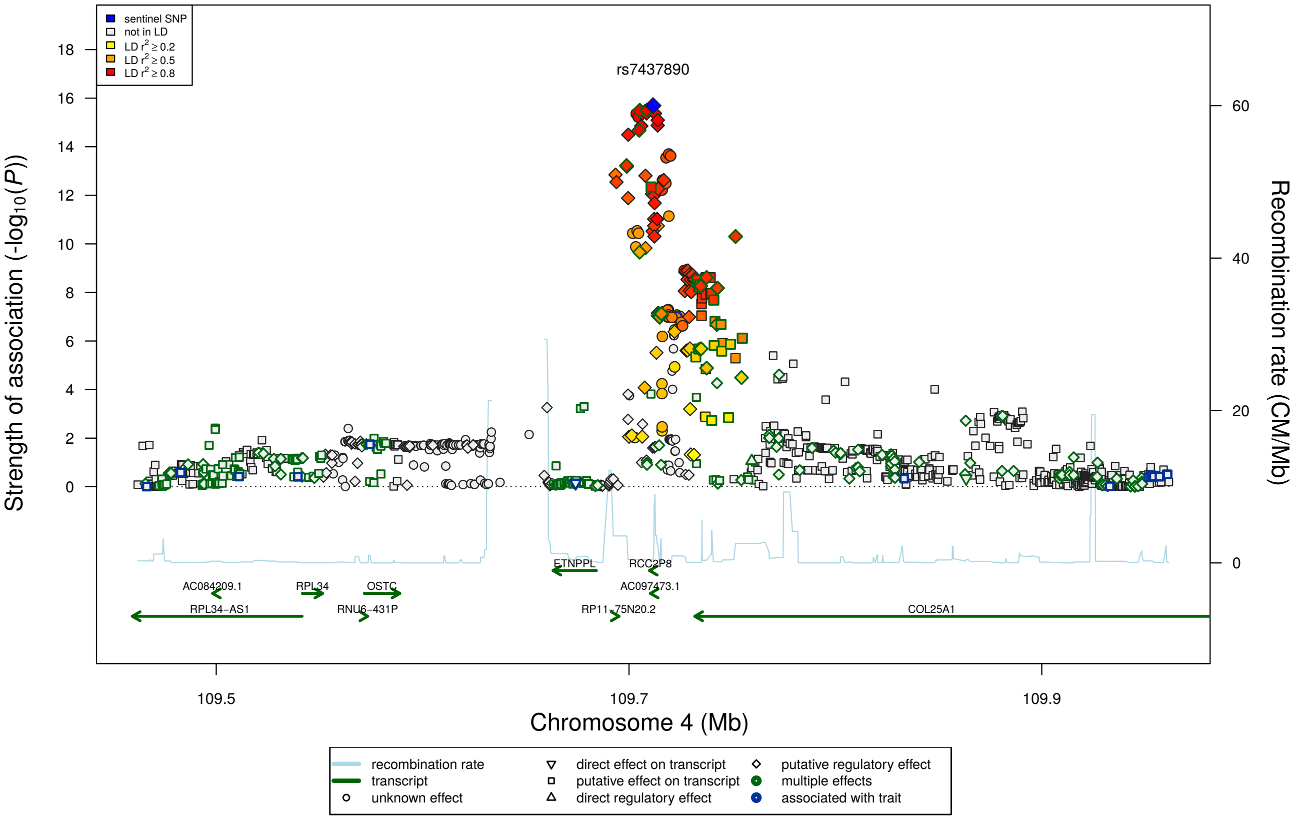

Genotype of rs7437890

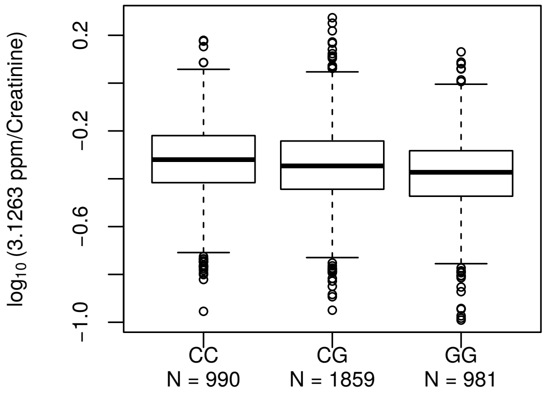

Genome−wide associations with 3.1263 ppm

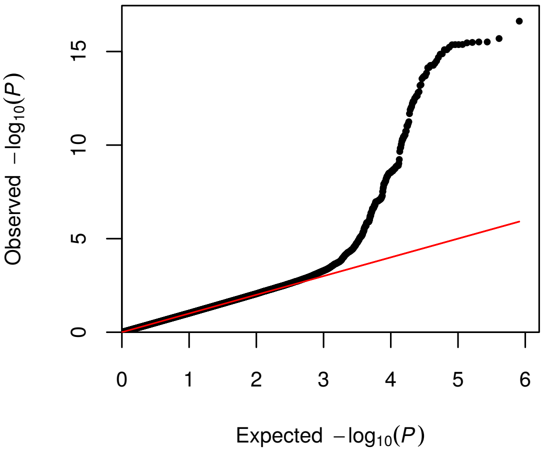

GWAS with **non-targeted** metabolic traits - **SLC6A19** locus

Associations with 6.8769 ppm

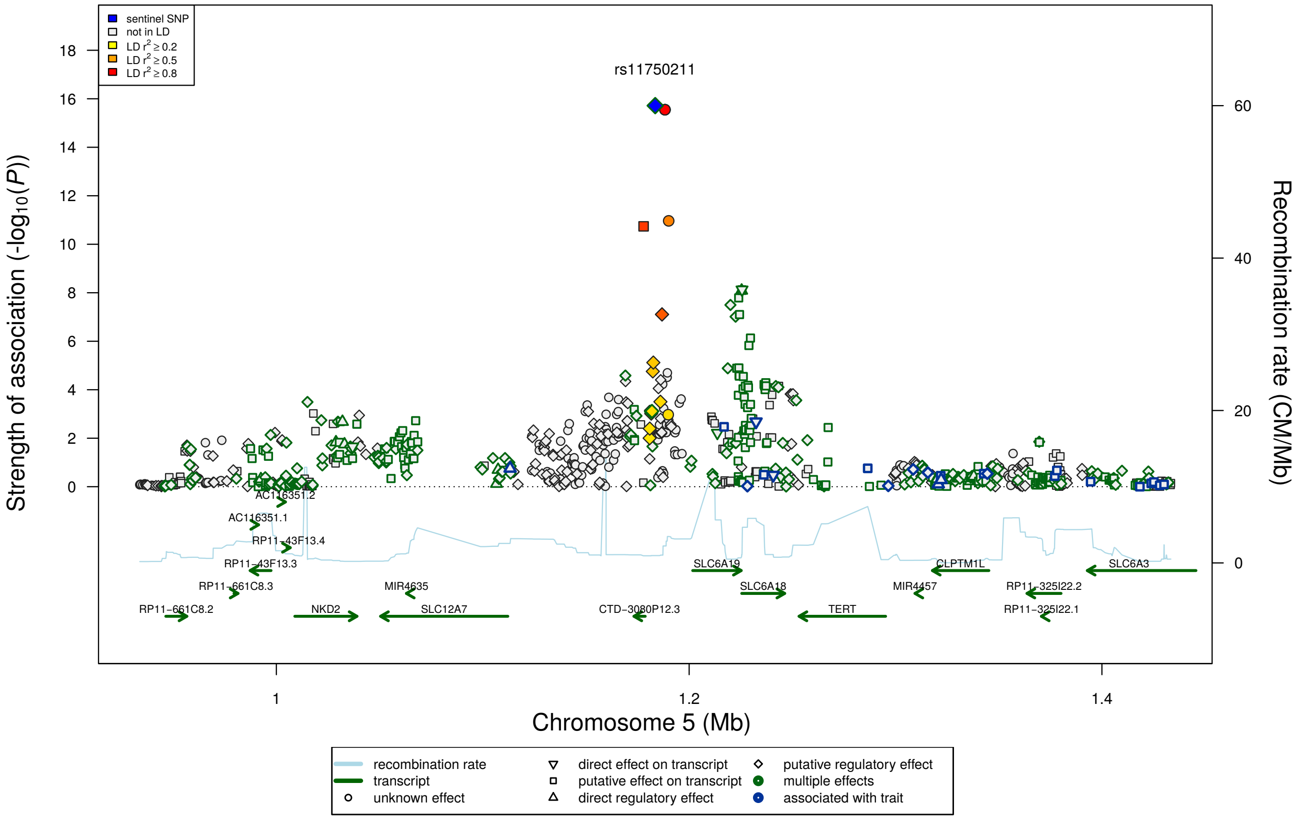

Genotype of rs11750211

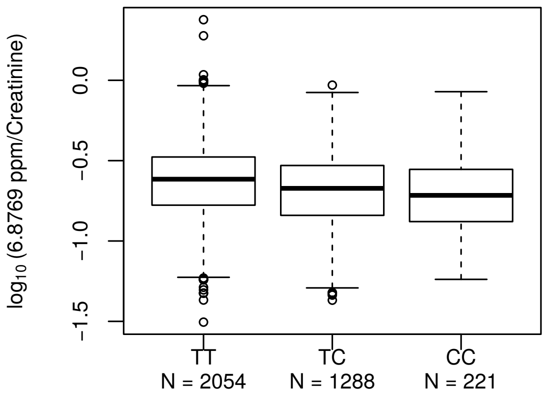

Genome−wide associations with 6.8769 ppm

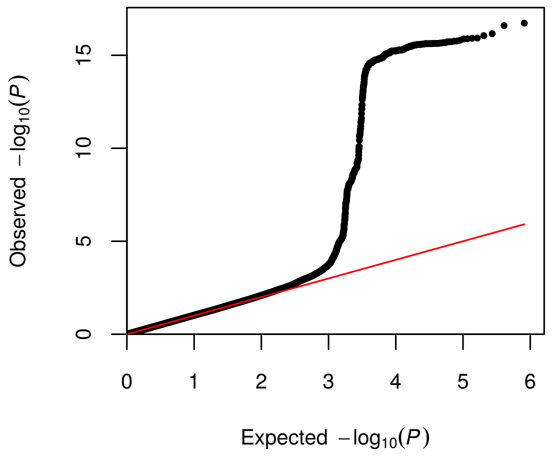

GWAS with **non-targeted** metabolic traits - **AGXT2** locus

Associations with 1.1710/1.9733 ppm

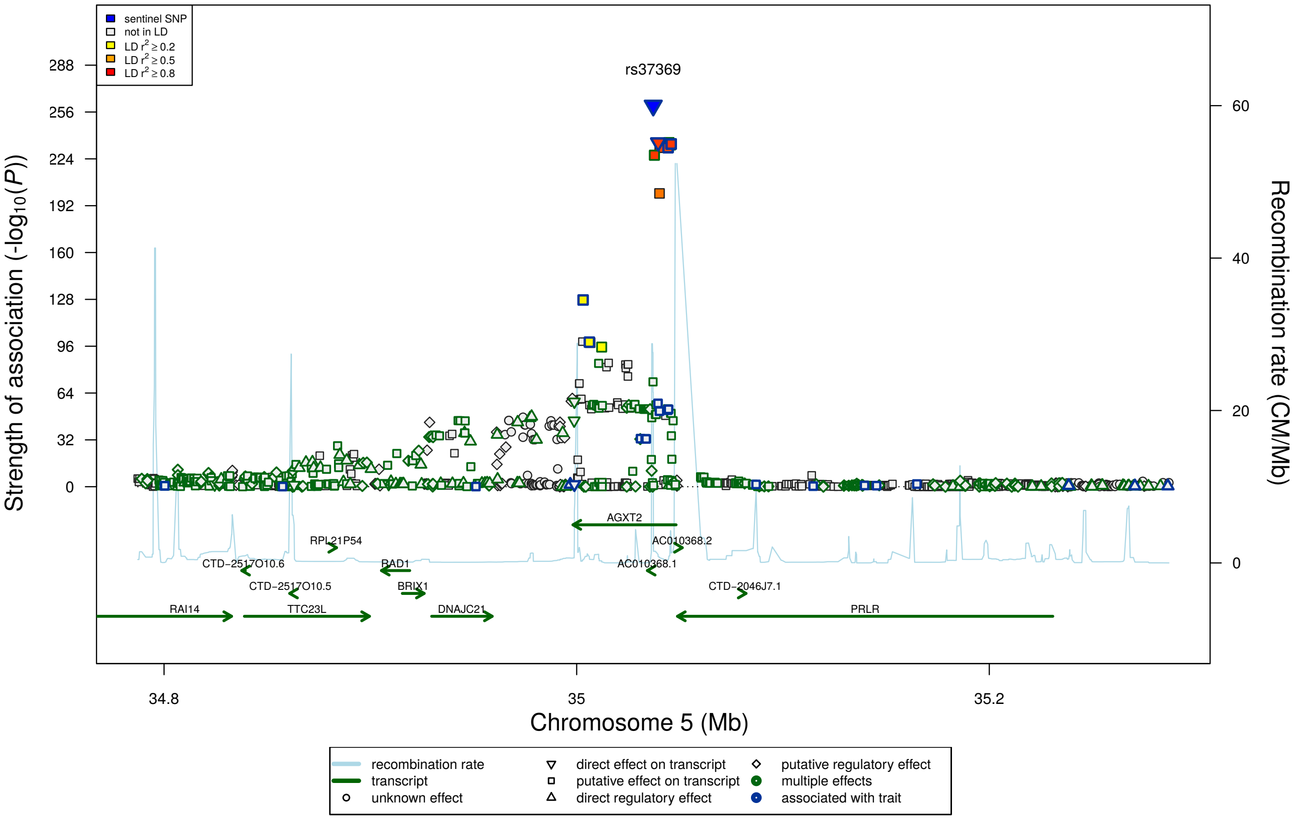

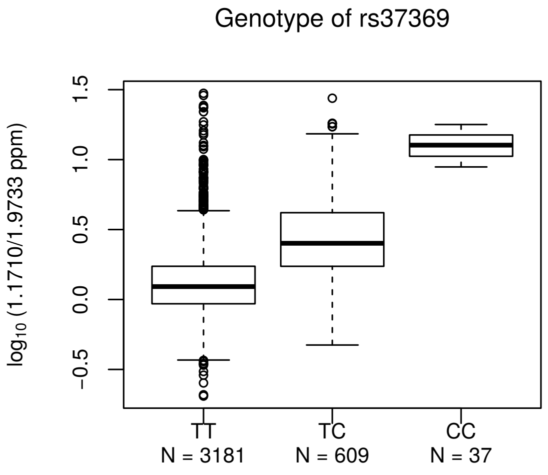

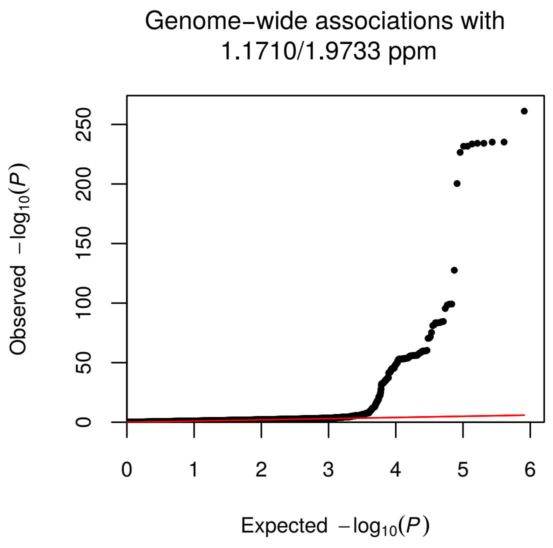

GWAS with **non-targeted** metabolic traits - **DMGDH** locus

Associations with 2.9156/5.2355 ppm

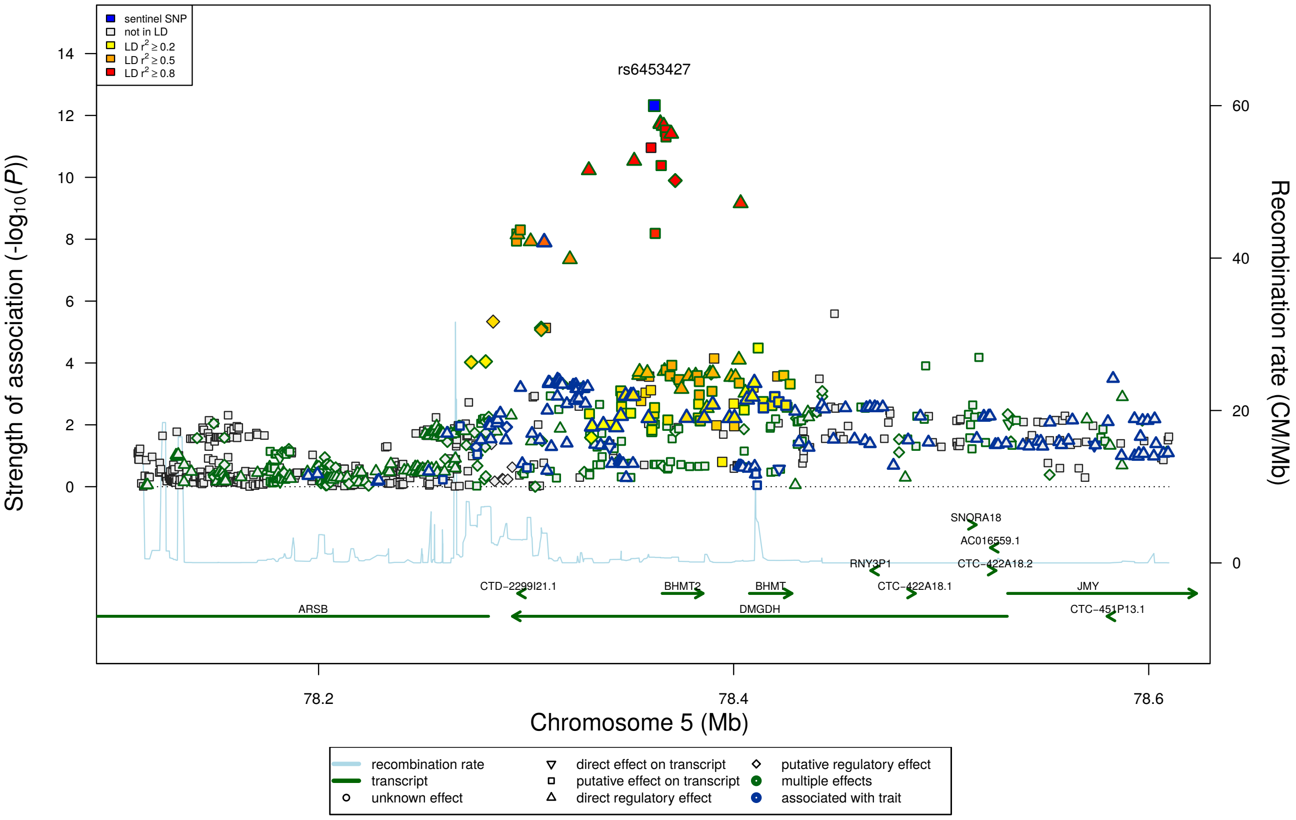

Genotype of rs6453427

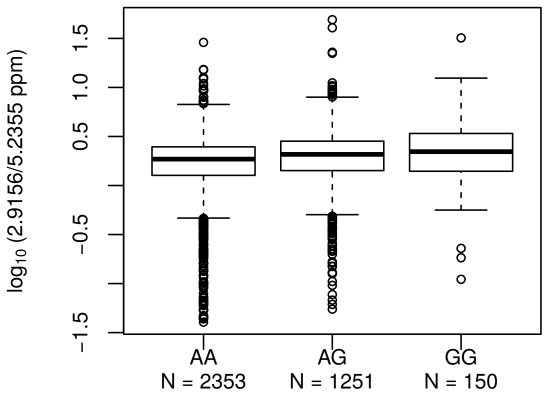

Genome−wide associations with 2.9156/5.2355 ppm

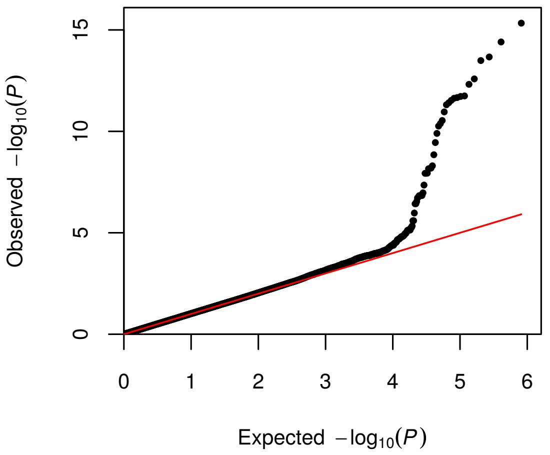

GWAS with **non-targeted** metabolic traits - **SLC36A2** locus

Associations with 3.5549/2.6495 ppm

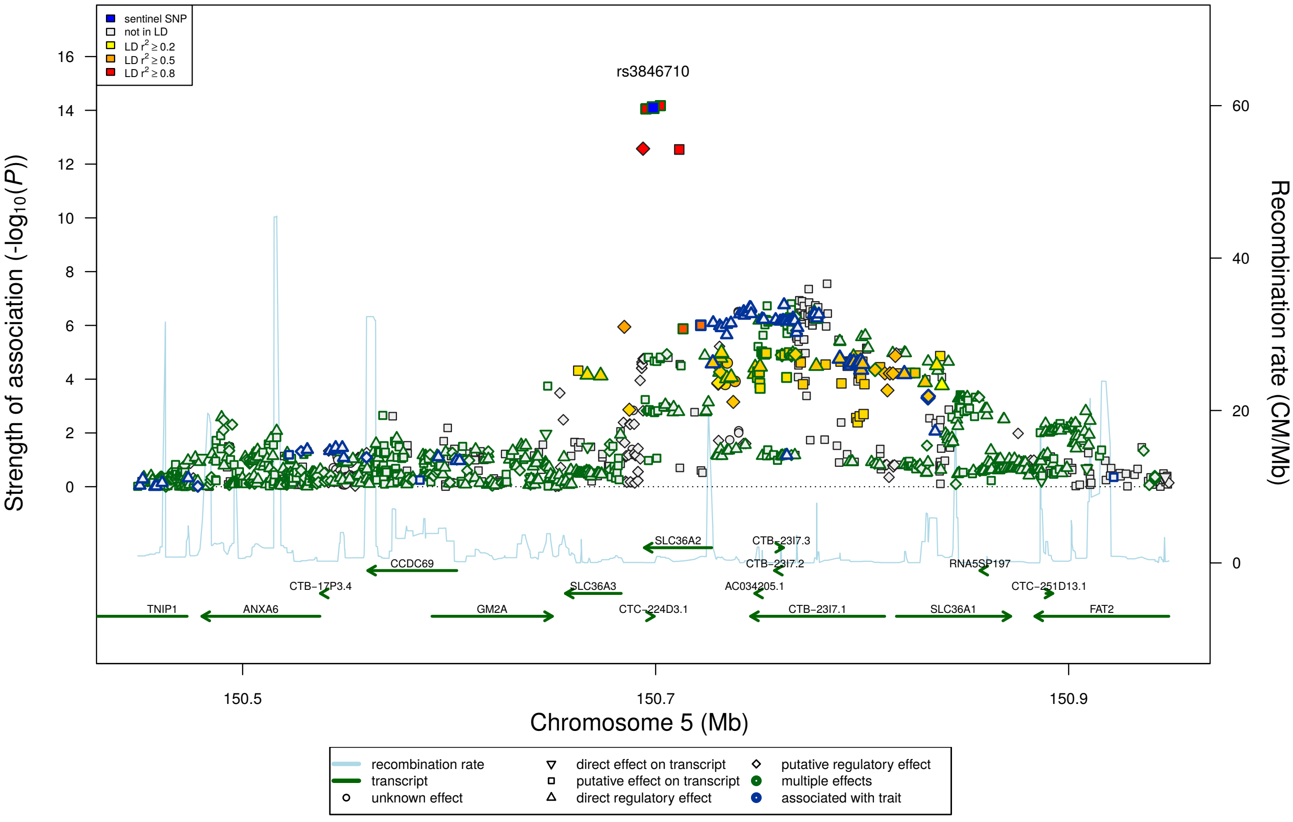

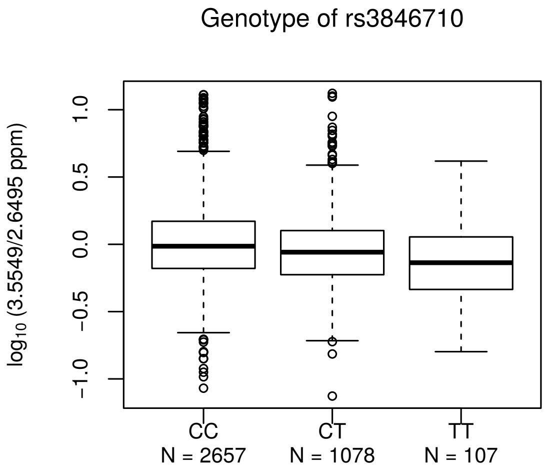

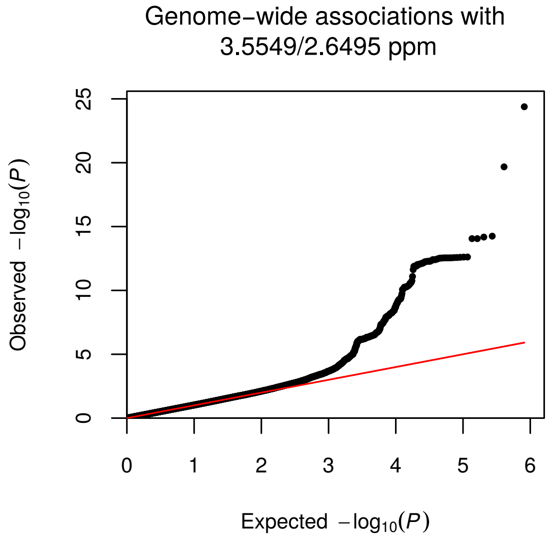

GWAS with **non-targeted** metabolic traits - **NAT2** locus

Associations with 2.1586/3.3240 ppm

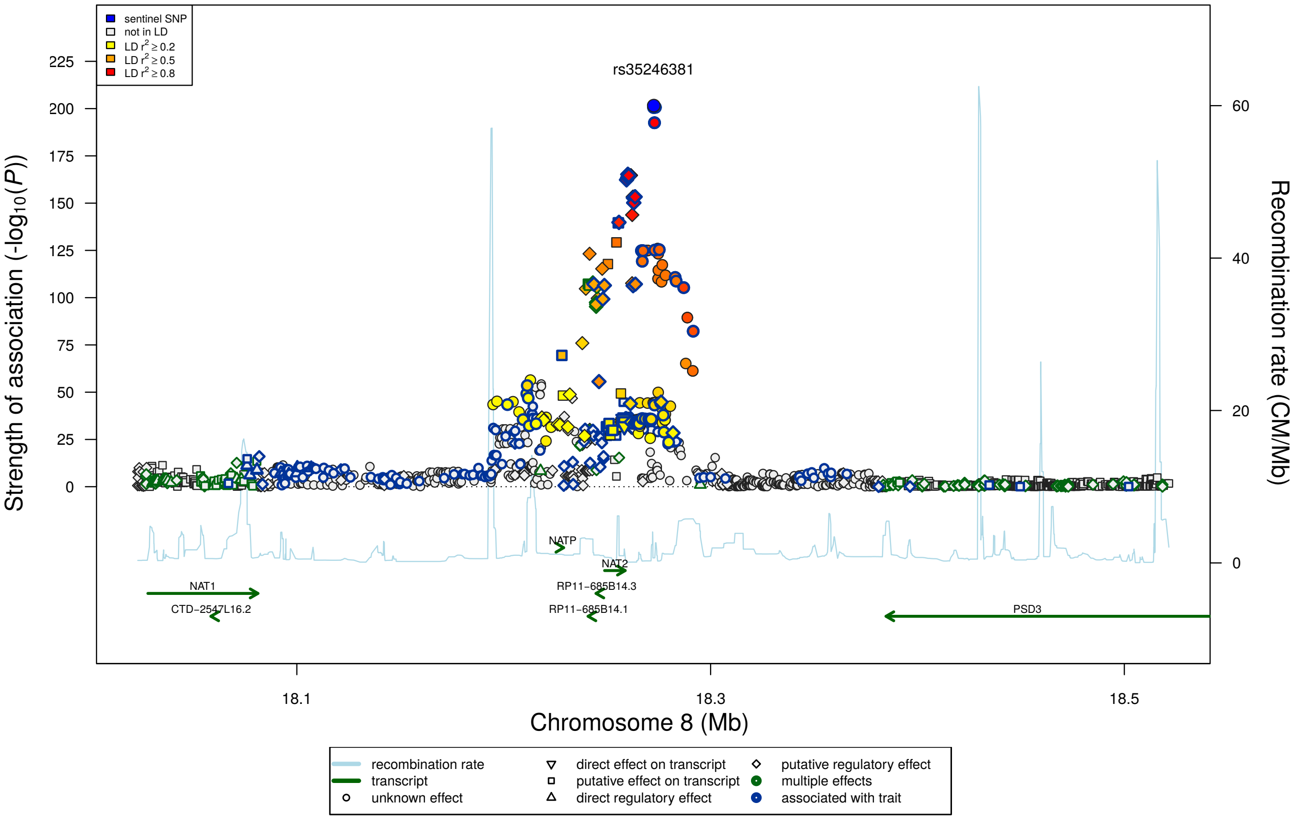

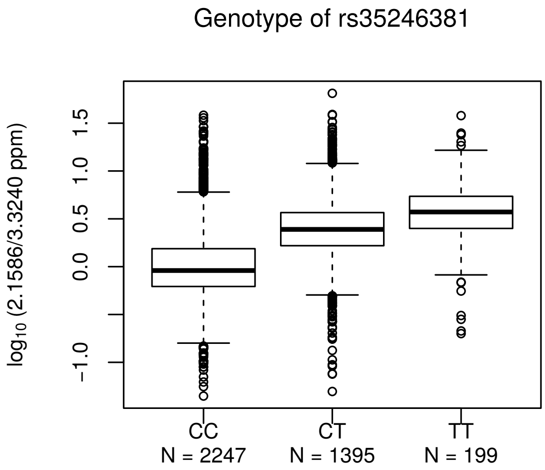

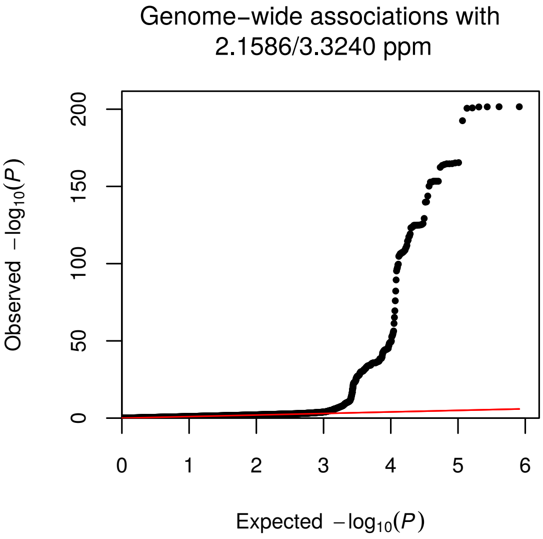

GWAS with **non-targeted** metabolic traits - **ABO** locus

Associations with 2.0308/2.0489 ppm

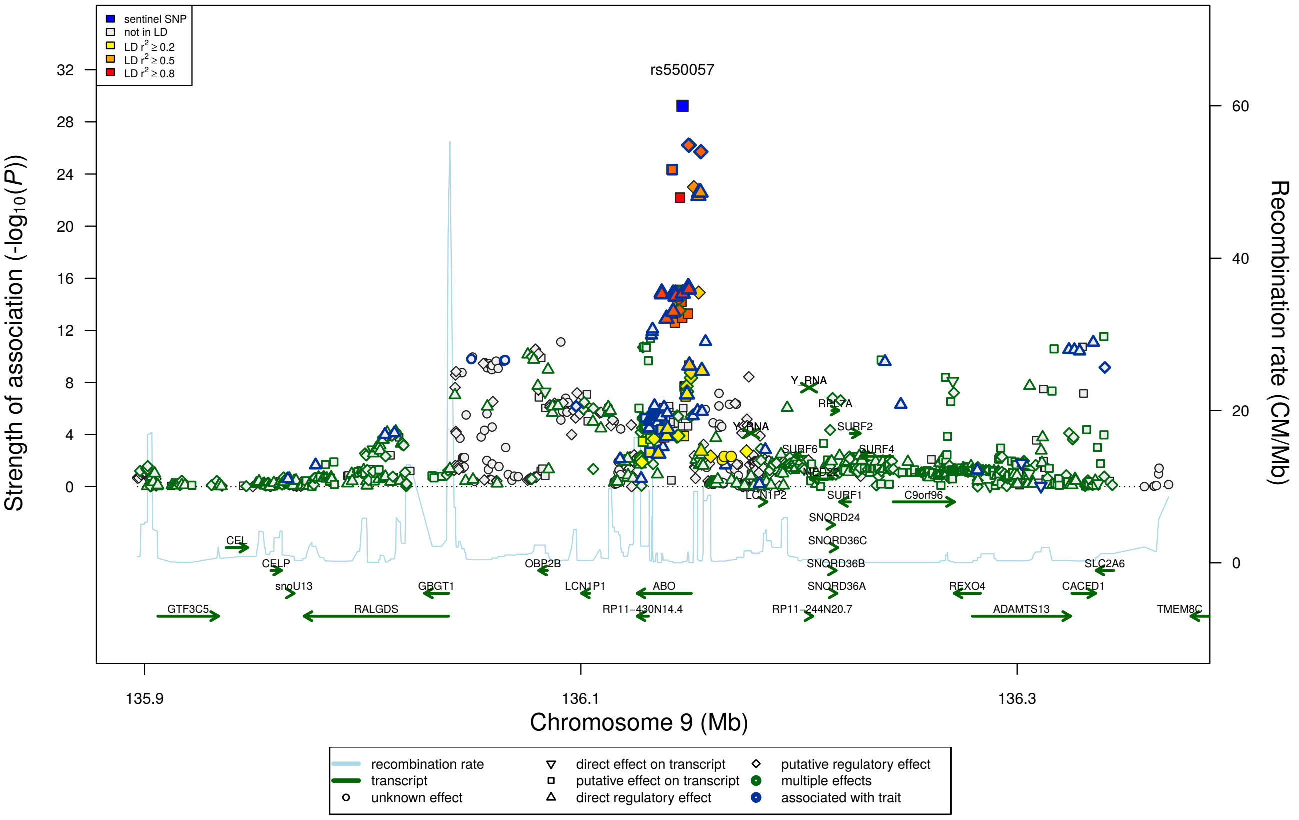

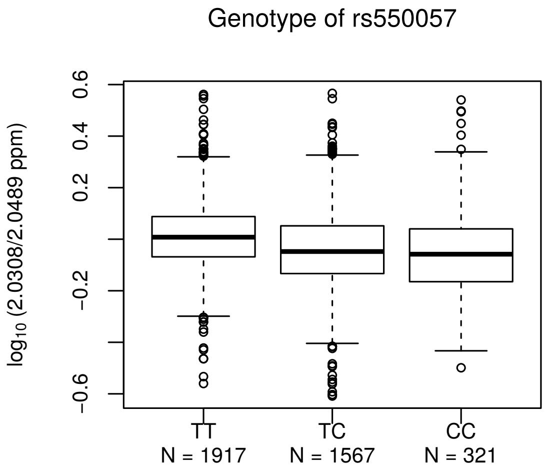

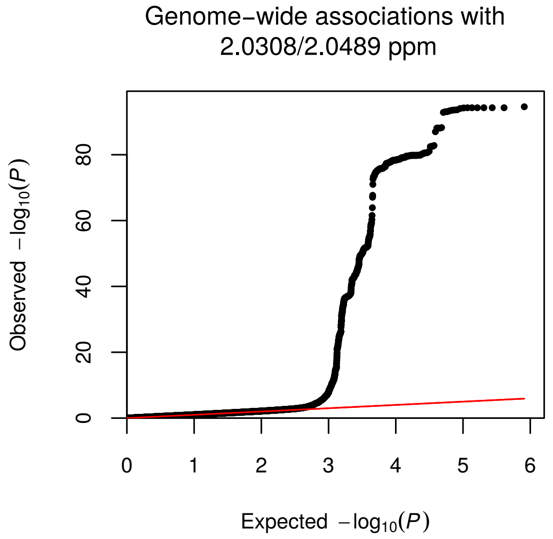

GWAS with **non-targeted** metabolic traits - **PYROXD2** locus

Associations with 2.8541 ppm

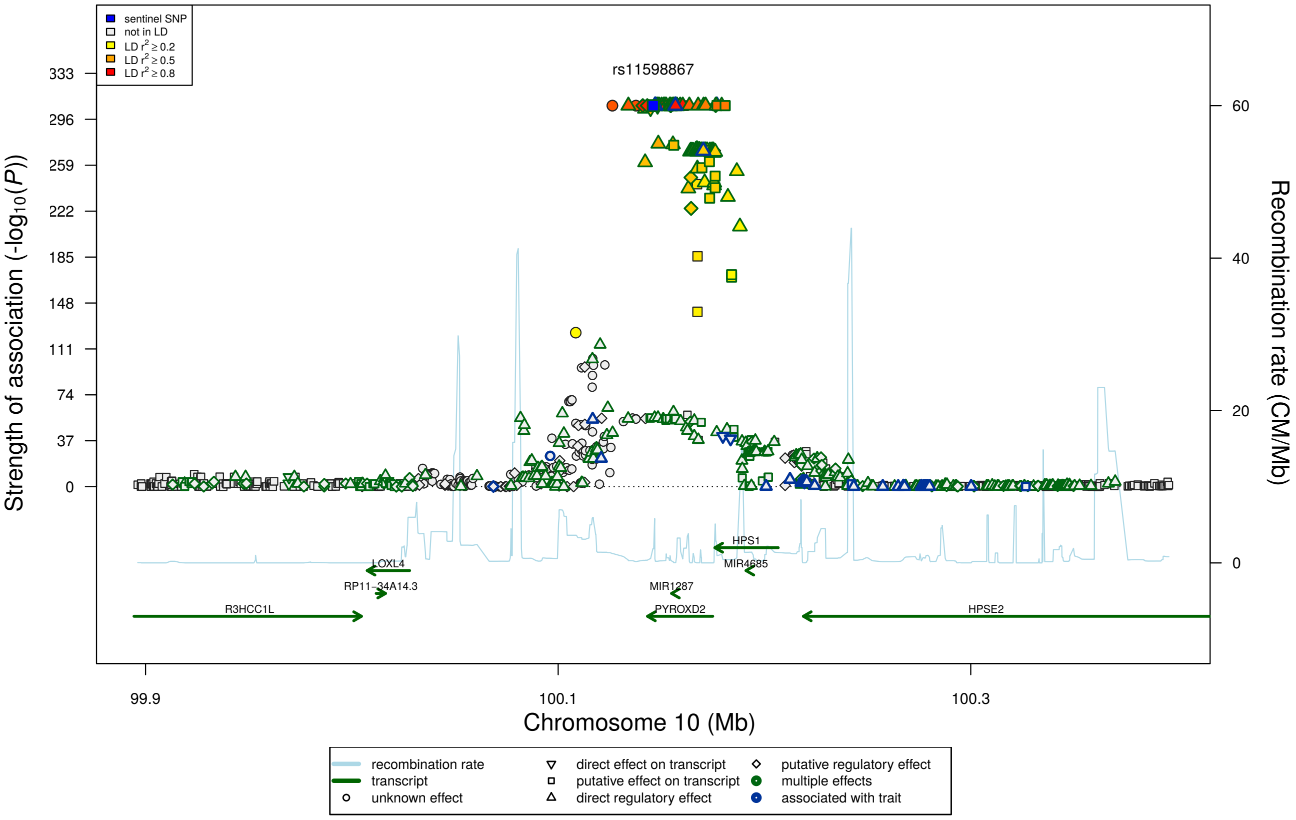

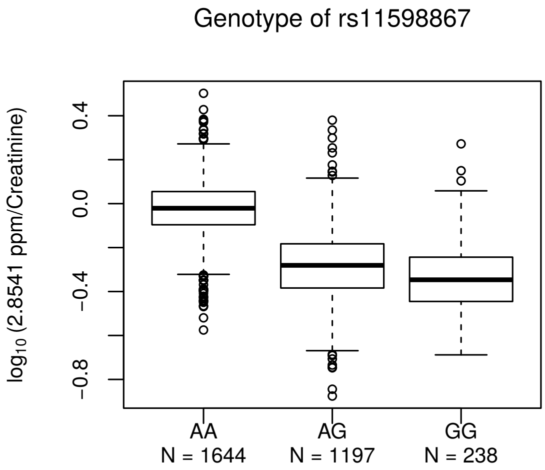

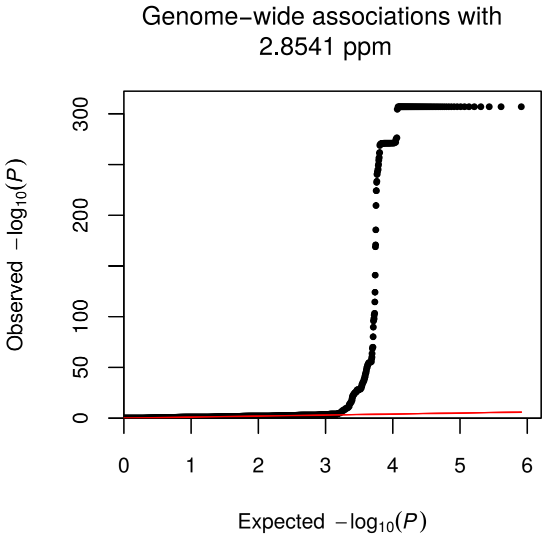

GWAS with **non-targeted** metabolic traits - **SLC6A13** locus

Associations with 1.1895 ppm

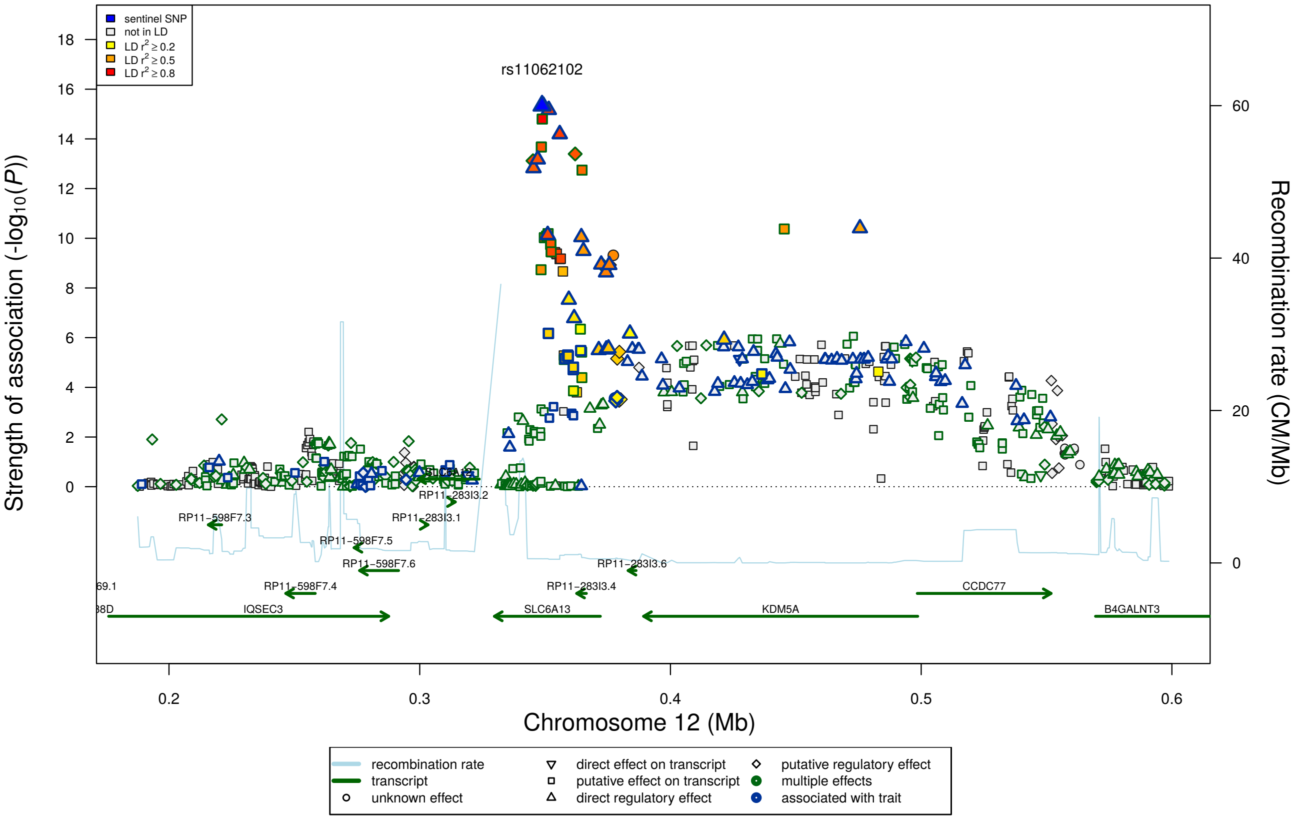

Genotype of rs11062102

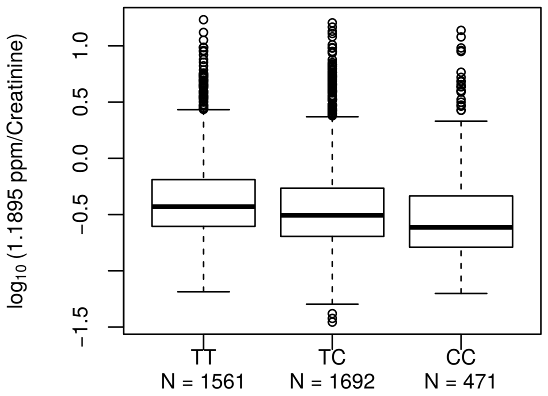

Genome−wide associations with 1.1895 ppm

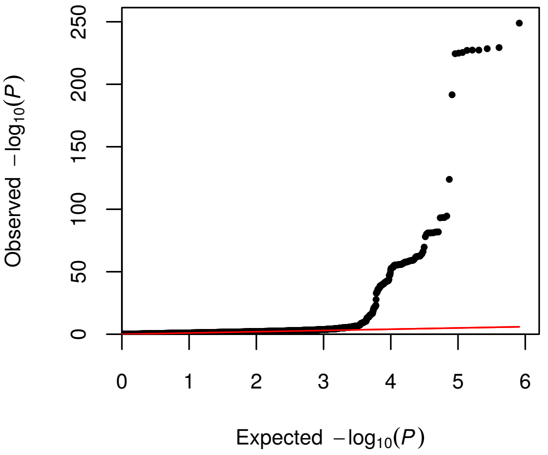

GWAS with **non-targeted** metabolic traits - **HPD** locus

Associations with 1.3455/7.6643 ppm

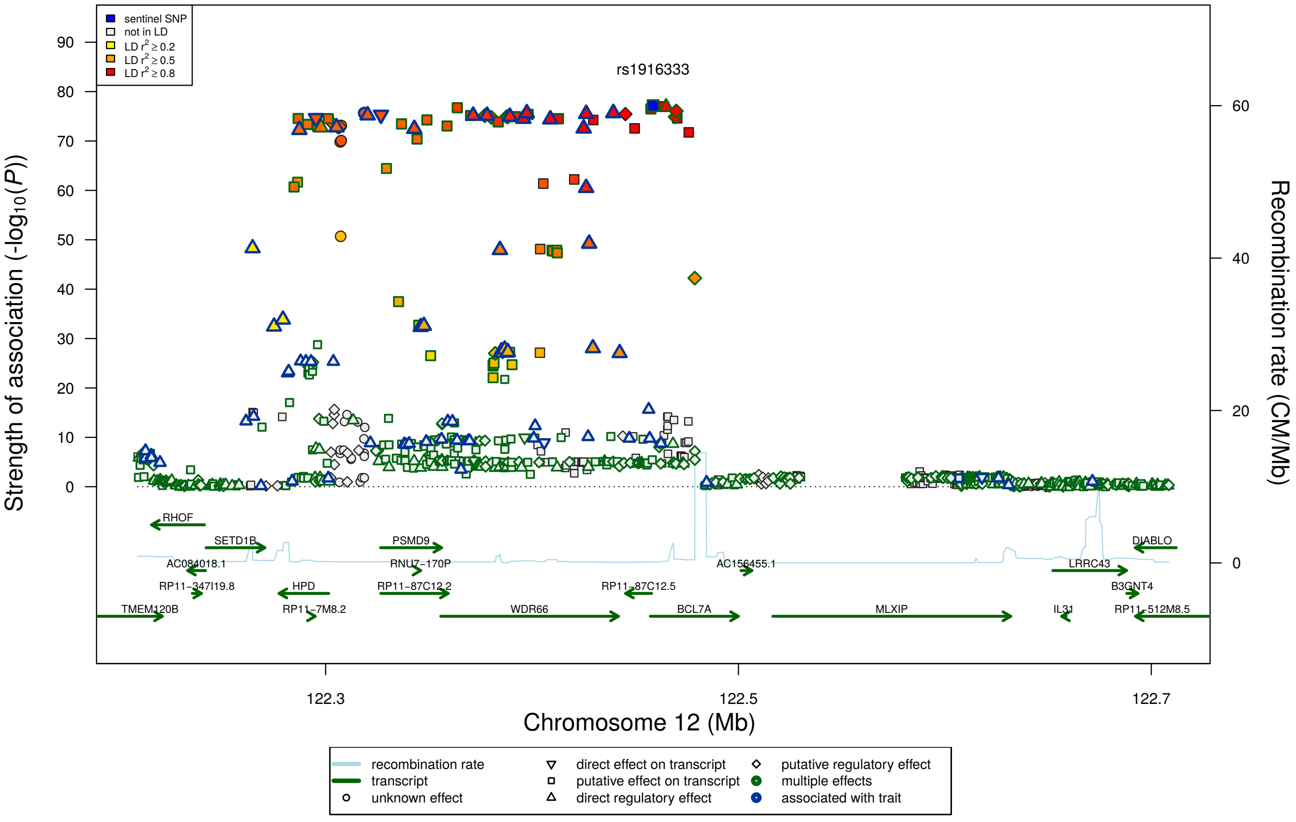

Genotype of rs1916333

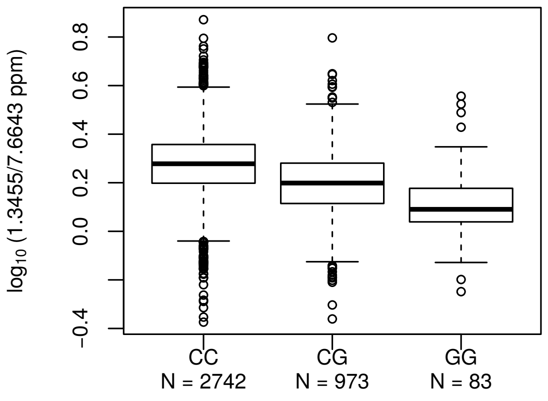

Genome−wide associations with 1.3455/7.6643 ppm

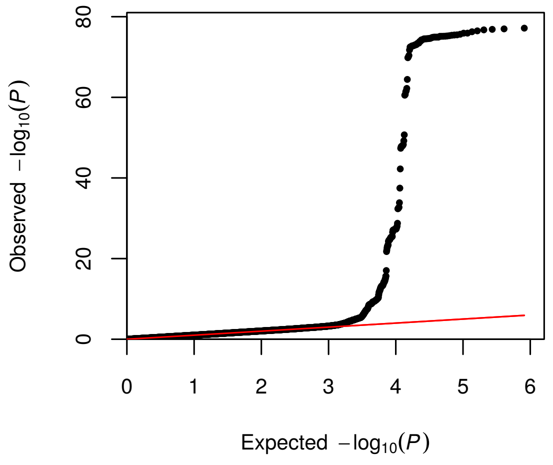

GWAS with **non-targeted** metabolic traits - **ACSM3** locus

Associations with 1.2565/1.0666 ppm

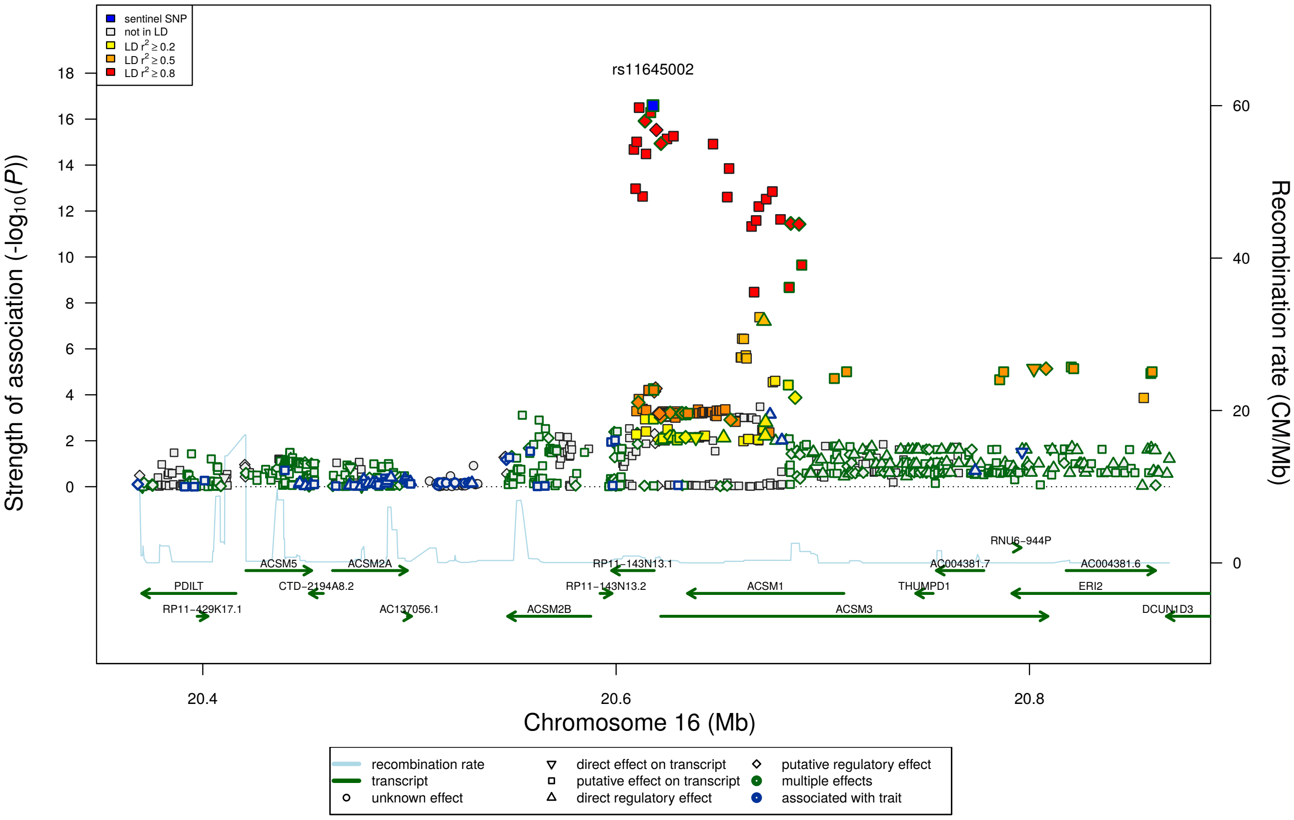

Genotype of rs11645002

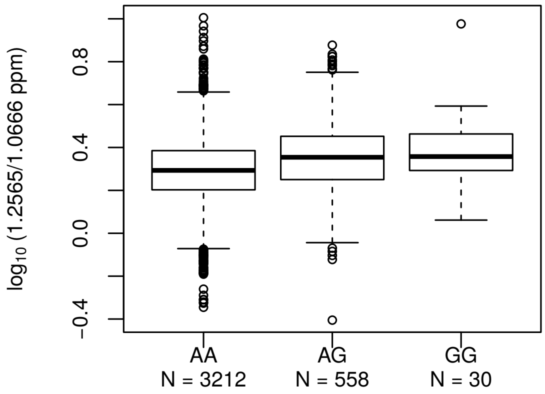

Genome−wide associations with 1.2565/1.0666 ppm

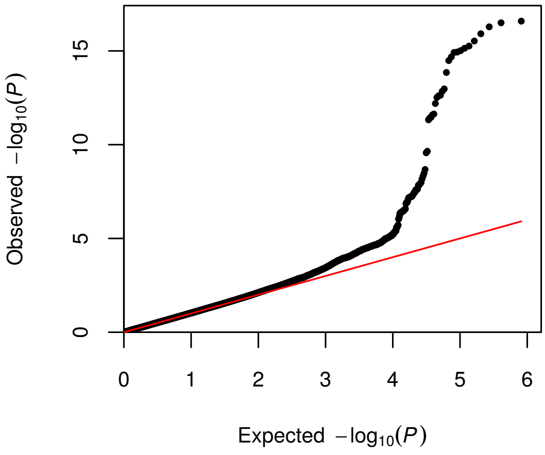

GWAS with **non-targeted** metabolic traits - **SLC5A11** locus

Associations with 3.5944/1.0666 ppm

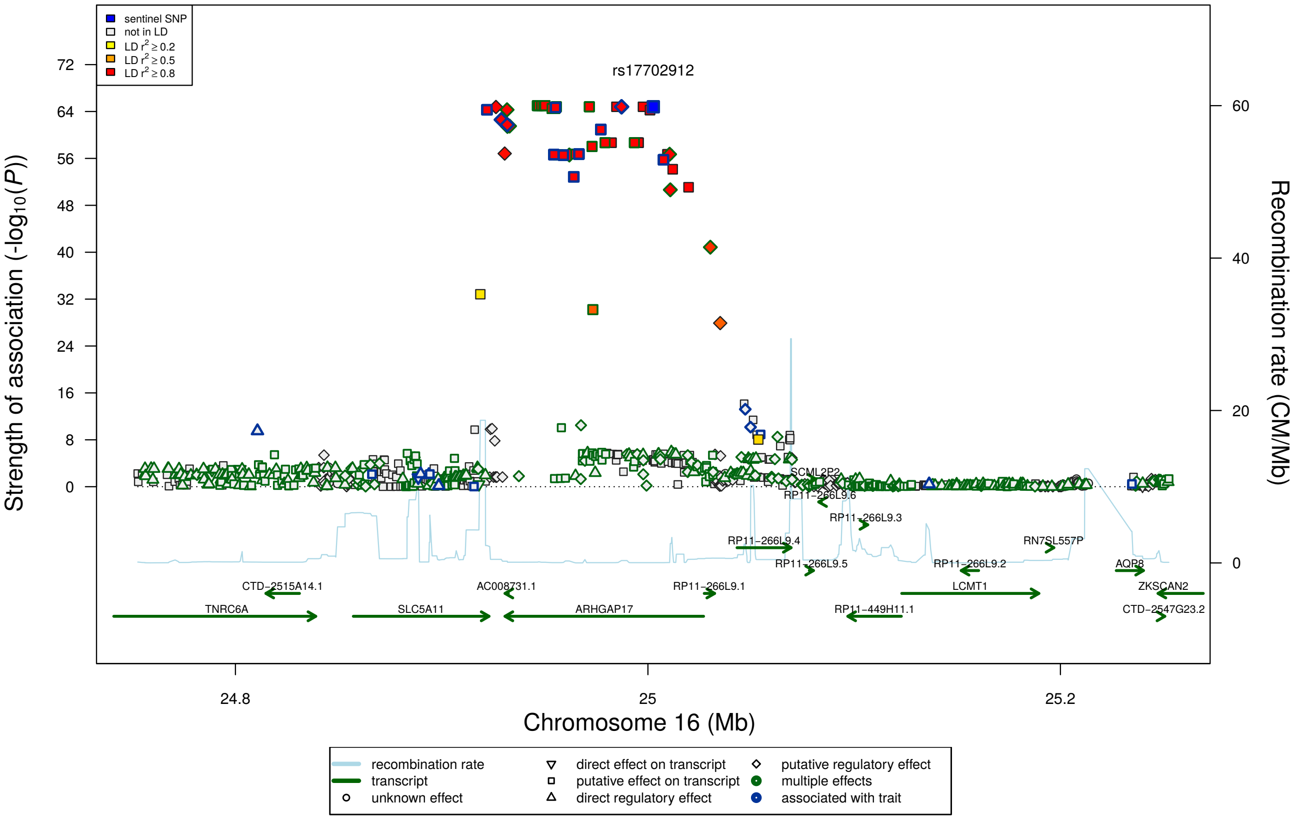

Genotype of rs17702912

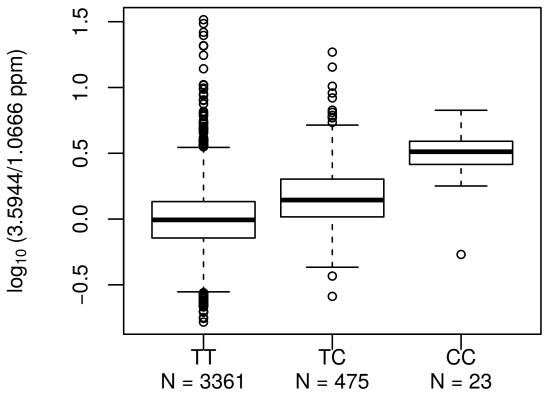

Genome−wide associations with 3.5944/1.0666 ppm

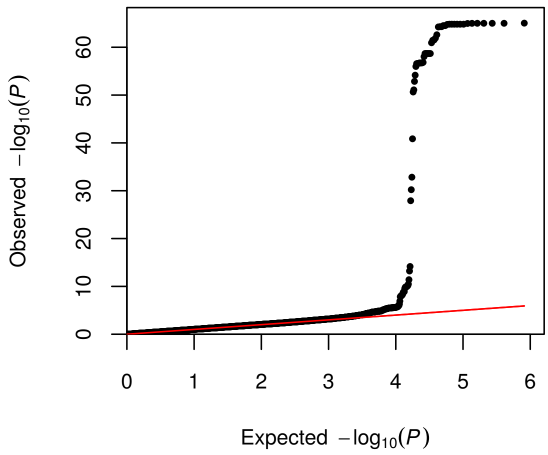

GWAS with **non-targeted** metabolic traits - **PNMT** locus

Associations with 6.8769 ppm

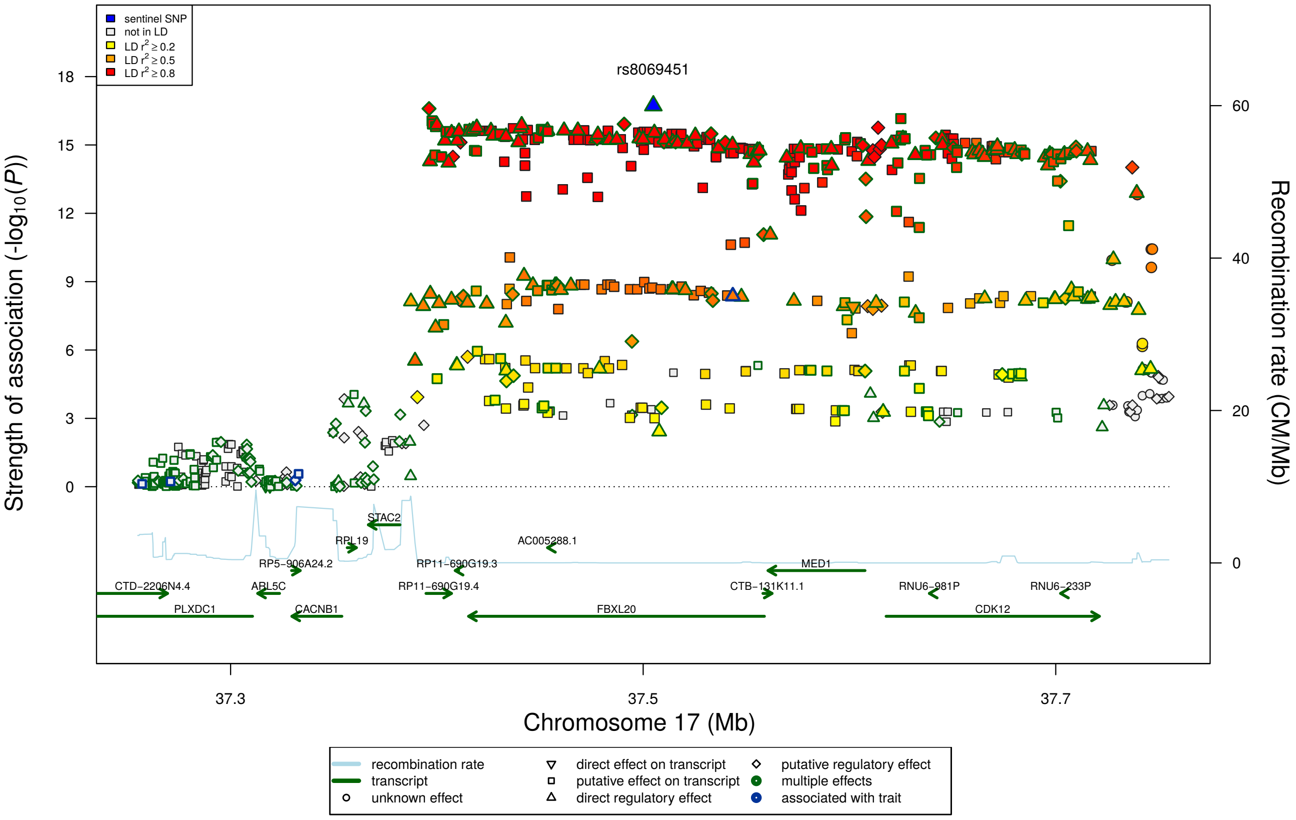

Genotype of rs8069451

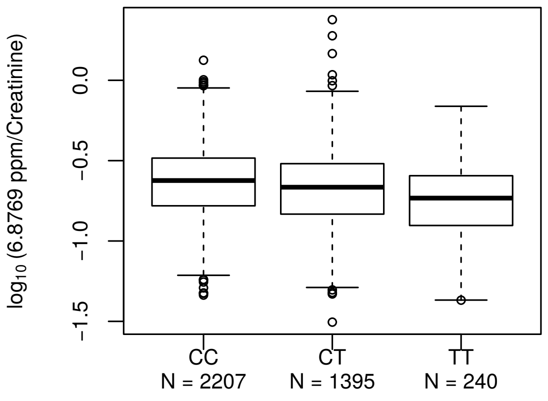

Genome−wide associations with 6.8769 ppm

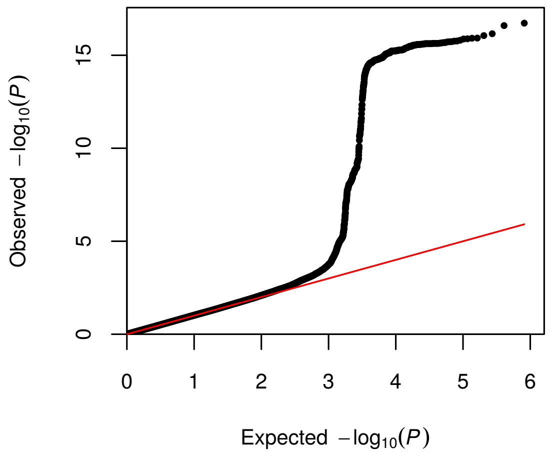

GWAS with **non-targeted** metabolic traits - **SLC13A3** locus

Associations with 2.3946/2.6495 ppm

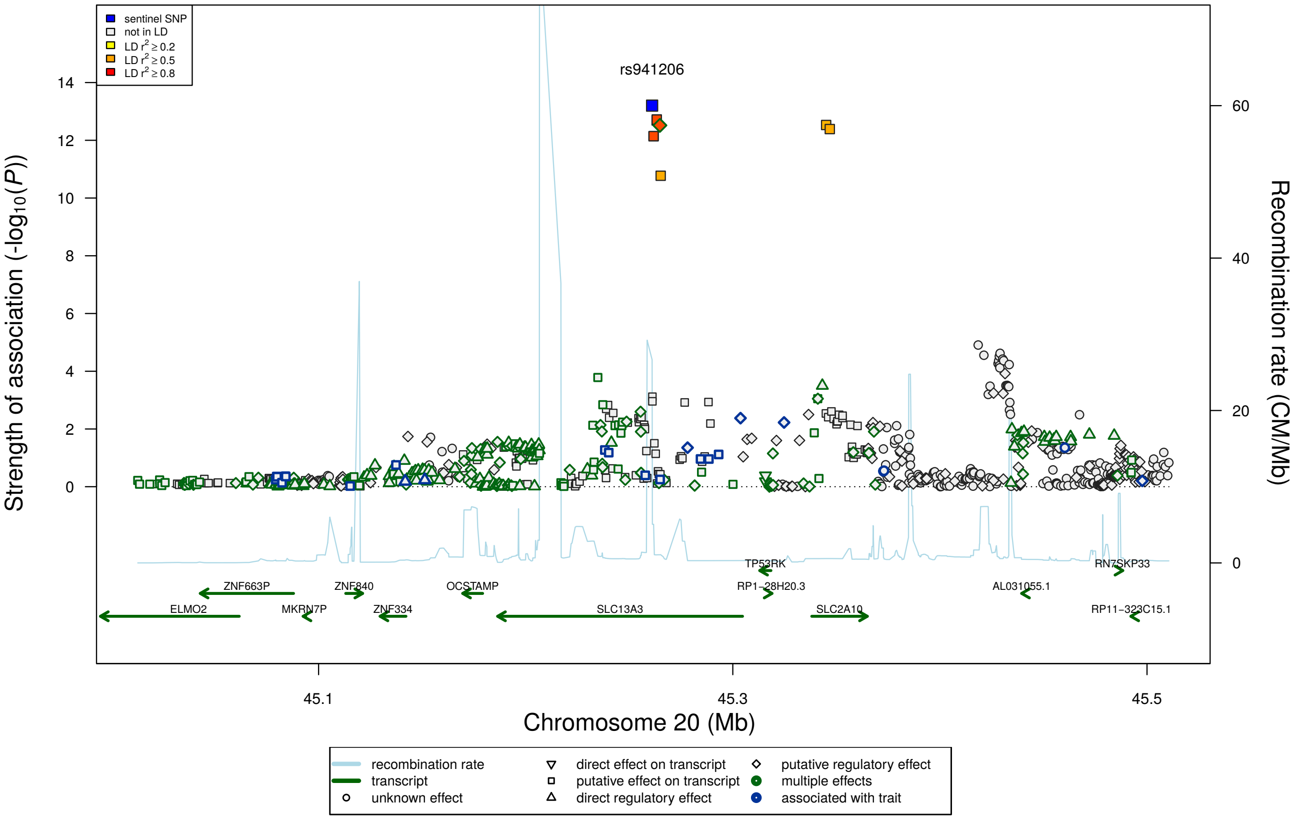

Genotype of rs941206

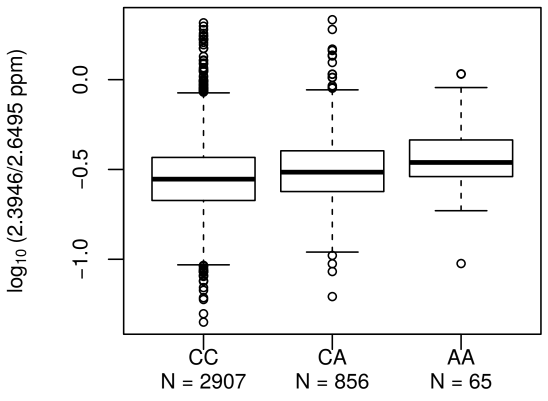

Genome−wide associations with 2.3946/2.6495 ppm

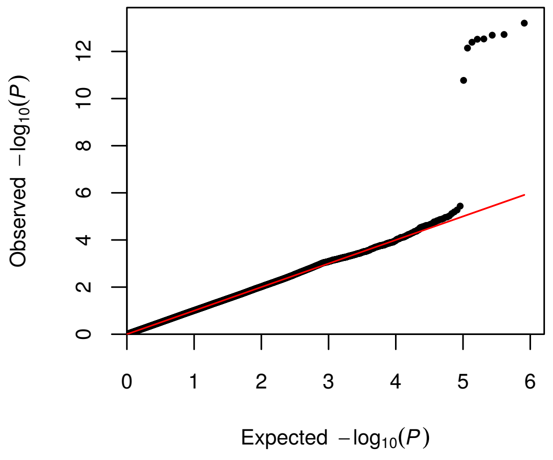

Supplement: S2 Fig — Top: The regional association plot displays SNPs in a locus and the strength of association to the top-associated metabolic trait (Tables 1 and 2). The plot window (500 kb) is centered on the SNP that displays the strongest association signal and, in case of ratios, meets the P-gain criterion (“lead SNP”, indicated in blue). The variants are colored according to the degree of linkage disequilibrium (LD) to the lead SNP. The plot symbols and border colors indicate functional annotations (e.g. effects on transcripts) as well as results from other association studies. Plots were created with the SNiPA web service (available at www.snipa.org) using data from the 1000 genomes project (phase 1 version 3, European super-population) and Ensembl 75. Bottom left: Top associated metabolic trait in SHIP-0 grouped by genotype of the lead SNP (in order major allele homozygotes, heterozygotes, and minor allele homozygotes). Bottom right: Quantile-quantile plots displaying the observed vs. the expected distribution of P-values for associations to the top-associated metabolic trait. (PDF) [file pgen.1005487.s002.pdf]
