# Supplementary figures and images for: Genome-Wide Association Study with Targeted and Non-targeted NMR Metabolomics Identifies 15 Novel Loci of Urinary Human Metabolic Individuality
Source: PLoS Genet. 2015 Sep 9;11(9):e1005487. doi: 10.1371/journal.pgen.1005487 (PMC4564198; doi:10.1371/journal.pgen.1005487)

# Xylulose Pathway

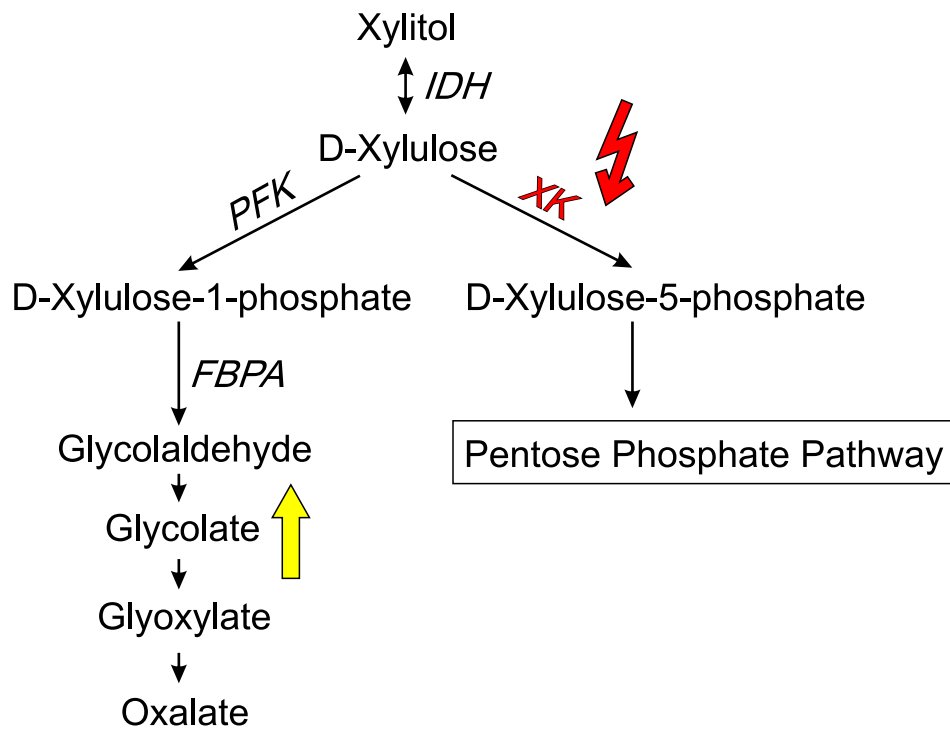

Supplement: S3 Fig — The mGWAS identified significant associations of SNP rs3132440 (intronic region of XYLB) and rs17118 (non-synonymous variant in XYLB) with glycolate. The urinary concentration increases per copy of the effect allele (indicated by the yellow arrow). Glycolate is a downstream product of the PFK-mediated D-xylulose pathway. Thus, the associated variants could decrease the enzymatic activity of XYLB’s gene product (xylulokinase; XK), which would lead to an increased D-xylulose metabolism via PFK. Figure adapted from [70, 91]. (PDF) [file pgen.1005487.s003.pdf]

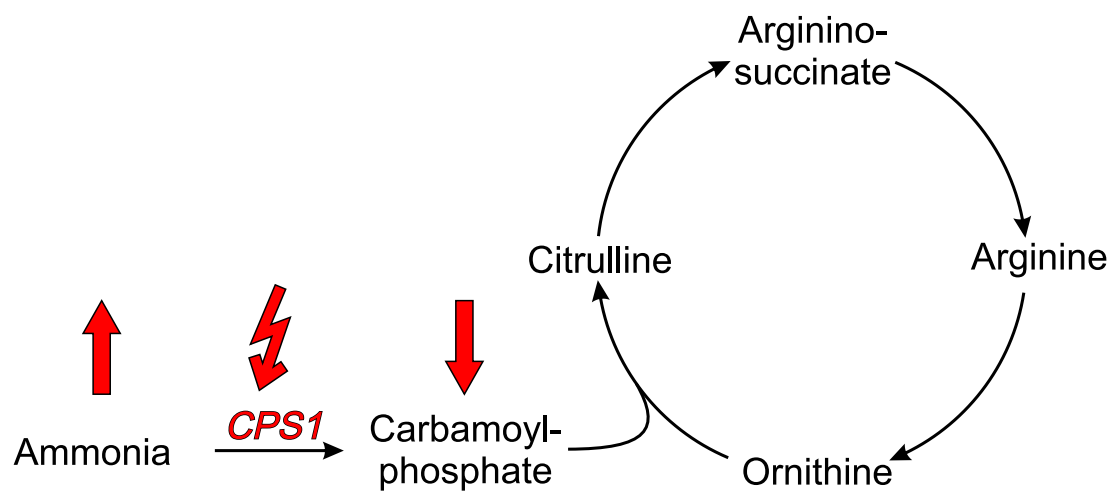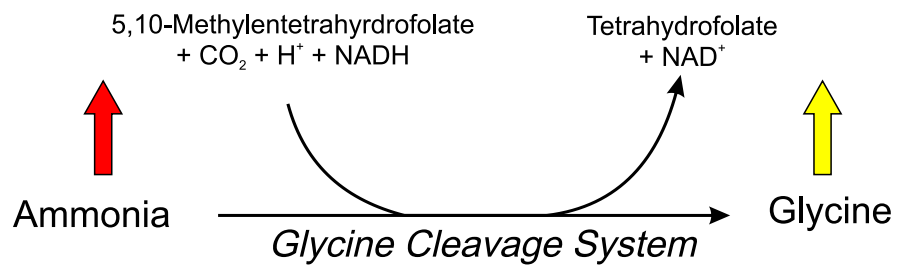

Supplement: S4 Fig — A deficiency of Carbamoyl-Phosphate Synthetase 1 (CPS1) can lead to high ammonia levels in blood (top). We detected significant associations of variants in CPS1 and elevated urinary glycine levels (indicated by the yellow arrow). We hypothesize that the excess ammonia is converted to glycine via the Glycine Cleavage System (bottom). (PDF) [file pgen.1005487.s004.pdf]
