# Supplementary material for: Genome-Wide Association Study with Targeted and Non-targeted NMR Metabolomics Identifies 15 Novel Loci of Urinary Human Metabolic Individuality
Source: PLoS Genet. 2015 Sep 9;11(9):e1005487. doi: 10.1371/journal.pgen.1005487 (PMC4564198; doi:10.1371/journal.pgen.1005487)
Supplement: S1 Table — In case of association to ratios, the metabolite that shows the stronger association signal is listed in the numerator. Tests: number of conducted association tests on the SNP. The significance level was adjusted for 38 tests (P < 0.05/38 = 1.32×10^−3^), which is the sum of replication attempts using targeted traits and non-targeted traits (S2 Table). In the targeted data set, the strongest identified association between rs7247977 (SLC7A9) and the lysine/valine ratio could not be replicated. Instead, the second-strongest, still significant association of this SNP with the concentrations of valine was successfully replicated. (DOCX) [file pgen.1005487.s005.docx]

| **Genetic data** | |  | **Associated targeted metabolic trait** | | | | |
| --- | --- | --- | --- | --- | --- | --- | --- |
| **Locus** | **SNP** |  | **Trait or pairwise ratio** | **Tests** | **N** | **beta’** | ***P*** |
| *CPS1* | rs715 |  | glycine/threonine | 1 | 1,559 | 0.1136 | 2.49×10^-11^ |
| *XYLB* | rs3132440 |  | glycolate | 1 | 1,577 | 0.0792 | 1.82×10^-7^ |
| *SLC6A20* | rs17279437 |  | N,N-dimethylglycine/alanine | 1 | 1,581 | -0.2575 | 1.32×10^-18^ |
| *ETNPPL* | rs56043887 |  | ethanolamine | 1 | 1,510 | -0.0317 | 2.14×10^-4^ |
| *SLC6A19* | rs11133665 |  | histidine/τ-methylhistidine | 1 | 1,176 | -0.1475 | 3.29×10^-11^ |
| *AGXT2* | rs37369 |  | 3-aminoisobutyrate | 1 | 1,394 | 2.4578 | 1.39×10^-85^ |
| *DMGDH* | rs6453429 |  | N,N-dimethylglycine/betaine | 1 | 1,495 | 0.1478 | 1.31×10^-8^ |
| *SLC36A2* | rs3846710 |  | glycine/citrate | 1 | 1,679 | -0.1009 | 1.09×10^-5^ |
| *NAT2* | rs1495743 |  | formate/acetate | 1 | 1,399 | 0.1132 | 4.17×10^-5^ |
| *GLDC* | rs1755615 |  | glycine/alanine | 1 | 1,638 | 0.0696 | 3.23×10^-4^ |
| *SLC6A13* | rs11062102 |  | 3-aminoisobutyrate | 1 | 1,362 | -0.1869 | 4.35×10^-8^ |
| *HPD* | rs4760099 |  | 2-hydroxyisobutyrate | 1 | 1,672 | -0.1568 | 7.25×10^-27^ |
| *PNMT* | rs7219014 |  | histidine/τ-methylhistidine | 1 | 1,167 | -0.1159 | 6.85×10^-7^ |
| *SLC7A9* | rs7247977 |  | lysine | 2 | 182 | 0.2656 | 7.10×10^-4^ |
| *SLC13A3* | rs941206 |  | succinate/citrate | 1 | 1,526 | 0.1203 | 4.17×10^-4^ |
