# Supplementary material for: Genome-Wide Association Study with Targeted and Non-targeted NMR Metabolomics Identifies 15 Novel Loci of Urinary Human Metabolic Individuality
Source: PLoS Genet. 2015 Sep 9;11(9):e1005487. doi: 10.1371/journal.pgen.1005487 (PMC4564198; doi:10.1371/journal.pgen.1005487)
Supplement: S2 Table — Non-targeted traits are reported as chemical shifts (i.e., the position in the NMR spectrum). To replicate the non-targeted association results, we selected the NMR peaks or ratios thereof that were closest to the NMR features used in the SHIP-0 data set. For five loci (ETNPPL, SLC6A19, DMGDH, ABO, and PNMT), we were unable to replicate the top associations in the KORA F4 data set. These associations are marked with an asterisk (*). (DOCX) [file pgen.1005487.s006.docx]

| **Genetic data** | |  | **Associated non-targeted metabolic trait** | | | | |
| --- | --- | --- | --- | --- | --- | --- | --- |
| **Locus** | **SNP** |  | **Trait or pairwise ratio** | **Tests** | **N** | **beta’** | ***P*** |
| *NAT8* | rs10178409 |  | 2.031 ppm | 1 | 1,675 | 0.1266 | 4.08×10^-33^ |
| *HIBCH* | rs13006833 |  | 1.067 ppm/1.050 ppm | 1 | 1,582 | 0.0316 | 1.41×10^-12^ |
| *CPS1* | rs715 |  | 3.558 ppm/2.548 ppm | 1 | 1,600 | 0.1752 | 1.83×10^-13^ |
| *AGXT* | rs6748734 |  | 1.085 ppm | 1 | 1,672 | 0.0688 | 1.32×10^-7^ |
| *SLC6A20* | rs17279437 |  | 2.915 ppm | 1 | 1,689 | -0.2149 | 1.65×10^-9^ |
| *TKT* | rs4687717 |  | 4.085 ppm / 7.664 ppm | 1 | 1,543 | 0.0357 | 1.34×10^-4^ |
| *ETNPPL* | rs7437890 |  | 3.125 ppm | 1 | 1,678 | 0.0324 | 0.0312 * |
| *SLC6A19* | rs11750211 |  | 6.861 ppm | 1 | 1,575 | 0.0178 | 0.421 * |
| *AGXT2* | rs37369 |  | 1.172 ppm/1.973 ppm | 1 | 1,685 | 1.9289 | 1.01×10^-132^ |
| *DMGDH* | rs6453427 |  | 2.915 ppm/5.235 ppm | 1 | 1,639 | 0.0328 | 0.276 * |
| *SLC36A2* | rs3846710 |  | 3.558 ppm/2.654 ppm | 1 | 1,690 | -0.0983 | 4.79×10^-5^ |
| *NAT2* | rs35246381 |  | 2.159 ppm/3.320 ppm | 1 | 1,682 | 0.6252 | 6.95×10^-70^ |
| *ABO* | rs550057 |  | 2.031 ppm/2.049 ppm | 2 | 1,666 | -0.0122 | 0.150 * |
| *PYROXD2* | rs11598867 |  | 2.854 ppm | 1 | 1,468 | -0.2369 | 3.34×10^-177^ |
| *SLC6A13* | rs11062102 |  | 1.190 ppm | 1 | 1,637 | -0.1286 | 8.47×10^-5^ |
| *HPD* | rs1916333 |  | 1.346 ppm/7.664 ppm | 1 | 1,645 | -0.1309 | 2.52×10^-16^ |
| *ACSM3* | rs11645002 |  | 1.257 ppm/1.067 ppm | 1 | 1,667 | 0.1532 | 1.07×10^-11^ |
| *SLC5A11* | rs17702912 |  | 3.594 ppm/1.067 ppm | 1 | 1,690 | 0.4250 | 4.57×10^-24^ |
| *PNMT* | rs8069451 |  | 6.861 ppm | 1 | 1,684 | 0.0072 | 0.734 * |
| *SLC13A3* | rs941206 |  | 2.396 ppm/2.654 ppm | 2 | 1,678 | 0.1065 | 8.55×10^-4^ |
