# Supplementary material for: Genome-Wide Association Study with Targeted and Non-targeted NMR Metabolomics Identifies 15 Novel Loci of Urinary Human Metabolic Individuality
Source: PLoS Genet. 2015 Sep 9;11(9):e1005487. doi: 10.1371/journal.pgen.1005487 (PMC4564198; doi:10.1371/journal.pgen.1005487)
Supplement: S4 Table — Expression rate (given as reads per kilobase of transcript per million mapped reads; RPKM) for the most likely effector gene per locus as determined in 16 different tissues. (DOCX) [file pgen.1005487.s008.docx]

| **Genes** | **Lung** | **Lymph Node** | **Prostate** | **Skeletal Muscle** | **White Blood Cells** | **Ovary** | **Testes** | **Thyroid** | **Kidney** | **Heart** | **Liver** | **Adipose** | **Adrenal** | **Brain** | **Breast** | **Colon** |
| --- | --- | --- | --- | --- | --- | --- | --- | --- | --- | --- | --- | --- | --- | --- | --- | --- |
| NAT8 | 0.24 | 0.06 | 0.09 | 0.00 | 0.32 | 0.31 | 0.09 | 0.00 | 63.67 | 0.00 | 5.58 | 0.00 | 0.00 | 0.09 | 0.00 | 0.00 |
| HIBCH | 7.88 | 10.11 | 20.25 | 4.20 | 6.24 | 16.96 | 25.68 | 29.11 | 45.55 | 13.71 | 27.98 | 12.38 | 8.36 | 8.56 | 30.48 | 22.53 |
| CPS1 | 0.58 | 0.67 | 0.71 | 0.25 | 0.05 | 1.39 | 5.26 | 0.82 | 1.57 | 0.50 | 306.77 | 0.83 | 0.48 | 0.78 | 1.43 | 0.59 |
| AGXT | 0.01 | 0.06 | 0.00 | 0.02 | 0.00 | 0.01 | 0.00 | 0.00 | 0.54 | 0.00 | 585.76 | 0.09 | 0.00 | 0.00 | 0.43 | 0.00 |
| XYLB | 0.13 | 0.40 | 0.89 | 0.12 | 0.36 | 0.92 | 0.57 | 0.12 | 1.90 | 0.24 | 7.81 | 0.58 | 0.48 | 0.32 | 0.88 | 0.61 |
| SLC6A20 | 0.28 | 0.04 | 0.06 | 0.10 | 0.05 | 0.04 | 0.10 | 0.14 | 1.07 | 0.00 | 0.00 | 0.50 | 0.02 | 0.41 | 0.01 | 0.06 |
| TKT | 72.76 | 39.67 | 85.49 | 6.65 | 182.63 | 57.00 | 26.92 | 86.24 | 72.17 | 5.77 | 24.58 | 94.27 | 31.93 | 26.40 | 131.21 | 62.52 |
| ETNPPL | 0.07 | 0.00 | 0.05 | 0.55 | 0.00 | 0.04 | 0.26 | 0.33 | 0.19 | 2.67 | 25.55 | 0.02 | 0.04 | 59.79 | 0.00 | 0.00 |
| SLC6A19 | 0.02 | 0.00 | 0.00 | 0.00 | 0.00 | 0.00 | 0.17 | 0.00 | 5.31 | 0.00 | 0.41 | 0.03 | 0.00 | 0.00 | 0.00 | 0.00 |
| AGXT2 | 0.00 | 0.06 | 0.00 | 0.01 | 0.00 | 0.00 | 0.07 | 0.00 | 23.96 | 0.00 | 23.34 | 0.00 | 0.00 | 0.00 | 0.03 | 0.00 |
| DMGDH | 0.09 | 0.25 | 1.08 | 0.36 | 0.00 | 1.25 | 1.40 | 2.56 | 19.01 | 0.45 | 14.67 | 1.16 | 0.50 | 0.92 | 6.58 | 0.90 |
| SLC36A2 | 0.02 | 0.18 | 0.04 | 9.96 | 0.00 | 0.01 | 0.37 | 1.84 | 11.92 | 0.13 | 0.00 | 0.08 | 0.03 | 0.01 | 0.64 | 0.00 |
| NAT2 | 0.00 | 0.00 | 0.05 | 0.00 | 0.00 | 0.11 | 0.22 | 0.10 | 0.23 | 0.09 | 37.96 | 0.05 | 0.03 | 0.00 | 0.19 | 0.74 |
| GLDC | 0.20 | 0.89 | 0.38 | 0.13 | 0.12 | 0.14 | 2.89 | 5.21 | 20.47 | 0.03 | 22.66 | 0.07 | 0.43 | 2.77 | 0.12 | 0.03 |
| PYROXD2 | 2.22 | 1.42 | 3.19 | 0.13 | 0.76 | 5.33 | 0.80 | 3.68 | 1.87 | 3.40 | 0.30 | 2.04 | 1.41 | 0.97 | 7.53 | 0.97 |
| SLC6A13 | 0.19 | 0.06 | 0.04 | 0.04 | 0.08 | 0.08 | 0.57 | 1.97 | 23.83 | 0.16 | 1.79 | 0.00 | 0.09 | 1.23 | 0.02 | 0.00 |
| HPD | 0.70 | 0.57 | 4.95 | 0.35 | 0.08 | 3.05 | 3.58 | 1.09 | 50.50 | 0.09 | 561.54 | 0.08 | 1.37 | 0.86 | 2.28 | 0.50 |
| ACSM3 | 1.63 | 0.77 | 3.73 | 0.26 | 1.89 | 9.89 | 2.53 | 0.55 | 24.71 | 0.63 | 16.16 | 0.32 | 0.61 | 0.15 | 0.75 | 3.31 |
| SLC5A11 | 0.11 | 0.21 | 0.21 | 0.00 | 0.00 | 0.01 | 0.15 | 0.10 | 0.17 | 0.01 | 0.02 | 0.01 | 0.25 | 8.60 | 0.00 | 0.07 |
| PNMT | 0.42 | 0.37 | 2.21 | 1.62 | 0.00 | 0.04 | 0.92 | 0.69 | 1.24 | 1.64 | 0.08 | 0.50 | 0.20 | 1.30 | 0.12 | 1.02 |
| SLC7A9 | 0.14 | 0.51 | 0.32 | 0.00 | 0.00 | 0.53 | 0.23 | 0.17 | 7.86 | 0.04 | 5.28 | 0.02 | 0.04 | 0.09 | 0.13 | 0.11 |
| SLC13A3 | 0.77 | 0.27 | 0.82 | 0.10 | 0.00 | 1.19 | 0.79 | 0.49 | 162.62 | 0.16 | 5.24 | 0.47 | 0.41 | 3.94 | 0.25 | 0.45 |
